# Burosumab Improved Histomorphometric Measures of Osteomalacia in Adults with X‐Linked Hypophosphatemia: A Phase 3, Single‐Arm, International Trial

**DOI:** 10.1002/jbmr.3843

**Published:** 2019-10-01

**Authors:** Karl L Insogna, Frank Rauch, Peter Kamenický, Nobuaki Ito, Takuo Kubota, Akie Nakamura, Lin Zhang, Matt Mealiffe, Javier San Martin, Anthony A Portale

**Affiliations:** ^1^ Department of Internal Medicine Yale School of Medicine New Haven CT USA; ^2^ Genetics Unit, Shriners Hospital for Children and McGill University Montreal QC Canada; ^3^ APHP, Department of Endocrinology and Reproductive Diseases, Bicêtre Paris Sud Hospital Le Kremlin Bicêtre France; ^4^ Department of Endocrinology & Nephrology The University of Tokyo Hospital Tokyo Japan; ^5^ Department of Pediatrics Osaka University Hospital Osaka Japan; ^6^ Department of Pediatrics Hokkaido University Hospital Hokkaido Japan; ^7^ Ultragenyx Pharmaceutical Inc. Novato CA USA; ^8^ Division of Pediatric Nephrology University of California San Francisco CA USA

**Keywords:** BIOCHEMICAL MARKERS OF BONE TURNOVER, BONE HISTOMORPHOMETRY, CLINICAL TRIALS, OSTEOMALACIA AND RICKETS, THERAPEUTICS > OTHER

## Abstract

In adults with X‐linked hypophosphatemia (XLH), excess FGF23 impairs renal phosphate reabsorption and suppresses production of 1,25‐dihydroxyvitamin D, resulting in chronic hypophosphatemia and persistent osteomalacia. Osteomalacia is associated with poor bone quality causing atraumatic fractures, pseudofractures, delayed fracture healing, and bone pain. Burosumab is a fully human monoclonal antibody against FGF23. UX023‐CL304 is an ongoing, open‐label, single‐arm, phase 3 study investigating the efficacy of subcutaneous burosumab, 1.0 mg/kg administered every 4 weeks, in improving osteomalacia in adults with XLH who have not been treated for at least 2 years before enrollment. The primary endpoint was improvement in osteoid volume/bone volume assessed by transiliac bone biopsies obtained at baseline and week 48. Additional assessments included serum phosphorus, markers of bone turnover, fracture/pseudofracture healing, and safety. Fourteen subjects enrolled, 13 completed 48 weeks, and 11 completed paired biopsies. All osteomalacia‐related histomorphometric measures improved significantly at week 48 (mean percent change: osteoid volume/bone volume, –54%, osteoid thickness, –32%, osteoid surface/bone surface, –26%, [median] mineralization lag time, –83%). Mean serum phosphorus concentration averaged across the mid‐point of the dose cycle between weeks 0 and 24 was 3.3 mg/dL, a 50% increase from 2.2 mg/dL at baseline. Markers of bone formation and resorption increased at week 48 (least squares [LS] mean increase: P1NP, +77%; CTx, +36%; both *p* < 0.0001). All subjects had one or more treatment‐emergent adverse event (AE). Most AEs were mild to moderate in severity. Two subjects experienced serious AEs (migraine; paresthesia) that were unrelated to treatment and resolved. Eleven subjects had 18 biopsy procedure‐related AEs: 14 for pain, two for itch, and one each for headache and bandage irritation. No deaths or incidents of hyperphosphatemia occurred. In conclusion, by normalizing phosphate homeostasis, burosumab significantly improved osteomalacia in adults with XLH, which likely explains the improved fracture healing and amelioration of skeletal complications. © 2019 The Authors. *Journal of Bone and Mineral Research* published by American Society for Bone and Mineral Research.

## Introduction

X‐linked hypophosphatemia (XLH) is a rare, lifelong disease caused by loss of function mutations in the *PHEX* (phosphate‐regulating endopeptidase homolog, X‐linked) gene, resulting in increased circulating levels of FGF23 that lead to chronic hypophosphatemia and impaired production of 1,25‐dihydroxyvitamin D (1,25(OH)_2_D).^1^, ^2^ Low serum phosphorus levels result in rickets and osteomalacia, the hallmarks of XLH in children and adults, respectively. Osteomalacia is associated with poor bone quality that results in pseudofractures, fractures, impaired fracture healing, and bone and joint pain.^3^, ^4^, ^5^

Since the 1980s, conventional therapy for XLH has consisted of multiple daily doses of oral phosphate and one or more doses of an active vitamin D metabolite.^1^, ^2^ Although this therapy has been the standard of care for children with XLH, there is no consensus regarding its use in adults. This is primarily because of concerns about its long‐term risks including nephrocalcinosis, hypercalciuria, and hyperparathyroidism. Previous studies have demonstrated improvement, albeit not complete healing of osteomalacia with conventional therapy in adults.^6^, ^7^, ^8^ Those studies used histomorphometric indices, such as the ratio of osteoid volume to total bone volume, in iliac crest bone biopsies to evaluate changes in the severity of osteomalacia.^9^, ^10^

Burosumab is a fully human monoclonal antibody against FGF23 approved for the treatment of XLH (in the US, EU, and Canada, with conditions of approval varying per location).^11^ In a phase 3 study in adults with XLH, burosumab significantly improved fracture healing, increased serum phosphorus levels and biochemical markers of bone remodeling, and reduced patient‐reported stiffness compared to placebo.^12^, ^13^ In the present study, we investigated the effect of treatment with burosumab on histomorphometric indices of osteomalacia in adults with XLH.

## Subjects and Methods

### Study design

UX023‐CL304 (http://clinicaltrials.gov identifier NCT02537431) is an ongoing, phase 3, single‐arm, multicenter trial investigating the efficacy of burosumab in improving osteomalacia in adults with XLH. Subjects were treated with burosumab, 1.0 mg/kg (rounded to the nearest 10 mg) administered subcutaneously every 4 weeks (Q4W) for at least 96 weeks. Transiliac crest bone biopsies—horizontal full‐thickness biopsies of the ilium from a site 2 cm dorsal of the anterior superior iliac spine—were obtained at baseline and week 48 using a needle with an inner diameter of at least 5 mm; the protocol recommended the use of a Bordier needle. Subjects received two 3‐day courses of tetracycline‐HCl (or demeclocycline‐HCl) 20 and 8 days before each biopsy. The use of tetracycline allowed for dynamic histomorphometric analysis, in which unstained sections were mounted unstained for florescence microscopy as described by Glorieux and colleagues.^14^ For structural morphometric analysis, additional sections of the biopsy were also stained using Masson Goldner Trichrome as described.^14^ Biopsies were qualitatively analyzed in real‐time to determine if osteomalacia was present by an experienced histomorphometrist (FR). If the baseline biopsy did not reveal osteomalacia in a given subject, they continued treatment but did not undergo a second biopsy at week 48. After week 48, all subjects continued treatment for an additional 48‐week extension period, for a total duration of 96 weeks. This report summarizes data through week 48.

### Participants

Key eligibility criteria included a diagnosis of XLH with documentation of a *PHEX* mutation or a serum intact FGF23 level greater than 30 pg/mL; an age between18 and 65 years; a fasting serum phosphorus and renal tubular maximum reabsorption of phosphate per glomerular filtration rate (TmP/GFR) <2.5 mg/dL; and the presence of skeletal pain defined as a score of four or more on Question 3, “Worst Pain,” of the Brief Pain Inventory.^15^ Subjects were ineligible to participate if they received conventional therapy within 2 years prior to screening. Additional inclusion and exclusion criteria are provided in the Supplementary Materials.

This study was designed, conducted, recorded, and reported in accordance with the principles established by the World Medical Association Declaration of Helsinki Ethical Principles for Medical Research Involving Human Subjects. The Institutional Review Board or Ethics Committee for each site approved the study protocol. Investigators obtained written informed consent from each study participant.

### Outcomes

Bone samples were processed and histomorphometric analyzes were performed as described in the study design section and as reported.^14^ The primary endpoint was the percentage change from baseline to week 48 in osteoid volume/bone volume. Changes in osteoid thickness, osteoid surface/bone surface, and mineralization lag time were also quantified. Mineralization lag time refers to the average time interval between osteoid formation and its subsequent mineralization and is calculated by dividing the osteoid thickness by the adjusted apposition rate. In subjects in whom the mineralization defect was profound and therefore the uptake of tetracycline label very low, mineralization lag time was calculated using an established technique.^16^ The structural histomorphometric parameters measured were cancellous bone volume/total volume, trabecular thickness, and cortical width. In addition to mineralization lag time, dynamic remodeling parameters assessed were mineralizing surface/bone surface, mineralizing surface/osteoid surface, and mineral apposition rate.

Pharmacodynamic measures of efficacy included change in fasting serum phosphorus, TmP/GFR, and serum 1,25(OH)_2_D levels. The following biochemical markers of bone remodeling were assessed: procollagen type 1 N‐propeptide (P1NP), carboxy‐terminal cross‐linked telopeptide of type I collagen (CTx), and bone‐specific alkaline phosphatase (BALP). Details regarding the schedule for each assessment are available in the Supplemental Materials.

Active (unhealed) fractures and pseudofractures were identified at baseline by a skeletal survey, and fracture healing was assessed by follow‐up targeted X‐rays of those fractures/pseudofractures identified at baseline. Pseudofractures, or Looser zones, are incomplete fractures that typically occur in the absence of a trauma or a fall; pseudofractures involve only one cortex of the long bone but can progress to a complete fracture, defined as a fracture that extends across the entire cortex. Pain and fatigue were assessed based on study subject‐responses to the Brief Pain Inventory and the Brief Fatigue Inventory, respectively.^15^, ^17^, ^18^

The following safety assessments were performed: the incidence and severity of adverse events and serious adverse events, development of anti‐burosumab antibodies, changes in echocardiograms (ECHO) and electrocardiograms (ECG), renal ultrasound nephrocalcinosis scores, fasting serum calcium, plasma intact parathyroid hormone (iPTH), and 24‐hour urine calcium excretion.

### Statistical analysis

Statistical analyses were conducted in SAS version 9.4 or higher (SAS Institute, Cary, NC, USA). Descriptive summaries for efficacy and safety endpoints were provided. Continuous variables were summarized by number of subjects, mean, standard deviation (SD), median, minimum, and maximum. Categorical data were summarized as the total number or percentage of subjects. Histomorphometric endpoints were analyzed using a two‐sided *t* test. If the normal assumption was not met, a sign test for median was used. The 95% CI and *p* value were provided. For other selected endpoints, the least squares (LS) mean and standard error (SE) for the change from baseline to week 48 were provided using the generalized estimating equation (GEE) repeated‐measures analysis, including time as the categorical variable adjusted for baseline measurement in the model with compound symmetry covariance structure.

## Results

### Disposition and baseline characteristics

Of the 25 subjects screened, 14 were enrolled, 13 completed the week 48 study visit, and 11 underwent paired bone biopsies (Supplemental Fig. S1). Three subjects did not undergo a bone biopsy at week 48; one subject withdrew from the study at week 44, and baseline biopsies in two subjects were of insufficient quality to conduct histomorphometric analysis, and therefore, the presence of osteomalacia could not be confirmed (per the protocol subjects without demonstration of osteomalacia at baseline did not undergo biopsy at week 48). Baseline demographics in the subjects who completed paired bone biopsies were similar to those in the group as a whole.

At baseline, study subjects had the typical clinical, biochemical, and radiographic findings of an adult population with XLH that included: bowing of either the upper, lower, or both extremities in all subjects; radiographic evidence of a healed fracture in six (43%) subjects, active pseudofractures in four (29%) subjects, osteoarthritis in eight (57%) subjects, dental disease in 13 (93%) subjects, and prior orthopedic surgery in 11 (79%) subjects. At some point in the past that was at least 2 years before enrollment, 12 (86%) subjects had taken conventional therapy (both oral phosphate salts and active vitamin D metabolites), one (7%) subject had taken only active vitamin D metabolites, and one (7%) subject had not taken any conventional therapy. Most subjects received conventional therapy before the age of 18 years, with the mean duration of oral phosphate and active vitamin D being 13 and 15 years, respectively.

The mean serum phosphorus level in the group was below the normal range of 2.5 to 4.5 mg/dL, consistent with study entry criteria. Osteomalacia was present on the baseline bone biopsy of 11 subjects (Table 1). Due to profound mineralization defects in five subjects, baseline mineralization lag time was calculated using imputation (see Subjects and Methods).^16^

**Table 1:** Baseline Characteristics in 14 Subjects

Characteristic	Total
Age (years), mean ± SD	40.1 ± 8.7
Female, *n* (%)	8 (57.1)
Primary race, *n* (%)	
Asian	4 (28.6)
Black or African American	1 (7.1)
White	9 (64.3)
Weight (kg), mean ± SD	70.3 ± 22.0
Height (cm), mean ± SD	150.4 ± 9.0
Body mass index (kg/m^2^), mean ± SD	30.8 ± 8.5
Serum phosphorus (mg/dL), mean ± SD	2.2 ± 0.4
TmP/GFR (mg/dL), mean (SD)	1.9 ± 0.3
Serum 1,25(OH)_2_D (mg/dL), mean ± SD^a^	37 ± 12

a *n* = 12.

TmP/GFR = renal tubular maximum reabsorption of phosphate per glomerular filtration rate.

### Efficacy

#### Bone histomorphometry

**Figure 1:**
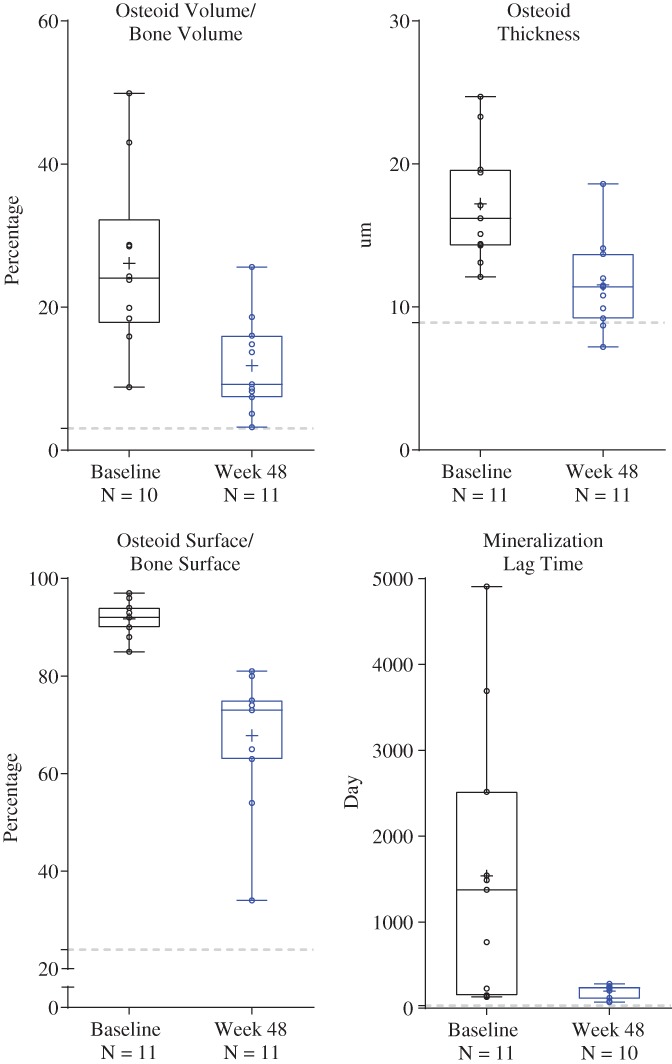
Histomorphometric indices. Data are presented as median, interquartile, range, mean (+), and individual data points (○). Gray line indicates upper limit of normal reference ranges: osteoid volume/bone volume 3.05%, osteoid thickness 8.9 μm, osteoid surface/bone surface 23.9%, mineralization lag time 28.6 days. Data are from Glorieux and colleagues.^14^

**Figure 2:**
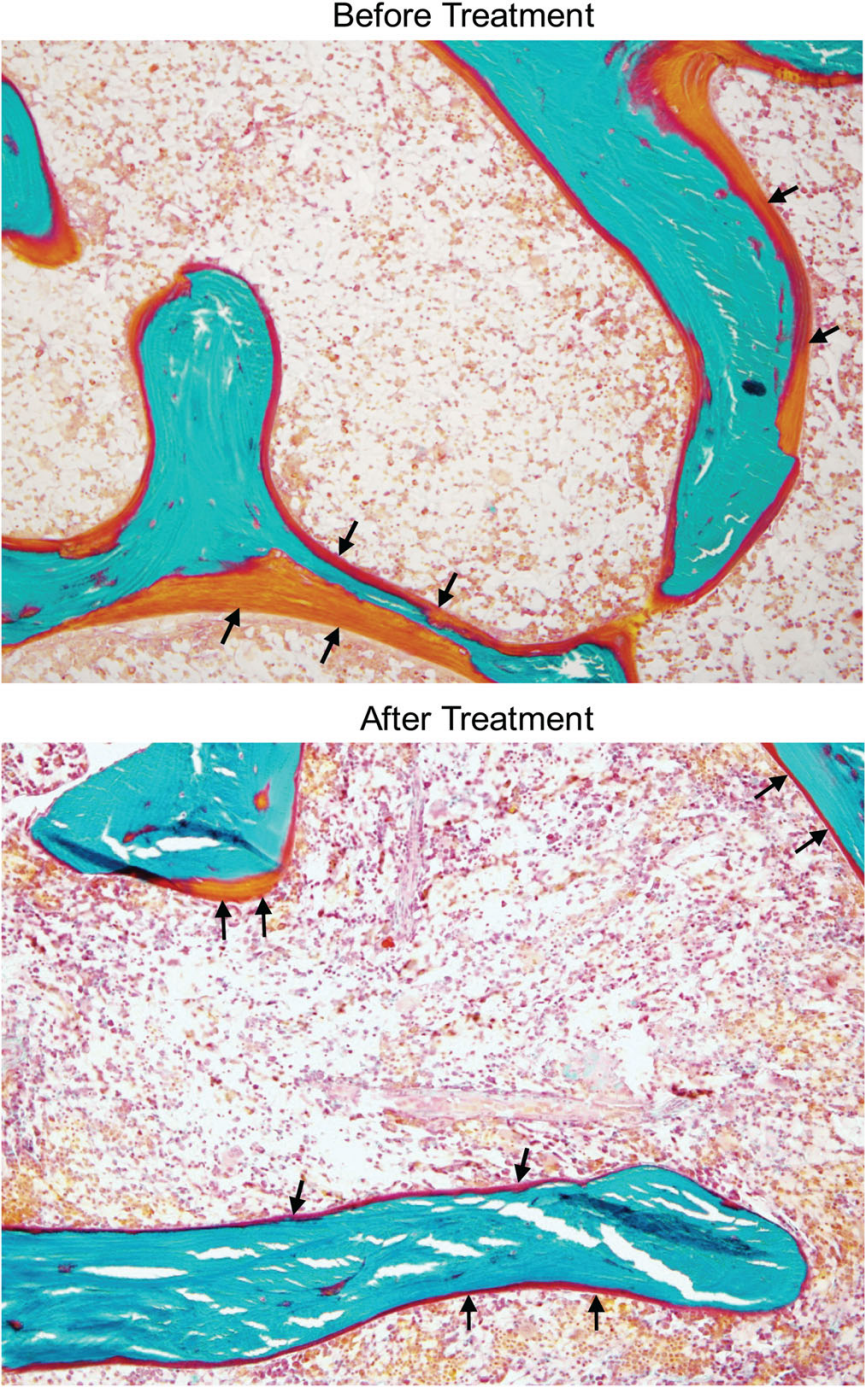
Undecalcified Goldner‐stained iliac bone samples in a 24‐year‐old female before and after treatment with burosumab. Mineralized bone is shown in green, unmineralized osteoid is shown in orange or red. After treatment, the layer of unmineralized osteoid (arrows) is thinner and covers a smaller percentage of mineralized bone surface. Magnification ×100.

After 48 weeks of treatment, all osteomalacia‐related histomorphometric measures improved significantly (Fig. 1). Osteoid volume/bone volume, the primary endpoint, decreased from a mean ± SD of 26.1% ± 12.4% at baseline to 11.9% ±6.6% at week 48, a mean absolute change of −15% (11%) and a mean ± SD (95% CI) percent change of −54% ± 20% (−69 to −40; *p* < 0.0001). Osteoid volume/bone volume decreased in all 10 subjects with evaluable samples; the percentage change ranged from −31% to −84%. Osteoid thickness changed by a mean ± SD of −32% ± 12% (95% CI, −40 to −24; *p* < 0.0001), from 17.2 ± 4.1 μm at baseline to 11.6 ± 3.1 μm at week 48. Osteoid surface/bone surface changed by a mean ± SD of −26% ± 15% (95% CI, −36 to −16; *p* = 0.0002), from 92% ± 3% at baseline to 68% ± 14% at week 48. Figure 2 illustrates the reduction in unmineralized osteoid following treatment with burosumab in a 24‐year‐old female. Using imputed values (see Subjects and Methods),^16^ mineralization lag time decreased from a median (min, max) of 1378.4 (129.6, 4909.1) days at baseline to 233.4 (69.8, 281.9) days at week 48, with a median (min, max) change of −83% (−96%, 54%) (95% CI, −95.1 to 51.8; *p* = 0.1094). Changes in other histomorphometric parameters included increases in mean cancellous bone volume/tissue volume, cortical width, trabecular thickness, mineralizing bone surface/bone surface, mineralizing surface/osteoid surface, and mineralization apposition rate. Indices of osteoclast activity, including osteoclast surface/bone surface and number of osteoclasts/bone perimeter, were not elevated at baseline and did not change appreciably with burosumab (Table 2).

**Table 2:** Additional Histomorphometric Parameters

	Current study UX023‐CL304		Healthy adult reference range^a^ mean ± SD; median (min, max)
Parameter	*n*	Mean ± SD; median (min, max)	
Bone volume/tissue volume (%)			27.8 ± 4.5; 27.4 (18.9, 34.7)
Baseline	9	31.1 ± 7.4; 30.1 (21.0, 44.4)	
Week 48	11	38.6 ± 12.8; 36.4 (23.3, 73.7)	
Cortical width (μm)			1010 ± 200; 1080 (630, 1300)
Baseline	10	1057 ± 294; 996 (515, 1530)	
Week 48	10	1150 ± 457; 1327 (468, 1676)	
Trabecular thickness (μm)			153 ± 24; 156 (111, 192)
Baseline	10	151 ± 53; 144 (94, 267)	
Week 48	11	166 ± 59; 195 (91, 245)	
Mineralizing surface/bone surface (%)			7.9 ± 2.7; 8.3 (3.3, 12.4)
Baseline	11	6.0 ± 4.8; 3.3 (1.0, 12.9)	
Week 48	10	7.0 ± 3.7; 5.8 (2.2, 13.6)	
Mineralizing surface/osteoid surface (%)			57.9 ± 13.8; 59.2 (39.9, 74.9)
Baseline	11	6.5 ± 5.1; 3.7 (1.1, 13.8)	
Week 48	10	10.8 ± 6.1; 8.1 (4.0, 20.3)	
Mineral apposition rate (μm/day)			0.75 ± 0.09; 0.77 (0.57, 0.86)
Baseline	11	0.58 ± 0.45; 0.43 (0.3, 1.8)	
Week 48	11	0.62 ± 0.19; 0.60 (0.3, 1.0)	
Osteoclast surface/bone surface (%)			1.0 ± 0.4; 0.9 (0.5, 1.9)
Baseline	8	0.4 ± 0.3; 0.3 (0.1, 1.0)	
Week 48	11	0.5 ± 0.4; 0.4 (0.1, 1.2)	
Number of osteoclasts/bone perimeter (1/mm)			0.3 ± 0.1; 0.3 (0.2, 0.6)
Baseline	8	0.1 ± 0.1; 0.1 (0.0, 0.3)	
Week 48	11	0.2 ± 0.1; 0.2 (0.0, 0.4)	

a Healthy reference range is based on data from 8 to 12 individuals between the ages of 17.0 and 22.9 years who underwent surgery for reasons independent of abnormalities in bone development and metabolism.^14^

Because of an AE, one subject missed burosumab doses at weeks 32 and 36 before resuming at week 40; to account for the lapse in dosing, this subject's posttreatment bone biopsy was performed at week 56 instead of week 48. Another subject was treated with tetracycline for sinusitis/bronchitis during the tetracycline labeling period before the week 48 biopsy, requiring the tetracycline label to be repeated and the biopsy to be performed at week 56 instead of week 48.

**Figure 3:**
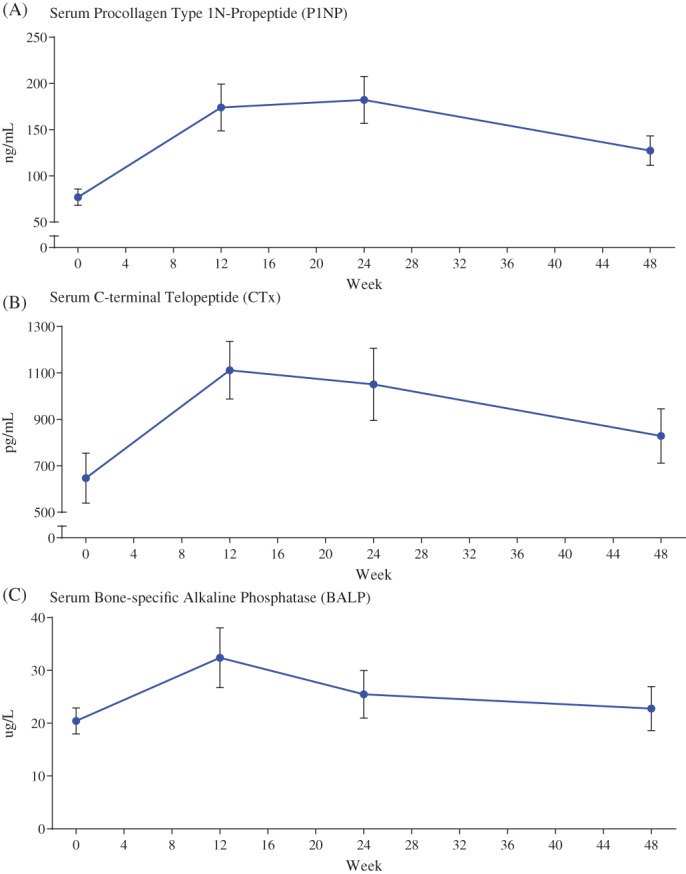
Biochemical markers of bone formation and resorption including (A) Serum Procollagen Type 1N‐Propetide, (B) Serum C‐terminal Telopeptode, and (C) Serum Bone‐specific Alkaline Phosphatase. Data is presented as mean ± standard error.

Markers of bone remodeling increased at week 48, with an LS mean increase of 77% (LS mean ± SE change 52.5 ± 11.6 ng/mL; *p* < 0.0001) in P1NP (Fig. 3A) and an LS mean increase of 36% (175.1 ± 44.0 pg/mL; *p* < 0.0001) in CTx (Fig. 3B). The highest measured increase in serum BALP occurred at week 12, with an LS mean increase of 53% (LS mean ± SE change 10.9 ± 3.5 μg/L; 95% CI, 4.0 to 17.9) at week 48 (Fig. 3C); the increase from baseline was 24% (4.5 ± 4.0 μg/L; *p* = 0.26).

Of the four active pseudofractures identified at baseline in four subjects, two had healed completely and two had partially healed by week 12; by week 48 three of the four active pseudofractures had healed, and the remaining pseudofracture was not evaluable because of a missing radiograph.

#### Pharmacodynamics

**Figure 4:**
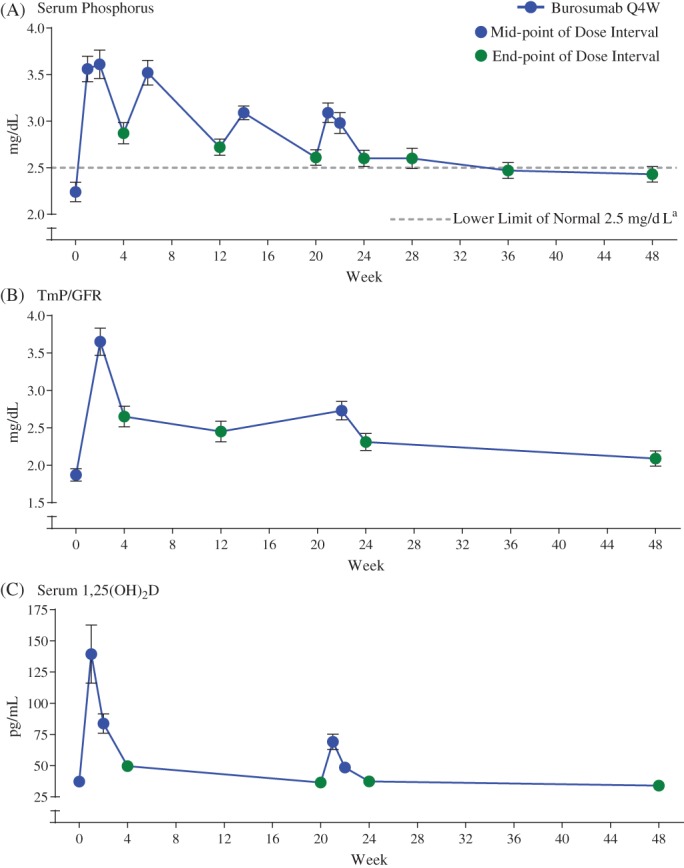
(A) Serum phosphorus, (B) TmP/GFR, and (C) serum 1,25(OH)_2_D. Data is presented as mean ± standard error. Assessments made at the mid‐point of the dose interval are shown in blue, and assessments made at the end‐point of the dose interval are shown in green. TmP/GFR = renal tubular maximum reabsorption of phosphate per glomerular filtration rate.

The mean serum phosphorus concentration, when averaged across the mid‐point of the dose intervals through week 24, was above the lower limit of normal (2.5 mg/dL) in 13 subjects (93%); the group mean ± SD serum phosphorus concentration during this period was 3.3 ± 0.4 mg/dL, a mean increase from baseline of 1.1 mg/dL or 50% (Fig. 4 *A*). Increases from baseline in serum phosphorus were maintained through week 48, when measured at the end of the dose cycle between weeks 24 and 48. Similarly, TmP/GFR increased at the first postbaseline assessment and remained above the baseline mean through week 48, with an LS mean ± SE increase from baseline to week 22 (measured at the mid‐point of the dose interval) of 0.9 ± 0.1 mg/dL or 48% (Fig. 4 *B*). The percentage increase from baseline to week 24 and week 48, at the end of the dose interval, were 26% and 14%, respectively. Mean serum 1,25(OH)_2_D concentrations were highest when measured 1 and 2 weeks after initiation of burosumab but declined to values near baseline at week 4; a similar pattern of mid‐dosing increases in serum levels of 1,25(OH)_2_D, was observed between weeks 20 and 24, although to a much lesser degree (Fig. 4 *C*).

#### Patient‐reported outcomes

With burosumab treatment, all three subscores from the Brief Pain Inventory decreased from baseline, indicating improvement. The Worst Pain score decreased from a mean ± SD of 6.6 ± 2.0 at baseline to 4.9 ± 2.6 at week 48, with an LS mean ± SE change of −1.9 ± 0.7 (95% CI, −3.2 to −0.6; *p* = 0.0054). Mean ± SD Pain Severity score decreased from 5.1 ± 1.8 at baseline to 3.4 ± 2.2 at week 48, with an LS mean ± SE change of −1.8 ± 0.6 (95% CI, −2.8 to −0.7; *p* = 0.0013). Mean ± SD Pain Interference score decreased from 5.2 ± 2.3 at baseline to 4.0 ± 2.7 at week 48, with an LS mean ± SE change of −1.5 ± 0.5 (95% CI, −2.5 to −0.4; *p* = 0.0060).

Burosumab treatment was also associated with decreases in the Brief Fatigue Inventory Assessment, indicating improvement. The mean ± SD Worst Fatigue score decreased from 6.8 ± 1.9 at baseline to 5.5 ± 2.5 at week 48, with an LS mean ± SE change of −1.6 ± 0.7 (95% CI, −2.8 to −0.3; *p* = 0.0156). The mean ± SD Global Fatigue score, calculated from averaging all nine items on the assessment, decreased from 5.0 ± 2.1 to 4.0 ± 2.4 at week 48, with an LS mean ± SE change of −1.2 ± 0.6 (95% CI, −2.3 to −0.1, *p* = 0.0359).

### Safety

Safety findings are reported for all subjects who completed at least 48 weeks of treatment, but no more than 84 weeks. All 14 subjects experienced at least one adverse event (AE) (Table 3). Ten (71%) subjects experienced an AE judged by the investigator to be related to burosumab, with the most common (ie, occurring in two or more subjects) being injection site urticaria (three subjects), abdominal pain (two subjects), asthenia (two subjects), injection site pain (two subjects), and injection site reaction (two subjects). Eleven subjects had 18 bone‐biopsy procedure‐related AEs that were all considered mild or moderate in severity by the investigator: 14 for pain, two for itch, and one each for headache and bandage irritation. Eleven subjects experienced mild to moderate procedure‐related pain; the majority of these events lasted less than a week. Two subjects experienced procedure‐related pain, considered mild, for an atypical duration of 102 and 93 days, respectively. There were no substantial complications from the bone biopsy procedure, such as excessive bleeding, infection, or severe pain.

**Table 3:** Safety Summary

Category	Incidence for all subjects (*n* = 14) *n* (%)
Any TEAE	14 (100.0)
Related TEAE	10 (71.4)
Injection site urticaria	3 (21.4)
Abdominal pain	2 (14.3)
Asthenia	2 (14.3)
Injection site pain	2 (14.3)
Injection site reaction	2 (14.3)
Serious TEAE	2 (14.3)
Paresthesia	1 (7.1)
Migraine	1 (7.1)
Related serious TEAE	0
Grade 3 or 4 TEAE	3 (21.4)
Paresthesia	1 (7.1)
Migraine and arthralgia	1 (7.1)
Uterine hemorrhage	1 (7.1)
TEAE leading to study discontinuation	0
TEAE leading to treatment discontinuation	0
TEAE leading to death	0

TEAE = treatment‐emergent adverse event.

Two subjects experienced serious AEs, a grade 3 paresthesia and grade 3 migraine headache; neither AE was considered related to burosumab, and both AEs resolved. Two additional grade 3 AEs occurred and were not considered related to burosumab: arthralgia in the same subject that experienced the serious AE migraine and a uterine hemorrhage in another subject. Of the prespecified AEs to monitor, five (36%) subjects had injection site reactions, eight (57%) subjects had hypersensitivity, and two (14%) subjects had restless leg syndrome. There were no deaths, no treatment‐emergent AEs (TEAEs) leading to discontinuation from the study or drug, and no incidents of hyperphosphatemia (defined as a serum phosphorus level >4.5 mg/dL). No subject experienced an increase in renal ultrasound scores between baseline and week 48. No noteworthy increases were observed in serum calcium or urine calcium excretion (Supplemental Fig. S2), or serum parathyroid hormone (Supplemental Fig. S3). Burosumab treatment did not result in clinically significant changes in any ECHO parameters, including left ventricular mass index and cardiac function, or ECG parameters, including QT interval and heart rate.

## Discussion

In adults with XLH, chronic hypophosphatemia leads to osteomalacia, fractures, pain, stiffness, decreased mobility, and an impaired quality of life. Burosumab, a monoclonal antibody against FGF23 approved for the treatment of XLH, was shown to reverse hypophosphatemia and improve clinical outcomes in both children and adults with XLH.^12^, ^13^, ^19^, ^20^, ^21^ Consistent with those published studies, the current study in adults with XLH demonstrates that 48 weeks of burosumab treatment restores phosphorus homeostasis. Additionally, this study demonstrates that improvement in phosphorus homeostasis is accompanied by significant improvement in the mineralization defect in XLH that manifests clinically as rickets in children and osteomalacia‐related fractures and bone pain in adults.

In the current study, all four key histomorphometric parameters of osteomalacia in adults with XLH were markedly elevated at baseline, indicating profound osteomalacia. Mean baseline osteoid volume/bone volume was 26.1%, a value similar to that reported by Sullivan and colleagues^6^ (19.1%) in a prior study of nine adults with XLH and substantially above the reference range reported in healthy young adults (0.41% to 3.05%) by Glorieux and colleagues^14^ using the same biopsy methodology. Mean baseline osteoid surface/bone surface was 91.73%, which is markedly higher than that observed in a healthy adults (4.9% to 23.9%).^14^ Baseline osteoid thickness (mean, 17.2 μm) and mineralization lag time (median, 1378.4 days) were also above mean values observed in healthy adults (5.4 to 8.9 μm^14^, ^22^ and 9.3 to 28.6 days,^14^, ^23^ respectively). In five subjects, the mineralization defect was so profound at baseline and uptake of tetracycline so low that imputation was needed to calculate mineralization lag time.

The key histomorphometric parameters in this study improved significantly in all subjects after 48 weeks of burosumab treatment, with a mean reduction of 54% in osteoid volume/bone volume, 26% in osteoid surface/bone surface, and 32% in osteoid thickness, respectively. The mean value of osteoid thickness decreased from 17.21 μm at baseline to 11.55 μm, a value that is within the range observed in healthy postmenopausal women (5.50 to 12.00 μm).^23^ The median mineralization lag time was 48 times that of the maximum value observed in 12 healthy adults ages 17 to 22 years, and was substantially reduced to just eight times the maximum normal value after 48 weeks of treatment.^14^ The decrease in mineralization lag time and reduction in the amount of unmineralized osteoid indicate substantial improvement in bone mineralization with burosumab treatment.

Treatment with multiple daily doses of oral phosphate and active vitamin D metabolites, referred to as conventional therapy, has led to improvement in rickets and growth in children with XLH, but there is less consensus regarding the benefits of conventional therapy in adults because of an unclear risk benefit ratio. Sullivan and colleagues^6^ reported that conventional therapy can improve, but not completely reverse, histomorphometric parameters of osteomalacia in a prospective study in adults with XLH. In that study, 16 adults with XLH were treated with conventional therapy and monitored for a mean duration of 4.2 years; nine of the 16 adults underwent paired iliac crest bone biopsies. The magnitude of the reduction in osteoid volume/bone volume seen with conventional therapy, 50%, was similar to that observed with burosumab, 54%. However, subjects in the present study received burosumab for only 48 weeks, whereas the mean duration of treatment in Sullivan and colleagues^6^ was more than four times longer.

Although conventional therapy may improve osteomalacia, it remains unclear whether the benefits of such therapy outweigh the potential risks. Sullivan and colleagues^6^ reported hypercalciuria and tertiary hyperparathyroidism. These events, as well as nephrocalcinosis, are known risks associated with conventional therapy.^2^, ^24^ In contrast, in the present study, we observed no changes in serum calcium or PTH concentrations, urine calcium excretion, nor renal nephrocalcinosis scores and no incidents of hyperphosphatemia. Burosumab's safety profile was similar to that seen in previous clinical trials, with most adverse events being mild to moderate in severity.^12^, ^13^, ^19^, ^20^, ^21^

Improvements in phosphorus metabolism, fracture healing, markers of bone remodeling, and patient‐reported outcomes in the present study were consistent with observations in a larger phase 3 placebo‐controlled study investigating the efficacy and safety of burosumab in adults with XLH (https://clinicaltrials.gov/ct2/show/NCT02526160).^12^, ^13^ In both studies, mean fasting serum phosphorus levels increased rapidly and all values determined at the mid‐point of the dose interval were above the lower limit of normal (2.5 mg/dL). Consistent with the high rate of fracture healing in the larger phase 3 study, two of the four active pseudofractures identified at baseline in the current study were completely healed after 12 weeks of burosumab treatment. In both studies, increases in serum P1NP (LS mean increase: current study 77%, previous phase 3 study 56%) and serum CTx (36%, 29%) also suggest that burosumab increases bone remodeling and formation.

Our study is not without limitations. Because of the invasive nature of a transiliac crest biopsy, a control arm was not included in the study design, and baseline values served as a reference for improvement. Nevertheless, the substantial changes in the key histomorphometric parameters indicate that burosumab provides meaningful improvement in measures of osteomalacia. Although the number of subjects studied was relatively small, all 10 subjects with evaluable biopsies showed improvement in the primary endpoint, osteoid volume/bone volume. Finally, it is possible that greater improvement in measures of osteomalacia would occur with a treatment duration longer than 48 weeks. This is not unreasonable because the sustained improvement in serum phosphate and 1,25‐dihydroxvitamin D levels, with the latter causing better intestinal calcium absorption, might well lead to further normalization of mineralization.

Our study focused on changes in iliac trabecular bone, the standard location for histomorphometric measures. However, osteomalacia in XLH also affects cortical bone, such as the shaft of the femur, tibia, and fibula, where most pseudofractures and fractures occur. Although we did not quantify the osteomalacia at these cortical sites, the significant healing of osteomalacia in the trabecular compartment, together with the dramatic healing of fractures and pseudofractures seen in the current study, as well as the previously published double‐blind randomized controlled trial support the conclusion that substantial healing of cortical osteomalacia also occurs with burosumab therapy.^12^, ^13^ It is also worth noting that the 33% increase in median cortical thickness in this study could indicate improvement in cortical strength.

With an increasing number of countries granting approval for burosumab in symptomatic adults with XLH, clinicians for the first time have therapeutic options for this patient population. No randomized controlled therapeutic trials have been conducted in adults but as noted, the prospective trial by Sullivan and colleagues^6^ showed 50% healing of osteomalacia after 4 years of conventional therapy in these patients. A subsequent cross‐sectional study reported amelioration in dental disease with long‐term administration of phosphorus and calcitriol to adults.^25^ Unlike the pivotal, double‐blind, randomized clinical trial with burosumab,^12^, ^13^ or the present report, changes in functional outcomes and fracture healing have not been systematically evaluated with conventional therapy, which limits a direct comparison of the two treatments. Conventional therapy requires meticulous patient adherence and frequent biochemical monitoring to avoid complications, which is part of the reason most adults are not treated for their disease. Further, conventional therapy does not prevent many of the long‐term complications of XLH in adults such as enthesopathy and hearing loss. It remains to be determined if burosumab will. There are no head‐to‐head trials comparing burosumab to conventional therapy in adults with XLH, but in children enrolled in a head‐to‐head trial, burosumab showed greater efficacy.^20^ The current work, as well the data from the recent randomized clinical trial,^12^, ^13^ provide healthcare professionals with a more complete picture of burosumab's efficacy in adults with XLH. Osteomalacia, a hallmark of XLH in adults, is associated with skeletal complications, pseudofractures, fractures, bone and joint pain, and an impaired quality of life. The present phase 3 study showed that burosumab significantly improves histomorphometric indices of osteomalacia, including osteoid volume/bone volume, osteoid thickness, osteoid surface/bone surface, and mineralization lag time. The improvements in osteomalacia coincided with increases in serum phosphorus and biochemical markers of bone remodeling. Such improvements in phosphorus homeostasis and healing of osteomalacia provide a physiologic basis for the efficacy of burosumab to heal fractures and pseudofractures in patients with XLH, and ameliorate symptoms such as pain and stiffness. Given its proven efficacy, better safety profile, and sound therapeutic rationale, burosumab represents an appealing therapeutic option for symptomatic adults with XLH.

## Disclosures

KI, PK, NI, TK, AN, and AAP served as principal investigators for this clinical study. FR received research support from Ultragenyx Pharmaceutical Inc. LZ and JSM are employees and shareholders of Ultragenyx Pharmaceutical Inc. MM was a previous employee of and is a shareholder of Ultragenyx Pharmaceutical Inc. AAP received payment from Ultragenyx for advisory board membership and lectures.

## Supporting information

Supplemental Figures

Protocol Amendment 3 29Aug2017_Redacted
