# Supplementary material for: Burosumab Improved Histomorphometric Measures of Osteomalacia in Adults with X‐Linked Hypophosphatemia: A Phase 3, Single‐Arm, International Trial
Source: J Bone Miner Res. 2019 Oct 1;34(12):2183–91. doi: 10.1002/jbmr.3843 (PMC6916280; doi:10.1002/jbmr.3843)
Supplement: Supplementary file 1 — Supplemental Figures [file JBMR-34-2183-s001.docx]

**Supplemental Materials**

## Supplemental Figure 1. UX023-CL304 CONSORT Diagram


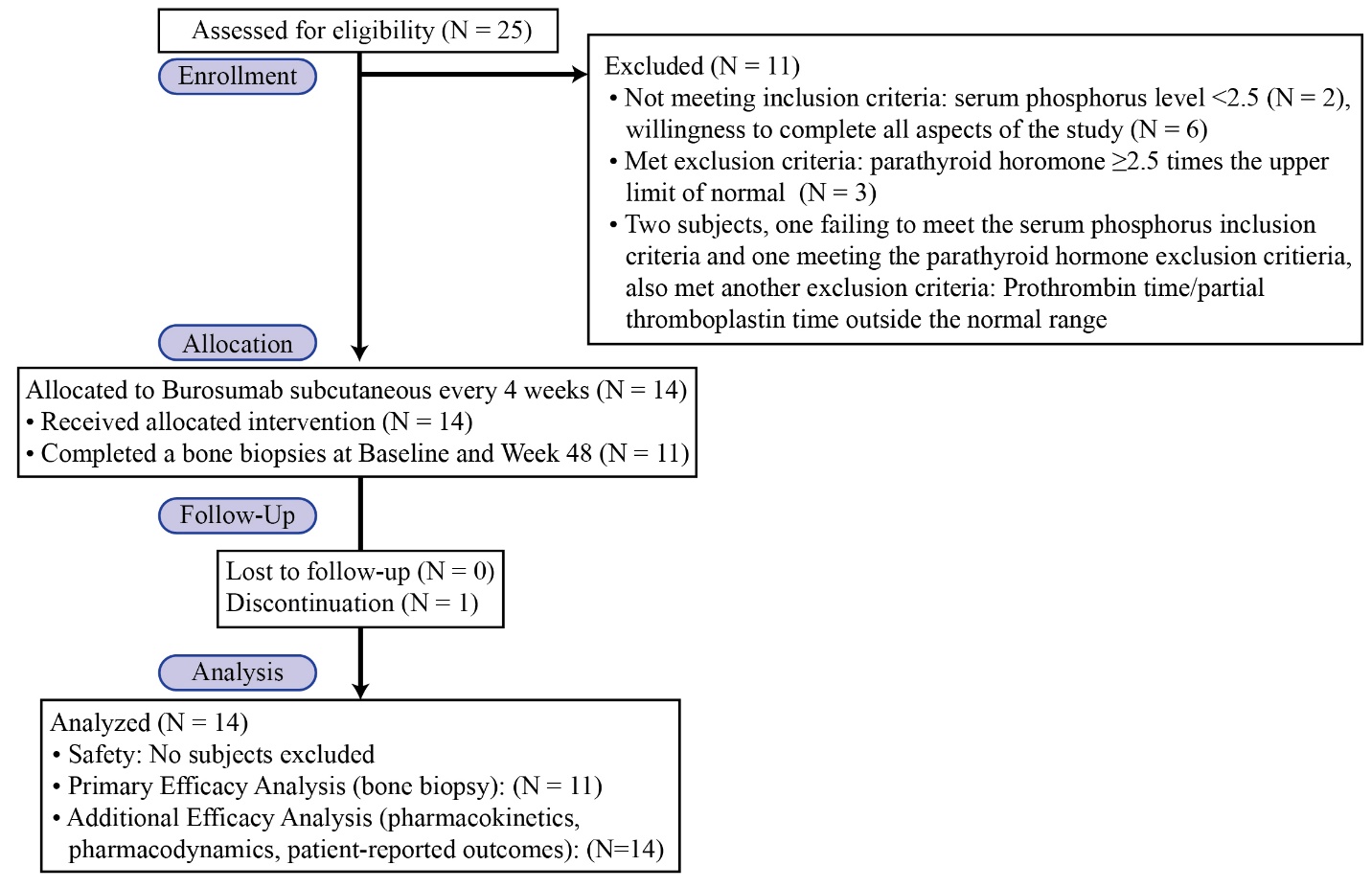


## Supplemental Figure 2. Serum and Urine Calcium


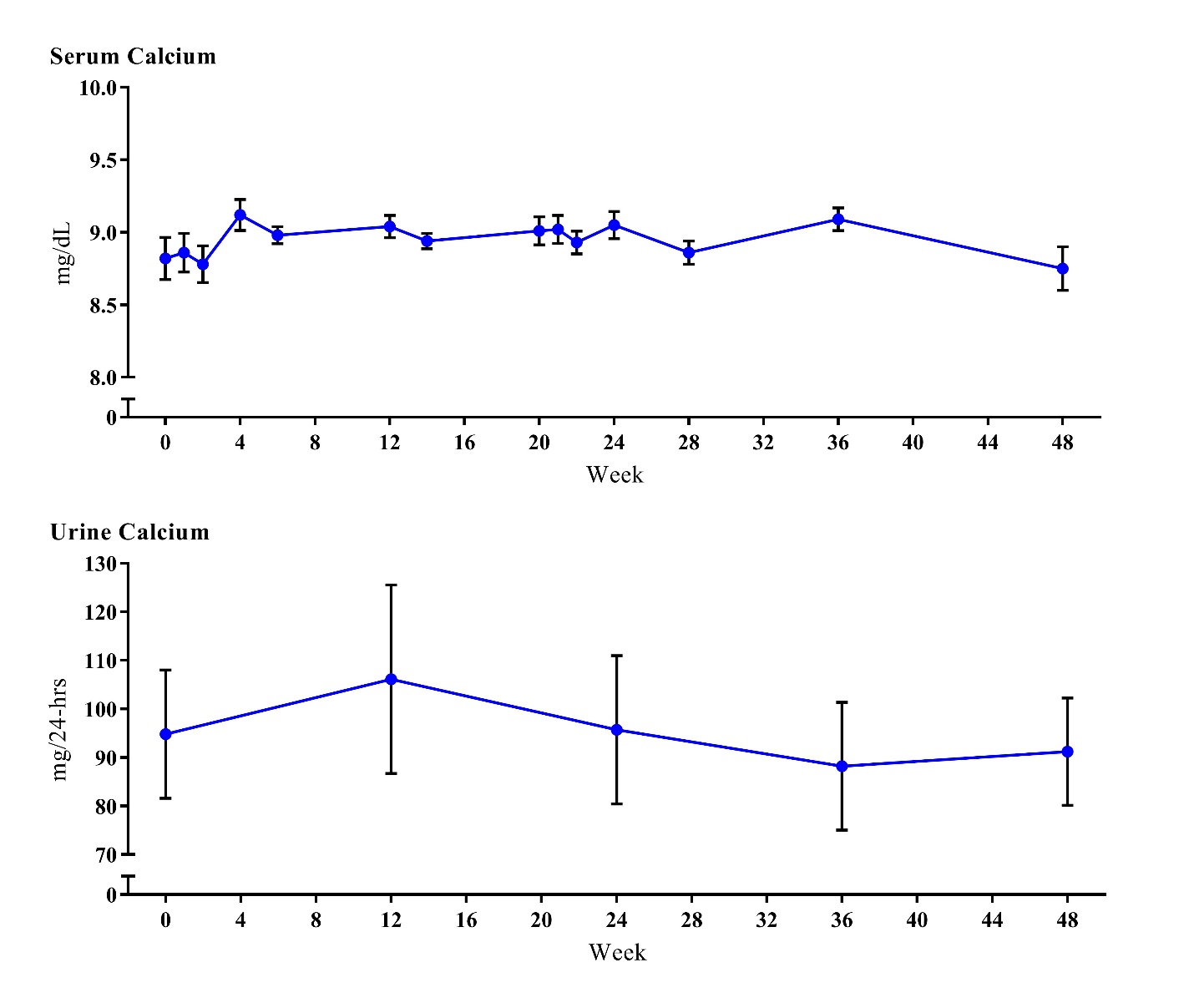


Data are expressed as mean ± standard error.

## Supplemental Figure 3. Serum Intact Parathyroid Hormone


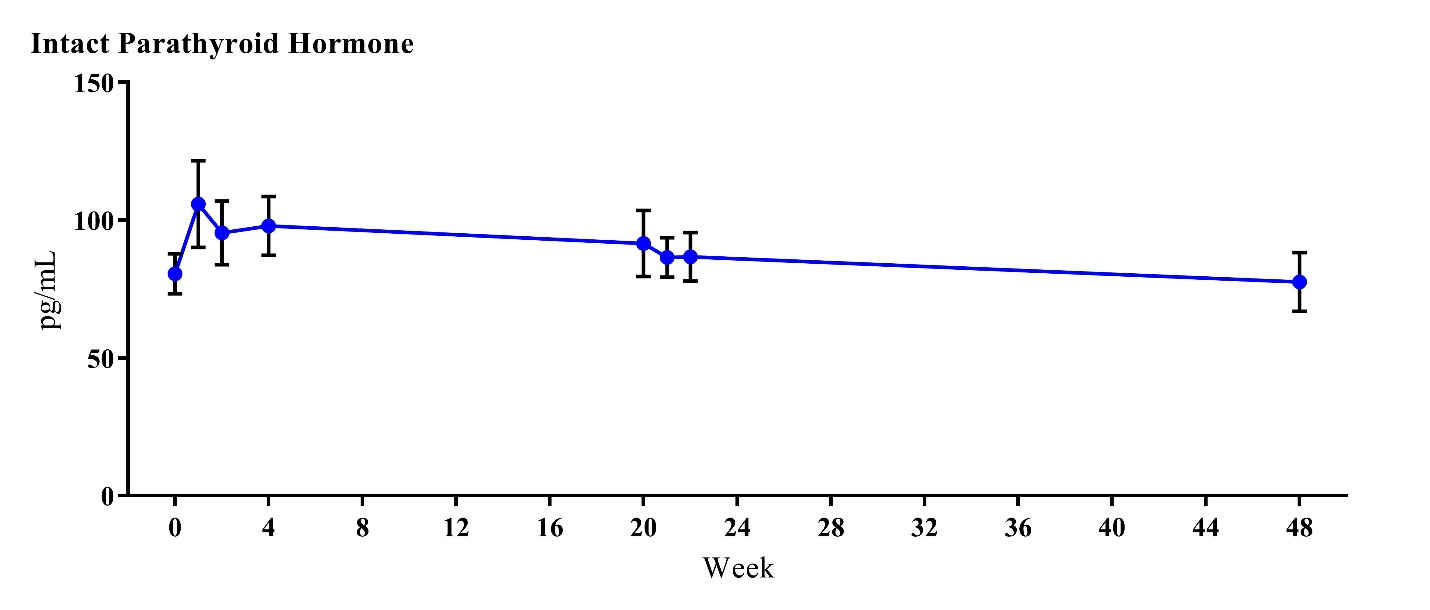


Data is presented as mean ± standard error.
