# Supplementary material for: Burosumab Improved Histomorphometric Measures of Osteomalacia in Adults with X‐Linked Hypophosphatemia: A Phase 3, Single‐Arm, International Trial
Source: J Bone Miner Res. 2019 Oct 1;34(12):2183–91. doi: 10.1002/jbmr.3843 (PMC6916280; doi:10.1002/jbmr.3843)
Supplement: Supplementary file 2 — Protocol Amendment 3 29Aug2017_Redacted [file JBMR-34-2183-s002.pdf]

An Open-Label, Single-Arm, Phase 3 Study to Evaluate the Effects of KRN23 on Osteomalacia in Adults with X-linked Hypophosphatemia (XLH)

**Protocol Number:**	**UX023-CL304**
**Original Protocol:**	**19 June 2015**
**Amendment 1:**	**29 March 2016**
**Amendment 2:**	**07 October 2016**
**Amendment 3:**	**29 August 2017**

**Investigational Product:**	KRN23 (Recombinant human IgG_1 monoclonal antibody to fibroblast growth factor 23 [FGF23])
**Indication:**	X-linked Hypophosphatemia (XLH)
**IND Number:**	76,488
**EudraCT Number:**	2015-001775-41
**Sponsor:**	Ultragenyx Pharmaceutical Inc.<br>60 Leveroni Court<br>Novato, CA, USA 94949
**Sponsor's Responsible Medical Officer:**	Javier San Martin, MD<br>Vice President, Clinical Sciences<br>Ultragenyx Pharmaceutical Inc.<br>[redacted]

This study is to be performed in compliance with the protocol, Good Clinical Practices (GCP), and applicable regulatory requirements.

Confidentiality Statement: This document and the information it contains is confidential and proprietary and is the property of Ultragenyx Pharmaceutical Inc. (Ultragenyx). Unauthorized disclosure, reproduction, transmission, or use of this document or information is strictly prohibited. By accepting this document, the recipient agrees that the information it contains will not be disclosed without written authorization from Ultragenyx, except to the extent required to satisfy Institutional Review Board /Ethics Committee procedures, to obtain written informed consent from potential subjects, or as required by law.

CLINICAL STUDY PROTOCOL AMENDMENT

SUMMARY OF CHANGES AND RATIONALE

UX023-CL304 Amendment 3

29 August 2017

Protocol UX023-CL304 Amendment 2 (dated 07 October 2016) has been modified by Amendment 3 to extend treatment duration for study subjects in the United States (US) and to describe the data monitoring through the Treatment Extension Periods. Minor edits, clarifications, and typographical corrections have also been made. The major protocol changes that impact study design and conduct are summarized below:

1. **Study Design and Duration:** In Sections 7.1 and 7.4.3.1 and other relevant sections, a second Treatment Extension Period II that includes an additional up to approximately 45 weeks of KRN23 treatment, until end of September 2018, has been added for subjects enrolled at sites in the US only. (The duration of this period will vary for individual subjects and will be determined by the time from start of Week 97 through final visit schedule 30 September 2018.) In addition, in these sections and Section 8.5.4.1 and other relevant sections, Safety Follow-up telephone calls (TCs) over an interval of up to 8 weeks following the End of Study or Early Termination Visit have been added for subjects not immediately continuing KRN23 treatment under commercial use or another mechanism upon completion of study drug treatment or early withdrawal from this study. The maximum study duration has consequently been changed to up to approximately 149 weeks. For subjects outside of the US, the duration of study treatment will remain 96 weeks, followed by Safety Follow-up TCs over an interval of up to 8 weeks following the End of Study or Early Termination Visit if not continuing KRN23 treatment under commercial use or another mechanism upon completion of study drug or early withdrawal from this study. The end of study is defined as the last day that protocol-specified assessments (including telephone contact) are conducted for the last subjects in the study.

   Rationale: Treatment Extension Period II has been added for subjects at US sites to provide KRN23 treatment to these subjects until a time when KRN23 is expected to be commercially available in the US. Safety and Efficacy parameters will continue to be assessed while the subjects remain on study drug. Subjects enrolled outside the US may have access to continued KRN23 treatment after completion of Extension Period I through various regional mechanisms, in accordance with local laws and regulations. Safety Follow-up TCs have been added after the extension periods to collect information as appropriate on ongoing or new adverse events, serious adverse events, and concomitant medications after subjects discontinue study drug but before KRN23 treatment under commercial use or another mechanism begins.

2. **Anti-KRN23 Antibodies:** In Section 7.5.3.8.2 and the Schedule of Events, the term HAHA (human anti-human antibody) in reference to anti-KRN23 antibody testing, has been replaced with the term ADA (anti-drug antibody).

   Rationale: This change is a clarification. The immunogenicity of KRN23 is evaluated by quantifying total anti-drug antibodies (ADA), independent of isotype, in human serum. While the study protocol previously used the term "HAHA" for this assessment, it has been replaced with the more correct and specific term, ADA.

3. **Safety Monitoring:** Section 7.6.7 and Section 8.5.4.6 have been updated to describe safety monitoring during the Treatment Extension Periods I and II of the study.

   Rationale: In this open-label study, the DMC will continue to monitor safety through Week 48 as planned. During the Treatment Extension Periods I and II, safety will be monitored on an ongoing basis by the Study Safety Review Team (SSRT), an internal safety review team defined and in place since the original protocol.

4. **Exploratory Efficacy Endpoints:** In Section 7.6.4.4, the exploratory endpoint of healing of pre-existing pseudofractures and/or Looser zones, as defined by skeletal survey at baseline and subsequent targeted radiography has been updated to clarify that it comprises the following components: the number of active pseudofractures and/or fractures as defined by skeletal survey at baseline and the numbers and percentages of the baseline active pseudofractures/fractures that were healed, partially healed, unchanged, and worsened at post-baseline visits, and the number of subjects with baseline active pseudofractures and/or fractures at baseline and the numbers of those subjects who had changes from baseline to healed, partially healed, unchanged, and worsened at post-baseline visits. Also, the descriptions of other exploratory endpoints were updated for clarity.

   Rationale: The section was updated to reflect the exploratory efficacy endpoints planned for analysis in this study.

5. **Physical Examination**: Section 7.5.3.4 was modified to specify that the genitourinary component of the physical exam will be performed as age-appropriate at the Investigator's discretion.

   Rationale: this modification was added to clarify that the genitourinary scope should be non-invasive and as per age-appropriate standard of care, at the Investigator's discretion based on the clinical judgement and/or safety need.

2 SYNOPSIS

TITLE OF STUDY:

An Open-Label, Single-Arm, Phase 3 Study to Evaluate the Effects of KRN23 on Osteomalacia in Adults with X-linked Hypophosphatemia (XLH)

PROTOCOL NUMBER:

UX023-CL304

STUDY SITES:

Approximately 14 sites globally

PHASE OF DEVELOPMENT:

Phase 3

RATIONALE:

XLH is a disorder of hypophosphatemia, renal phosphate wasting, and defective bone mineralization caused by inactivating mutations in the *PHEX* gene (phosphate-regulating gene with homologies to endopeptidases on the X chromosome). In the absence of functional PHEX, release of fibroblast growth factor 23 (FGF23) by osteocytes is greatly increased. Excess circulating FGF23 signals the kidney tubules to decrease phosphate reabsorption by down-regulating NaPi-IIa and NaPi-IIc in the tubular cells and suppressing 1,25-dihydroxyvitamin D (1,25(OH)$_2$D) production, resulting in decreased intestinal absorption of calcium and phosphate producing hypophosphatemia. In adults with XLH, a hallmark of the disease is osteomalacia, a defect in bone mineralization due to chronically low serum phosphorus. Osteomalacia is characterized by a lack of proper mineralization, a prolonged mineralization process, and an accumulation of osteoid tissue. It is also associated with a reduction in bone remodeling and reduced bone quality and is a key contributor to the symptoms of bone and joint pain and the non-traumatic pseudofractures and reduced quality of life experienced by adults with XLH.

KRN23 is a recombinant fully human IgG$_1$ monoclonal antibody being developed to treat XLH by binding to FGF23 and inhibiting its activity, thereby increasing serum phosphorus levels and improving osteomalacia and related symptoms. Histologic and histomorphometric analyses of iliac crest bone biopsies are the gold standard for evaluating changes in osteomalacia, thus it is important to characterize changes in bone biopsy parameters in response to KRN23 treatment. Bone biopsy studies in adults with XLH receiving oral phosphate and vitamin D therapy have shown incomplete healing of osteomalacia. In addition, there are no systematic studies in adults to show this regimen improves symptoms or outcomes, and there are concerns about the risk of long-term complications, particularly nephrocalcinosis, which increases with dose and duration of therapy. More efficacious, safer, and convenient therapies clearly are needed for adults who continue to experience XLH-related symptoms and complications.

A Phase 1 study established the pharmacokinetic (PK) profile of KRN23. A Phase 1/2 study and associated extension study evaluated the pharmacodynamics (PD) of KRN23 on phosphate metabolism and related measures of the phosphate-calcium mineral control system. The safety data from these studies have shown that KRN23 in single and repeated monthly doses up to 1.0 mg/kg

was well tolerated by adult XLH subjects. KRN23 sufficiently increased serum phosphorus levels such that improvements in bone physiology, structure, and function would be expected.

A randomized, double-blind, placebo-controlled Phase 3 study is being conducted to confirm the efficacy and safety of KRN23 treatment, and establish the beneficial impact on patient-reported and functional outcomes in adults with XLH. The current open-label study will examine the efficacy of KRN23 on bone quality, including the effects on osteomalacia at the tissue level, and provide an additional assessment of safety. Long term safety and efficacy data will be collected during the Treatment Extension Periods I and II.

PRIMARY EFFICACY HYPOTHESIS:

Treatment with 1.0 mg/kg KRN23 monthly will improve XLH-associated osteomalacia as determined by osteoid volume (osteoid volume/bone volume, OV/BV).

OBJECTIVES:

Primary Efficacy Objective:

Establish the effect of KRN23 treatment on improvement in XLH-associated osteomalacia as determined by osteoid volume (osteoid volume/bone volume, OV/BV).

Secondary Efficacy Objectives:

The key secondary efficacy objective is to establish the effect of KRN23 treatment on increasing serum phosphorus levels in adults with XLH

Other secondary efficacy objectives are to establish the effect of KRN23 treatment in adults with XLH on:

- Changes from baseline in additional histomorphometric parameters – including osteoid thickness (O.Th), osteoid surface/bone surface (OS/BS), and mineralization lag time (MLt)
- Changes from baseline in parameters of bone mineralization including mineral apposition rate (MAR), mineralizing surface (MS/BS), bone formation rate (BFR), and others
- Additional PD markers reflecting the status of phosphorus homeostasis and renal function
- Bone remodeling as assessed by bone turnover markers

Exploratory Efficacy Objectives:

- Examine the effect of KRN23 treatment in adults with XLH on pseudofracture/fracture healing
- Patient reported outcomes (PROs) assessing skeletal pain and fatigue

Pharmacokinetic Objective:

Assess the PK of KRN23 throughout the dosing cycle following the first doses and steady state

Safety Objective:

Establish the safety and tolerability profile of KRN23 in the treatment of adults with XLH including adverse events (AEs), ectopic mineralization risk, cardiovascular effects, and immunogenicity profile

STUDY DESIGN AND METHODOLOGY:

UX023-CL304 is a Phase 3 open-label, single-arm, multicenter study to establish the effects of KRN23 on bone quality and osteomalacia associated with XLH. Approximately 14 adult subjects with a diagnosis of XLH supported by typical clinical and biochemical features and who have not received oral phosphate and vitamin D therapy in the past two years will be enrolled. At least 3 subjects of each sex will be enrolled. Iliac crest bone biopsies will be performed at baseline and 48 weeks. Baseline histologic and histomorphometric assessments of the bone biopsy specimens will be performed as each biopsy is completed to assess sample quality and confirm the presence of osteomalacia in at least 8 subjects. If a subject is determined not to have osteomalacia at the time of the initial biopsy, that subject will continue on study but will not undergo the second bone biopsy procedure at Week 48. All other assessments will be completed as scheduled. The goal of the study is to assess changes in bone quality; histologic and histomorphometric evaluation of iliac crest bone biopsies will be supported by changes in serum phosphorus and biochemical markers of bone turnover and additional PD markers associated with FGF23-mediated processes. Pseudofractures and PROs will provide additional information on KRN23 efficacy. Safety, immunogenicity, and PK of KRN23 will also be evaluated.

KRN23 will be administered via subcutaneous (SC) injections monthly (Q4W, 28 days) for 48 weeks. Subjects who complete the 48 weeks of the Open-Label Treatment Period will then continue into an additional 48-week Treatment Extension Period I (until Week 96). For subjects at study sites outside of the United States (US), the Week 96 Visit will be their End of Study (EOS) efficacy visit (referred to as EOS I). For subjects at sites in the US, treatment will continue for up to an additional 45 weeks in an open-label Treatment Extension Period II, until the end of September 2018, at which time they will have their EOS efficacy visit (referred to as EOS II). (The duration of this period will vary for individual subjects and will be determined by the time from the start of Week 97 through their EOS II Visit). For subjects not immediately continuing KRN23 treatment under commercial use or another mechanism upon completion of study drug treatment or early withdrawal from this study, a Safety Follow-up telephone call (TC) will occur 4 weeks (+5 days) after the subject’s final study site visit (ie EOS I [non-US subjects], EOS II [US subjects], or Early Termination [ET] visit) to collect information on any ongoing or new AEs, serious adverse events (SAEs), and concomitant medications. If the subject is not continuing KRN23 therapy under commercial use or another mechanism at that time, an additional Safety Follow-up TC will occur at 8 weeks (+ 5 days) after the subject’s final study site visit. The end of this study is defined as the last day that the protocol specified assessments (including telephone contact) are conducted for the last subject.

NUMBER OF SUBJECTS PLANNED:

Approximately 14 adult subjects are expected to be enrolled in the study including at least 8 subjects with a confirmed diagnosis of osteomalacia

DIAGNOSIS AND CRITERIA FOR INCLUSION AND EXCLUSION:

Individuals eligible to participate in this study must meet all of the following criteria:

1) Male or female, aged 18 - 65 years, inclusive
2) Diagnosis of XLH supported by classic clinical features of adult XLH (such as short stature or bowed legs), and at least ONE of the following at Screening:
    - Documented *PHEX* mutation in either the patient or in a directly related family member with appropriate X-linked inheritance
    - Serum iFGF23 level > 30 pg/mL by Kainos assay
3) Biochemical findings consistent with XLH based on overnight fasting (min. 8 hours):
    - Serum phosphorus < 2.5 mg/dL at Screening
    - TmP/GFR < 2.5 mg/dL at Screening
4) Presence of skeletal pain attributed to XLH/osteomalacia, as defined by a score of ≥ 4 on the Brief Pain Inventory question 3 (BPI-Q3, Worst Pain) at Screening. (Skeletal pain that, in the opinion of the investigator, is attributed solely to causes other than XLH/osteomalacia—for example, back pain or joint pain in the presence of severe osteoarthritis by radiograph in that anatomical location—in the absence of any skeletal pain likely attributed to XLH/osteomalacia should not be considered for eligibility)
5) Estimated glomerular filtration rate (eGFR) ≥ 60 mL/min (using the Chronic Kidney Disease Epidemiology Collaboration [CKD-EPI] equation) or eGFR of 45 to <60 mL/min at Screening with confirmation that the renal insufficiency is not due to nephrocalcinosis
6) Provide written informed consent after the nature of the study has been explained, and prior to any research-related procedures. If the subject is a minor, provide written assent and have a legally authorized representative willing and able to provide written informed consent
7) Willing to provide access to prior medical records for the collection of biochemical and radiographic data and disease history
8) Females of child-bearing potential must have a negative urine pregnancy test at Screening and Baseline and be willing to have additional pregnancy tests during the study. Females considered not to be of childbearing potential include those who have not experienced menarche, are post-menopausal (defined as having no menses for at least 12 months without an alternative medical cause) or are permanently sterile due to total hysterectomy, bilateral salpingectomy, or bilateral oophorectomy.
9) Participants of child-bearing potential or fertile males with partners of child-bearing potential who are sexually active must consent to use a highly effective method of contraception as determined by the site Investigator from the period following the signing of the informed consent through the final Safety Follow-up TC (as defined in Section 7.4.3.1)
10) Must, in the opinion of the investigator, be willing and able to complete all aspects of the study, adhere to the study visit schedule and comply with the assessments

Individuals who meet any of the following exclusion criteria will not be eligible to participate in the study:

1) Use of any pharmacologic vitamin D metabolite or analog (e.g. calcitriol, doxercalciferol, and paricalcitol) within the 2 years before Screening
2) Use of oral phosphate within 2 years before Screening
3) Use of aluminum hydroxide antacids, acetazolamides, and thiazides within 7 days prior to Screening
4) Use of bisphosphonates in the 2 years prior to Screening
5) Use of denosumab in the 6 months prior to Screening
6) Use of teriparatide in the 2 months prior to Screening
7) Chronic use of systemic corticosteroids defined as more than 10 days in the 2 months prior to Screening
8) Corrected serum calcium level ≥ 10.8 mg/dL (2.7 mmol/L) at Screening
9) Serum iPTH ≥ 2.5 times the upper limit of normal (ULN) at Screening
10) Use of medication to suppress PTH (cinacalcet for example) within 60 days prior to Screening
11) Prothrombin time/Partial thromboplastin time (PT/PTT) outside the normal range at Screening
12) Evidence of any disease or use of anticoagulant medication (such as warfarin, heparin, direct thrombin inhibitors, or xabans that, in the opinion of the investigator, cannot be discontinued) that may increase the risk of bleeding during the biopsy procedure
13) Pregnant or breastfeeding at Screening or planning to become pregnant (self or partner) at any time during the study
14) Unable or unwilling to withhold prohibited medications throughout the study
15) Documented dependence on narcotics
16) Use of KRN23, or any other therapeutic monoclonal antibody within 90 days prior to Screening
17) Use of investigational product or investigational medical device within 30 days prior to Screening, or requirement for any investigational agent prior to completion of all scheduled study assessments.

    OR, in Japan, use of any investigational product or investigational medical device within 4 months prior to Screening, or requirement for any investigational agent prior to completion of all scheduled study assessments.

18) Presence or history of any hypersensitivity, allergic or anaphylactic reactions to any monoclonal antibody or KRN23 excipients that, in the judgment of the investigator, places the subject at increased risk for adverse effects
19) History of allergic reaction or adverse reactions to tetracycline or demeclocycline

20) Prior history of positive test for human immunodeficiency virus antibody, hepatitis B surface antigen, and/or hepatitis C antibody
21) History of recurrent infection (other than dental abscesses, which are known to be associated with XLH) or predisposition to infection, or of known immunodeficiency
22) Presence of malignant neoplasm (except basal cell carcinoma)
23) Presence of a concurrent disease or condition that would interfere with study participation or affect safety
24) Presence or history of any condition that, in the view of the investigator, places the subject at high risk of poor treatment compliance or of not completing the study

INVESTIGATIONAL PRODUCT, DOSE, AND MODE OF ADMINISTRATION

KRN23 is a sterile, clear, colorless, and preservative-free solution supplied in single-use 5-mL vials containing 1 mL of KRN23 at a concentration of 30 mg/mL. KRN23 will be administered without dilution. All subjects will receive 1.0 mg/kg KRN23 monthly (Q4W, 28 days) rounded to the nearest 10 mg. The amount of drug administered will be calculated based on baseline weight and a 1.0 mg/kg KRN23 dose level (rounded to the nearest 10 mg) up to a maximum dose of 90 mg. The dose will remain fixed for the duration of the study, provided serum phosphorous levels do not exceed 5.0 mg/dL (1.61 mmol/L) at any time or 4.5 mg/dL (1.45 mmol/L) on two occasions. The dose will be recalculated if body weight changes by more than 20% from the baseline measurement.

Subjects will receive study drug via SC injection to the abdomen, upper arms, or thighs; the injection site will be rotated with each injection. No more than 1.5 mL may be administered to a single injection site. If the dose requires more than 1.5 mL, multiple injections must be administered, each at a different injection site.

If serum phosphorus increases above 5.0 mg/dL (1.61 mmol/L) at any time the actual dose will be decreased by half. If serum phosphorous increases above the ULN (4.5 mg/dL; 1.45 mmol/L) but does not exceed 5.0 mg/dL (1.61 mmol/L), the dose will be adjusted only if a second serum phosphorus result exceeds the ULN. Following a downward dose adjustment, the investigator together with the medical monitor should determine how and when to dose titrate up. Unscheduled serum phosphorus assessments may be necessary. Based on the totality of the data from studies INT-001 and INT-002 over a period of 16 months in which most subjects were treated with 1.0 mg/kg KRN23 and no subject experienced an elevation of serum phosphorus that approached the 4.5 mg/dL (1.45 mmol/L) threshold, it is considered unlikely that dose adjustment will be necessary.

REFERENCE THERAPY, DOSE, AND MODE OF ADMINISTRATION:

The study is a single-arm, open-label study. All subjects will be on active treatment; no reference therapy or placebo will be administered.

DURATION OF TREATMENT:

The planned duration of treatment in this study is up to 96 weeks for subjects at sites outside of the US. For subjects at sites in the US, the planned duration of treatment is up to approximately 141 weeks, until end of September 2018. All Subjects will receive treatment with KRN23 for 48 weeks as part of the Open-Label Treatment Period. Subjects will then continue into an additional 48-week Treatment Extension Period I and, if at a US site, up to approximately 45 weeks, until end of September 2018, in Treatment Extension Period II. The duration of Treatment Extension Period II will vary for individual subjects and will be determined by the time from the start of Week 97 through their end of study visit.

CRITERIA FOR EVALUATION:

Primary Efficacy Endpoint:

The primary endpoint is the percent change from baseline in osteoid volume/bone volume (OV/BV) at Week 48 based on analysis of iliac crest bone biopsies

Key Secondary Efficacy Endpoint:

The key secondary efficacy endpoint is the proportion of subjects achieving mean serum phosphorus levels above the lower limit of normal (LLN; 2.5 mg/dL [0.81 mmol/L]) at the mid-point of the dose interval (i.e., Weeks 2, 6, 14, and 22), as averaged across dose cycles between baseline and Week 24.

Secondary Efficacy Endpoints:

- Percent changes from baseline in additional histomorphometric parameters – including osteoid thickness (O.Th), osteoid surface/bone surface (OS/BS), and mineralization lag time (MLt)
- Changes from baseline in MAR, MS/BS, BFR and additional measures of bone formation and remodeling
- Additional measures to assess serum phosphorus levels between baseline and Week 24 include:
    - Proportion of subjects achieving mean serum phosphorus levels above the LLN (2.5 mg/dL [0.81 mmol/L]) at the end of the dosing cycle (4 weeks after dosing), as averaged across dose cycles
    - Mid-point of dosing cycle: mean change from baseline and percent change from baseline averaged across dose cycles
    - End of dosing cycle: mean change from baseline, and percent change from baseline averaged across dose cycles
    - Cumulative exposure: area under the curve (AUC)

- Change from baseline over time in serum 1,25$(OH)_2$D, urinary phosphorus, ratio of renal tubular maximum reabsorption rate of phosphate to glomerular filtration rate (TmP/GFR), and tubular reabsorption of phosphate (TRP)

- Change and percent change from baseline over time in serum biochemical markers of bone turnover, including procollagen type 1 N-propeptide (P1NP), carboxy-terminal cross-linked telopeptide of type I collagen (CTx), and bone-specific alkaline phosphatase (BALP)

Exploratory Efficacy Endpoints:

- Healing of active pseudofractures and/or fractures, as defined by skeletal survey at baseline and subsequent targeted radiography
- Change from baseline in BPI Pain Severity and in Pain Interference scores over time
- Change from baseline over time in Brief Fatigue Inventory (BFI) Q3-Worst Fatigue Scores
- Change from baseline over time in BFI Global Fatigue Score, calculated by averaging all 9 items on the BFI

Drug Concentration Measurements:

PK samples will be obtained from all subjects throughout the study at time points representing the peak and trough exposure levels. .

Safety Assessments:

Safety will be evaluated by the incidence, frequency and severity of AEs and SAEs, including clinically significant changes from baseline to scheduled time points in the following safety variables:

<u>General Safety Variables</u>:

- Vital signs and weight
- Physical examinations
- eGFR
- Chemistry, hematology, and urinalysis, including additional KRN23/XLH biochemical parameters of interest (creatinine, calcium, and intact parathyroid hormone (iPTH)
- Immunogenicity (anti-drug antibody [ADA] against KRN23)
- Concomitant medications

<u>Ectopic Mineralization Safety Assessments</u>:

- Renal ultrasound

- Echocardiogram (ECHO) and electrocardiogram (ECG)

STATISTICAL METHODS:

A full description of the statistical evaluations will be provided in the Statistical Analysis Plan (SAP).

Sample Size:

The study will enroll approximately 14 adult subjects with XLH including at least 8 subjects with a confirmed diagnosis of osteomalacia. To ensure a level of gender balance, at least 3 subjects of each sex will be enrolled. At least 6 paired biopsy specimens are expected at the end of the study. A reduction in excess osteoid is expected to be shown in all subjects with paired biopsies with an estimated ≥ 50% reduction from baseline in osteoid thickness. The sample size and study duration are believed to be sufficient to enable characterization of KRN23 effects on bone tissue and skeletal health.

Analysis Populations:

Primary Analysis Set: The primary analysis set will include enrolled subjects with baseline and follow-up (Week 48 or ET) bone biopsy data.

Full Analysis Set: The full analysis set for efficacy is defined as all enrolled subjects who receive at least one dose of study drug.

Safety Analysis Set: The safety analysis set consists of all enrolled subjects who receive at least one dose of study drug. This analysis set will be used for the analyses of all safety endpoints.

Treatment Extension Analysis Set: The treatment extension analysis set consists of all enrolled subjects who continued after open-label treatment period and received at least one dose during the Treatment Extension Period I. This analysis set will be used for the analyses of efficacy and safety endpoints.

Primary Efficacy Analysis:

The primary analysis will be performed using the primary analysis set. Osteoid volume (osteoid volume/bone volume, OV/BV) at baseline, 48 weeks, and the percent change from baseline at 48 weeks will be summarized.

Secondary Efficacy Analyses:

The key secondary endpoint will be summarized as the proportion of subjects who achieve a serum phosphorus level above the LLN (2.5 mg/dL [0.81 mmol/L]), at 2 weeks post-dose (between baseline and Week 24, on average). Additional analyses of serum phosphorus including observed values, change from baseline, percent change from baseline over time and area under the curve will be summarized. Similar analyses will be performed for 1,25(OH)2D, urinary phosphorus, TmP/GFR, and TRP. Additional histomorphometric indices (O.Th, OS/BS, and MLt) and the percent change from baseline at 48 weeks will be summarized.

In general, observed values at baseline and end of study, and percent changes from baseline to end of the study for bone quality parameters and markers of bone turnover will be summarized.

Safety Analysis:

All AEs will be coded using the Medical Dictionary for Regulatory Activities (MedDRA).
The incidence and frequency of AEs will be summarized by System Organ Class, Preferred Term, severity, and relationship to treatment. The incidence and frequency of AEs and SAEs will be summarized. A by-subject listing will be provided for those subjects who experience a SAE, including death, or experience an AE associated with early withdrawal from the study or study drug treatment.

Clinical laboratory data will be summarized by the type of laboratory test. The frequency and percentage of subjects who experience abnormal clinical laboratory results (i.e., outside of reference ranges) and/or clinically significant abnormalities will be presented for each clinical laboratory measurement.

A review of safety data will be conducted by the Data Monitoring Committee (DMC) periodically through the end of the Treatment Period (Week 48). During Treatment Extension Periods I and II, safety data will be reviewed by the Ultragenyx Study Safety Review Team (SSRT) on an ongoing basis.

Table 2.1: Schedule of Events—Screening and Baseline Visits

	Screening[1]			Baseline[1]		
**VISIT TYPE/NUMBER**		**TC 1**	**TC 2**	**V1**		
**STUDY WEEK or DAY**	**Week −4**	**Week −3**	**Week −1**	**Day -2**	**Day -1**	**Day/Wk 0**
Informed Consent, Inclusion/Exclusion Criteria	X					
Medical History, Demographics, Height [2]	X					
PHEX Mutation Analysis [3,4]				X		
Tetracycline HCl or Demeclocycline Label [5]		X	X			
Bone Biopsy [1]					X	
Bone Turnover Markers [6]				X		
Serum Phosphorus, Calcium, iPTH, Creatinine/eGFR [6]	X			X		
Serum 1,25(OH)$_2$D [6]				X		
Serum iFGF23	X[4]			X		
2-hr Urine [6,7]	X			X		
24-hr Urine [6,7]				X		
BPI, BFI [8,9]	X			X		
Skeletal survey [10]				X		
Anti-KRN23 (ADA)						X
Vital Signs [11]	X			X	X	X
Physical Examination	X			X		
Weight[12]				X		
Renal Ultrasound [1]	X					
ECHO, ECG [1]				X		
Chemistry, Hematology (with PT/PTT), Urinalysis/pregnancy test[6, 13, 14]	X			X		
Concomitant Medications	X			X	X	X
Adverse Events	X			X	X	X
**KRN23 ADMINISTRATION**						**X**

Table 2.2: Schedule of Events—Open Label Treatment Period (Weeks 1 – 48)

VISIT TYPE/NUMBER[15]	HH V2	V3	V4	HH V5	HH V6	V7	HH V8	HH V9	HH V10	HH V11	V12	V13	HH V14	HH V15	V16	HH V17	HH V18	TC 3	TC 4	V19[1]	
WEEK	1	2	4	6	8	12	14	16	20	21	22	24	28	32	36	40	44	45	47	48[1]	
																				Day 1	Day 2
Tetracycline HCl or Demeclocycline Label[5]																		X	X		
Bone Biopsy [1]																					X[16]
Bone Turnover Markers [6]						X						X								X	
Serum Phosphorus, Calcium [6]	X	X	X	X		X	X		X	X	X	X	X		X					X	
Serum $1,25(OH)_2D$ [6]	X	X	X						X	X	X	X								X	
iPTH[6]	X	X	X						X	X	X									X	
Serum iFGF23[6]												X								X	
Serum Creatinine/eGFR[6]		X	X			X					X	X								X	
2-hr Urine [6,7]		X	X			X					X	X								X	
24-hr Urine [6,7]						X						X			X					X	
BPI, BFI [8,9]						X						X			X					X	
Targeted Radiography [10]						X						X			X					X	
KRN23 PK	X	X	X							X	X	X								X	
Anti-KRN23 (ADA)			X									X								X	
Vital Signs [11]			X			X						X			X					X	
Weight [12]						X						X			X					X	
Physical Examination						X						X			X					X	
Renal Ultrasound												X								X	
ECHO, ECG												X								X	
Chemistry, Hematology (with PT/PTT), Urinalysis [6, 13]			X			X						X			X					X	
Urine Pregnancy Test [14]			X		X	X		X	X			X	X	X	X	X	X			X	

VISIT TYPE/NUMBER[15]	HH V2	V3	V4	HH V5	HH V6	V7	HH V8	HH V9	HH V10	HH V11	V12	V13	HH V14	HH V15	V16	HH V17	HH V18	TC 3	TC 4	V19[1]	
**WEEK**	**1**	**2**	**4**	**6**	**8**	**12**	**14**	**16**	**20**	**21**	**22**	**24**	**28**	**32**	**36**	**40**	**44**	**45**	**47**	**48[1]**	
																				**Day 1**	**Day 2**
Concomitant Medications	X	X	X	X	X	X	X	X	X	X	X	X	X	X	X	X	X			X	X
Adverse Events	X	X	X	X	X	X	X	X	X	X	X	X	X	X	X	X	X			X	X
**KRN23 ADMINISTRATION**			X		X	X		X	X			X	X	X	X	X	X			X	

Table 2.3: Schedule of Events – Treatment Extension Period I (Weeks 49-96), Early Termination, and Safety Follow-up

	Treatment Extension Period I														Early Termination				Safety Follow-up
**VISIT TYPE/NUMBER[15]**	**HH V20**	**HH V21**	**V22**	**HH V23**	**HH V24**	**HH V25**	**V26**	**HH V27**	**HH V28**	**V29**	**HH V30**	**HH V31**	**HH V32**	**V33[1] EOS I[19]**	**ET[1, 20]**				**Follow-up Phone Call[18]**
															**TC 5**	**TC 6**	**ET**		
**WEEK**	**52**	**56**	**60**	**64**	**68**	**70**	**72**	**76**	**80**	**84**	**88**	**92**	**94**	**96**	**Week -3**	**Week -1**	**Day 1**	**Day 2**	
Bone Biopsy[1]																		X[16]	
Tetracycline HCl or Demeclocycline Label[5, 16]															X	X			
Bone Turnover Markers [6]							X							X			X		
Serum Phosphorus, Calcium [6]			X			X	X			X			X	X			X		
Serum 1,25(OH)$_2$D [6]			X			X	X			X			X	X			X		
iPTH[6]			X			X	X			X			X	X			X		
Serum iFGF23[6]														X			X		
Serum Creatinine/eGFR[6]			X				X			X				X			X		
2-hr Urine [6,7]			X				X			X				X			X		
24-hr Urine [6,7]							X							X			X		
BPI, BFI [8,9]							X							X					
Targeted Radiography [10]	Only at clinic visits following a newly diagnosed fracture[17]																		
Anti-KRN23 (ADA)							X							X			X		
KRN23 PK						X	X							X			X		
Vital Signs [11]			X				X			X				X			X		
Weight [12]							X							X			X		
Physical Examination							X							X			X		
Renal Ultrasound							X							X			X		
ECHO, ECG							X							X			X[17]		
Chemistry, Hematology (with PT/PTT), Urinalysis [6, 13]			X				X			X				X			X[13]		
Urine Pregnancy Test [14]	X	X	X	X	X		X	X	X	X	X	X		X			X		
Concomitant Medications	X	X	X	X	X	X	X	X	X	X	X	X	X	X			X	X	X
Adverse Events	X	X	X	X	X	X	X	X	X	X	X	X	X	X			X	X	X

	Treatment Extension Period I														Early Termination				Safety Follow-up
VISIT TYPE/NUMBER[15]	HH V20	HH V21	V22	HH V23	HH V24	HH V25	V26	HH V27	HH V28	V29	HH V30	HH V31	HH V32	V33[1] EOS I[19]	ET[1, 20]				Follow-up Phone Call[18]
															TC 5	TC 6	ET		
WEEK	52	56	60	64	68	70	72	76	80	84	88	92	94	96	Week -3	Week -1	Day 1	Day 2	
**KRN23 ADMINISTRATION[21]**	X	X	X	X	X		X	X	X	X	X	X		X[21]					

Footnotes to Table 2.1, Table 2.2, and Table 2.3

1 The Baseline visit should occur no more than 31 days following the Screening visit. The bone biopsy must be completed on a day with no other procedures, and prior to dosing on Day 0. Renal ultrasound, ECHO, ECG, and x-rays may be performed within 3 days of indicated clinic visit to accommodate scheduling availability. The Baseline, Week 48, and Week 96 (or Early Termination; ET) assessments may be completed in any reasonable order (except where indicated) to allow for flexibility in scheduling. However, all Screening/Baseline assessments and inclusion/exclusion criteria must be satisfied prior to dosing.

2 Medical history will include any available previous *PHEX* mutation analysis results for the subject or relevant family members with appropriate X-linked inheritance pattern. Height (in meters) will be obtained using a stadiometer (without shoes).

3 *PHEX* mutation analysis will be performed for all qualified subjects. If the Baseline result for *PHEX* mutation analysis is negative or inconclusive (i.e., No Mutation, Likely Benign, Variant of Uncertain Significance, or Possibly Pathogenic), and informed consent is provided by the subject, reflexive genetic testing will be performed to assess additional genes associated with phenotypes overlapping with XLH. A new blood sample for genetic analysis may be collected if necessary.

4 For patients without prior *PHEX* mutation analysis who fail screening on the basis of the Kainos iFGF23 assay, *PHEX* analysis may be conducted before the baseline visit

5 Available test results related to eligibility will be communicated to the subject within 1 week of the Screening visit by telephone call (TC1). If eligible, tetracycline HCl (or demeclocycline) will be provided to the subject with instructions for administration. The bone biopsy should be performed 5 days after the last dosing day for tetracycline or demeclocycline: The first dose should be given on days -20, -19, and -18 following TC1, and the second dose given on days -8, -7, and -6 following a second telephone call (TC2). The same instructions apply to TC3 and TC4 prior to the Week 48 (or TC5 and TC6 prior to ET if applicable) bone biopsy.

6 Blood and urine to be collected after a minimum overnight fasting time of 8 hours and prior to drug administration (if applicable).

7 Both 2-hr and 24-hr urine will be used for measurements of urinary phosphorus, creatinine and calcium; 2-hr urine will be used for the derivation of TmP/GFR and TRP.

8 Only the BPI Question 3 (Worst Pain) will be administered at Screening for eligibility. The complete short-form BPI and BFI will be administered at all other indicated visits.

9 Administer BPI first followed by BFI. The BPI and BFI should be administered prior to the performance of any invasive procedures.

10 X-rays will be taken at locations pre-determined by skeletal survey (chest, lateral spine, right and left hand/wrist, right and left humerus, right and left radius/ulna, right and left femur/pelvis, right and left tibia/fibula, and right and left foot). If active pseudofracture(s) or fracture(s) are detected at baseline,

targeted X-rays at that location will be obtained as indicated. During the Treatment Extension Period I, targeted x-rays will only be performed at clinic visit(s) to follow healing of any newly diagnosed fractures.

[11] Vital sign measurements consist of seated systolic/diastolic BP measured in millimeters of mercury (mm Hg; 2 measurements separated by 15 minutes), HR (beats per minute), respiration rate (breaths per minute), and temperature in degrees Celsius (°C). Obtain at the beginning of the visit before any additional assessments are completed

[12] Weight will be recorded in kilograms. Dose of study drug must be adjusted if weight increases by more than 20% from baseline.

[13] Serum chemistry panels may include PD parameters (i.e. serum phosphorus), and safety parameters of interest (i.e. calcium) to avoid duplication of testing. Screening and Week 48 Day1 hematology will include Prothrombin time/Partial thromboplastin time (PT/PTT). ET hematology will include PT/PTT if a bone biopsy is also scheduled (if a subject discontinues the study between 24 and 48 weeks)

[14] Urine pregnancy test for women of childbearing potential only. A serum pregnancy test will be performed in the event of a positive or equivocal urine pregnancy test result.

[15] During the Open-Label Treatment Period (Weeks 1 – 48) and Treatment Extension Period I (Weeks 49-96) subjects will return to the clinic and/or have home health (HH) visits as indicated (± 5 days). HH visits may also be conducted at the clinic depending on subject proximity to the investigational site and local availability of home health care resources.

[16] Bone biopsies will not be performed at Week 48 (or at ET) for subjects who do not have evidence of osteomalacia on the initial biopsy. Bone biopsies will not be performed at the ET Visit if the subject discontinues within 6 months of the baseline biopsy or if the subject terminates the study after 48 weeks. Prior to ET biopsy, tetracycline HCl (or demeclocycline) will be provided to the subject with instructions for administration. The bone biopsy should be performed 5 days after the last dosing day for tetracycline or demeclocycline The first dose should be given on days -20, -19, and -18 following TC5, and the second dose given on days -8, -7, and -6 following a subsequent telephone call (TC6) prior to the ET biopsy (if eligible).

[17] Targeted X-rays and ECHO will not be performed at ET if the assessment was conducted within 3 months of termination. Targeted X-rays will not be taken at ET if the subject terminates the study after 48 weeks.

[18] To be completed for all subjects who complete the EOS I or ET Visit and are not immediately continuing KRN23 treatment under commercial use or another mechanism. The site personnel will initiate a Safety Follow-up telephone call (TC) 4 weeks (+ 5 days) after the EOS I/ET Visit to collect information on whether KRN23 treatment has been started through another mechanism and, if not, any ongoing or new AEs, SAEs, or concomitant medications. Appropriate follow-up of AEs/SAEs should continue until all safety concerns, in the Investigator's opinion, are resolved. If the subject is not continuing KRN23 therapy under commercial use or another mechanism at that time, then site personnel will initiate a subsequent Safety Follow-up TC at 8 weeks (+ 5 days) after the EOS I/ET Visit to collect information on whether KRN23 treatment has been started through another mechanism and, if not, any ongoing or new AEs, SAEs, or concomitant medications.

[19] For subjects at study sites outside of the US, their final scheduled site visit at Week 96 will be their End of Study (EOS) efficacy visit; this visit is referred to as EOS I. No study drug will be administered to these subjects at this visit under the current protocol. If the subject will be continuing KRN23 treatment under commercial use or another mechanism, the first dose of that treatment should not be administered until after completion of all of the EOS I Visit assessments.

[20] If a subject will be continuing KRN23 treatment under commercial use or another mechanism, the first dose of that treatment should not be administered until after completion of all of the ET Visit assessments.

[21] Study drug will be administered every 28 days (± 5 days and no fewer than 14 days apart). For subjects outside the US, no study drug will be administered at the Week 96 Visit.

Table 2.4: Schedule of Events – Treatment Extension Period II (Week 97 – End of Study II) Plus Safety Follow-up (Sites in United States Only)

VISIT FREQUENCY or VISIT ID [1]	Q4W (HH Visit)	Q12W	Q24W	EOS II[12]	Safety Follow-up TC[13]
Serum Phosphorus [2]		X		X	
Serum Calcium [2]		X		X	
Serum 1,25(OH)$_2$D [2]		X		X	
Serum iPTH [2]		X		X	
Serum iFGF23				X	
Serum Creatinine/eGFR [2]				X	
Bone turnover markers [2]				X	
2-hr Urine [2, 3]				X	
24-hr Urine [2, 3]				X	
Targeted X-ray [4]	Only at clinic visits following a newly diagnosed fracture				
BPI, BFI [5, 6]			X	X	
KRN23 PK [7]				X	
Anti-KRN23 (ADA)				X	
Vital Signs [8]		X		X	
Physical Examination, Weight [9]		X		X	
Renal Ultrasound				X	
ECHO, ECG				X	
Chemistry, Hematology, Urinalysis [2]		X		X	
Urine Pregnancy Test [10]	X			X	
AEs/Concomitant Medication	X			X	X
KRN23 Administration [11]	X				

1 During Treatment Extension Period II (Weeks 97 – End of Study II for subjects at sites in the US only), clinic visits will occur at 12-week intervals (± 5 days). Subjects have home health (HH) visits at 4-week intervals (± 5 days). HH visits may also be conducted at the clinic depending on subject proximity to the investigational site and local availability of home health care resources.

2 Blood and urine to be collected after a minimum overnight fasting time of 8 hours and prior to drug administration (if applicable). Peak serum phosphorus may be collected as an unscheduled laboratory assessment if necessary.

[3] Both 2-hr and 24-hr urine will be used for measurements of urinary phosphorus, creatinine and calcium; 2-hr urine will be used for the derivation of TmP/GFR and TRP.

[4] Targeted x-rays will only be performed at clinic visit(s) to follow healing of any newly diagnosed fractures.

[5] Administer BPI first followed by BFI. The BPI and BFI should be administered prior to the performance of any invasive procedures.

[6] The complete short-form BPI and BFI will be administered

[7] Obtain blood samples for KRN23 measurement prior to drug administration (if applicable).

[8] Vital sign measurements consist of seated systolic/diastolic BP measured in millimeters of mercury (mm Hg; 2 measurements separated by 15 minutes), HR (beats per minute), respiration rate (breaths per minute), and temperature in degrees Celsius (°C). Obtain at the beginning of each indicated visit before any additional assessments are completed.

[9] Weight will be recorded in kilograms. Dose of study drug must be adjusted if weight changes by more than 20% from baseline.

[10] For women of childbearing potential only. A serum pregnancy test will be performed in the event of a positive or equivocal urine pregnancy test result

[11] Study drug will be administered every 28 days (± 5 days and no fewer than 14 days apart)

[12] For subjects who complete Treatment Extension Period II, their final End of Study (EOS) efficacy visit is referred to as EOS II and should occur before 30 September 2018. If a subject will be continuing KRN23 treatment under commercial use or another mechanism, the first dose of that treatment should not be administered until after completion of all of the EOS II Visit assessments.

[13] To be completed for all subjects who complete the EOS II Visit and are not immediately continuing KRN23 treatment under commercial use or another mechanism. The site personnel will initiate a Safety Follow-up telephone call (TC) 4 weeks (+ 5 days) after the EOS II/ET Visit to collect information on whether KRN23 treatment has been started through another mechanism and, if not, any ongoing or new AEs. SAEs, or concomitant medications. Appropriate follow-up should continue until all safety concerns, in the Investigator’s opinion, are resolved. If the subject is not continuing KRN23 therapy under commercial use or another mechanism at that time, then site personnel will initiate a subsequent Safety Follow-up TC at 8 weeks (+ 5 days) after the EOS II/ET Visit to collect information on whether KRN23 treatment has been started through another mechanism and, if not, any ongoing or new AEs, SAEs, or concomitant medications.

3 TABLE OF CONTENTS

1 TITLE PAGE 1
2 SYNOPSIS 4
3 TABLE OF CONTENTS 22
4 LIST OF ABBREVIATIONS AND DEFINITION OF TERMS 26
5 INTRODUCTION 29
5.1 Overview of XLH 30
5.2 Brief Overview of KRN23 Development 31
5.2.1 Brief Description of KRN23 31
5.2.1.1 Mechanism of Action in XLH 31
5.2.2 Nonclinical Studies 31
5.2.3 Clinical Studies 33
5.3 Summary of Overall Risks and Potential Benefits 34
5.4 Study Rationale 34
5.5 Primary Efficacy Hypothesis 35
6 STUDY OBJECTIVES 36
7 INVESTIGATIONAL PLAN 37
7.1 Overall Study Design and Plan 37
7.2 Discussion of Study Design, Including Choice of Control Group 37
7.3 Selection of Study Population 38
7.3.1 Inclusion Criteria 39
7.3.2 Exclusion Criteria 40
7.3.3 Removal of Subjects from Therapy or Assessment 42
7.3.3.1 Stopping Rules 42
7.4 Treatments 43
7.4.1 Investigational Product 43
7.4.2 Reference Therapy 44

7.4.3 Selection of Doses and Study Duration....44
7.4.3.1 Study Duration....45
7.4.4 Method of Assigning Subjects to Treatment Groups....46
7.4.5 Blinding....46
7.4.6 Treatment Compliance....46
7.5 Study Procedures and Assessments....46
7.5.1 Efficacy Measures....47
7.5.1.1 Iliac Crest Bone Biopsy....48
7.5.1.2 Biochemical Markers....48
7.5.1.3 Patient Reported Outcomes....49
7.5.1.4 Targeted Radiography and Skeletal Survey....50
7.5.2 Drug Concentration Measurements....51
7.5.3 Safety Measures & General Assessments....51
7.5.3.1 Medical History....51
7.5.3.2 Vital Signs....52
7.5.3.3 Weight and Height....52
7.5.3.4 Physical Examination....52
7.5.3.5 Renal Ultrasound and Glomerular Filtration Rate....52
7.5.3.6 Echocardiogram....53
7.5.3.7 Electrocardiogram....53
7.5.3.8 Clinical Laboratory Tests for Safety....53
7.5.3.9 Pregnancy Testing....55
7.5.3.10 Prior and Concomitant Therapy....56
7.5.3.11 Adverse Events....57
7.5.4 Appropriateness of Measures....57
7.6 Statistical Methods and Determination of Sample Size....58
7.6.1 Determination of Sample Size....59

7.6.2 Analysis Populations 59
7.6.2.1 Primary Analysis Set....59
7.6.2.2 Full Analysis Set 59
7.6.2.3 Safety Analysis Set 59
7.6.2.4 Treatment Extension Analysis Set 59
7.6.3 General Principles 59
7.6.3.1 Subject Accountability....60
7.6.3.2 Demographic and Baseline Characteristics 60
7.6.3.3 Baseline....60
7.6.4 Efficacy Endpoints and Analyses....60
7.6.4.1 Primary Efficacy Endpoint 60
7.6.4.2 Key Secondary Endpoint 61
7.6.4.3 Secondary Endpoints 61
7.6.4.4 Exploratory Endpoints 62
7.6.5 Safety Analyses 62
7.6.6 Interim Analysis 63
7.6.7 Data Monitoring Committee 63
8 STUDY CONDUCT....64
8.1 Ethics....64
8.1.1 Institutional Review Board or Ethics Committee....64
8.1.2 Ethical Conduct of Study 64
8.1.3 Subject Information and Consent....64
8.2 Investigators and Study Administrative Structure 65
8.3 Investigational Product Accountability....65
8.4 Data Handling and Record Keeping 66
8.4.1 Case Report Forms and Source Documents....66
8.4.2 Data Quality Assurance....66

8.4.3 Record Retention 67
8.5 Reporting and Follow-up of Adverse Events 67
8.5.1 Definition of Adverse Events 67
8.5.2 Severity of Adverse Events 68
8.5.3 Relationship of Adverse Events to Study Drug 69
8.5.4 Adverse Event Reporting to Ultragenyx 69
8.5.4.1 General 69
8.5.4.2 Serious Adverse Events, Serious Adverse Drug Reactions, and Requirements for Immediate Reporting 70
8.5.4.3 Urgent Safety Reporting 70
8.5.4.4 Adverse Drug Reaction Reporting 71
8.5.4.5 Pregnancy in Subject or Partner 71
8.5.4.6 Review of Safety Data 72
8.5.4.7 Safety Contact Information 73
8.6 Financing and Insurance 73
8.7 Publication Policy 73
9 REFERENCES 74
10 SIGNATURE PAGE 78

List of Tables

Table 2.1: Schedule of Events—Screening and Baseline Visits 14
Table 2.2: Schedule of Events—Open Label Treatment Period (Weeks 1 – 48) 15
Table 2.3: Schedule of Events – Treatment Extension Period I (Weeks 49-96), Early Termination, and Safety Follow-up 17
Table 2.4: Schedule of Events – Treatment Extension Period II (Week 97 – End of Study) Plus Safety Follow-up (Sites in United States Only) 20
Table 7.5.3.8.1: Clinical Laboratory Assessments for Safety 54

4 LIST OF ABBREVIATIONS AND DEFINITION OF TERMS

Abbreviations

1,25$(OH)_2$D	1,25-dihydroxyvitamin D
25(OH)D	25-hydroxyvitamin D
ADA	anti-drug antibodies
AE	adverse event
ALP	alkaline phosphatase
AUC	area under the curve
BALP	bone-specific alkaline phosphatase
BFI	Brief Fatigue Inventory
BFI-Q3	Brief Fatigue Inventory Question 3 (worst fatigue in 24 hours)
BFR	bone formation rate
BPI	Brief Pain Inventory
BUN	blood urea nitrogen
CBC	complete blood count
°C	degrees Celsius
CFR	Code of Federal Regulations
CKD-EPI	Chronic Kidney Disease Epidemiology Collaboration
CRF	case report form
CTx	carboxy-terminal cross-linked telopeptide of type I collagen
dL	deciliter
DMC	Data Monitoring Committee
EC	Ethics Committee
ECG	electrocardiogram
ECHO	echocardiogram
ECLA	electrochemiluminescent assay
EDC	electronic data capture
eGFR	estimated glomerular filtration rate
EOS	End of Study (Visit)
ET	Early Termination (Visit)
EU	European Union
EudraCT	European Union Drug Regulating Authorities Clinical Trials
FDA	Food and Drug Administration
FGF23	fibroblast growth factor 23
GCP	Good Clinical Practice

GFR	glomerular filtration rate
GLP	Good Laboratory Practice
GMP	Good Manufacturing Practice
HIPAA	Health Insurance Portability and Accountability Act
Hyp	hypophosphatemic
IB	Investigator's Brochure
ICF	Informed Consent Form
ICH	International Conference on Harmonisation of Technical Requirements for Pharmaceuticals for Human Use
iFGF23	intact FGF23
IgG1	immunoglobulin G isotype 1
IND	Investigational New Drug (application)
IRB	Institutional Review Board
iPTH	intact parathyroid hormone
IV	intravenous
kDa	kilodalton
kg	kilogram
KHK	Kyowa Hakko Kirin Co. Ltd.
L	liter
LLN	lower limit of normal
LVH	left ventricular hypertrophy
mAb	monoclonal antibody
MAR	mineral apposition rate
MLt	mineralization lag time
MedDRA	Medical Dictionary for Regulatory Activities
mg	milligram
mm Hg	millimeters of mercury
mmol	millimole
MS/BS	mineralizing surface/bone surface
NaPi-IIa	type IIa sodium/phosphate cotransporter
NaPi-IIc	type IIc sodium/phosphate cotransporter
NCI CTCAE	National Cancer Institute Common Terminology Criteria for Adverse Events
NOAEL	no observed adverse effect level
OS/BS	osteoid surface/bone surface
O.Th	osteoid thickness

OV/BV	osteoid volume/bone volume
P1NP	procollagen type 1 N-propeptide
PD	pharmacodynamic(s)
PHEX	Phosphate regulating gene with homology to endopeptidases located on the X chromosome
PK	pharmacokinetic(s)
PRO	patient-reported outcome
PT	prothrombin time
PTT	partial thromboplastin time
PTH	parathyroid hormone
Q4W	monthly; every four weeks
RBC	red blood cell
RSI	Reference Safety Information
SAE	serious adverse event
SAP	statistical analysis plan
SC	subcutaneous
SSRT	Study Safety Review Team
SUSAR	suspected unexpected serious adverse reactions
TC	telephone call
TmP/GFR	ratio of renal tubular maximum reabsorption rate of phosphate to glomerular filtration rate
TRP	tubular reabsorption of phosphate
ULN	upper limit of normal
US	United States
WBC	white blood cell
XLH	X-linked hypophosphatemia

Definition of Terms

Investigational Product is defined as, “A pharmaceutical form of an active ingredient or placebo being tested or used as a reference in a clinical trial, including a product with a marketing authorization when used or assembled (formulated or packaged) in a way different from the approved form, or when used for an unapproved indication, or when used to gain further information about an approved use” (from International Conference on Harmonisation of Technical Requirements for Pharmaceuticals for Human Use [ICH] Harmonised Tripartite Guideline E6: Guideline for Good Clinical Practice).

The terms “Investigational Product” and “study drug” may be used interchangeably in the protocol.

5 INTRODUCTION

X-linked hypophosphatemia (XLH) is a disorder of hypophosphatemia, renal phosphate wasting, and defective bone mineralization caused by inactivating mutations in the *PHEX* gene (phosphate-regulating gene with homologies to endopeptidases on the X chromosome). In the absence of functional PHEX, release of fibroblast growth factor 23 (FGF23) by osteocytes is greatly increased. Excess circulating FGF23 leads to decreased reabsorption of phosphate and reduced 1,25-dihydroxyvitamin D ($1,25(OH)_2D$) production, resulting in decreased intestinal absorption of calcium and phosphate. Chronic hypophosphatemia leads to defective bone mineralization and consequently to rickets in children and osteomalacia in adults, the two major pathologic features of XLH.

Adult patients with XLH have significant morbidity that increases with age. In adulthood, XLH progresses into a disease of persistent and often clinically significant osteomalacia as a result of chronic low phosphorus, causing bone and joint pain and pseudofractures in a large proportion of patients (Reid et al. 1989). With age, osteoarthritis often develops, in part due to degenerative changes resulting from joint misalignment that occurred with childhood rickets. Mineralization of tendons/ligaments (enthesopathy) and recurrent dental abscesses may also occur. Adults with XLH also have muscle dysfunction, likely due to the importance of phosphate in energy metabolism, which may contribute significantly to the fatigue and weakness expressed by these patients (Reid et al. 1989), (Veilleux et al. 2012), (Sabbagh et al. 2008).

The current standard of care for XLH in children consists of oral phosphate and vitamin D (calcitriol) replacement, which is typically discontinued in adolescence once longitudinal bone growth is complete. Treatment can improve rickets but provides limited benefit and does not treat the underlying cause (Carpenter et al. 2011). No consensus exists regarding treatment of XLH in adults, despite the significant morbidity in many patients. By blocking the action of the aberrantly elevated FGF23, phosphorus homeostasis could potentially be normalized, offering an effective and safe treatment option for patients with XLH.

Proof-of-concept studies in a relevant murine model support the use of an anti-FGF23 monoclonal antibody (mAb) as a treatment for XLH. Experiments in both juvenile and adult hypophosphatemic (Hyp) mice provided evidence that treatment with an anti-FGF23 mAb normalized or ameliorated many of the characteristic abnormalities associated with XLH (Aono et al. 2009), (Aono et al. 2011). KRN23 is a fully human IgG_1 mAb that binds to and inhibits FGF23. The Sponsor and development partner, Kyowa Hakko Kirin Co. Ltd. (KHK) are investigating KRN23 as a potential therapeutic candidate for the treatment of XLH, a disease distinguished by high levels of serum FGF23.

KHK has conducted a series of nonclinical pharmacology, pharmacokinetic (PK), and toxicology studies supporting the investigation of KRN23 in adults and children.
Three clinical studies have been completed in adult patients with XLH: a single dose Phase 1 safety and tolerability study of KRN23, a repeat dose Phase 1/2 dose escalation study, and an associated treatment extension study. An additional open-label long-term extension study is

ongoing. A randomized, double-blind, placebo-controlled, Phase 3 study is planned in symptomatic adults with XLH to establish the efficacy and safety profile of KRN23 treatment. The study also seeks to establish the beneficial impact of KRN23 treatment on functional and patient-reported outcomes (PROs) including pain, stiffness, fatigue, and health-related quality of life. The current open-label study will examine the efficacy of KRN23 on bone quality and skeletal health, including the effects on osteomalacia at the tissue level, and provide an additional assessment of safety.

5.1 Overview of XLH

XLH is a rare genetic disorder that is serious, chronically debilitating and represents an unmet medical need. This genetic deficiency is estimated to occur in about 1:20,000 live births (Burnett et al. 1964), (Imel et al. 2005). XLH is the most common inherited form of rickets and the most common inherited defect in renal tubular phosphate transport. XLH is transmitted as an X-linked dominant disorder (Dixon et al. 1998). Mutations resulting in the loss of function of PHEX form the genetic basis for XLH (Carpenter et al. 2011). More than 300 different *PHEX* gene mutations have been identified in patients with XLH (PHEXdb); however, few definitive correlations have been observed between specific mutations and phenotypic severity.

Patients with XLH have hypophosphatemia due to excessive FGF23 levels (Jonsson et al. 2003), (Yamazaki et al. 2002); however, the precise mechanism by which PHEX disruption results in elevated FGF23 is complex and not fully understood (Rowe 2012), (Carpenter et al. 2011). FGF23 plays an important role as a specific regulator of serum phosphorus; its major function is to reduce serum phosphorus levels by inhibiting renal proximal tubular phosphate reabsorption (Fukumoto 2008), (Razzaque et al. 2007). FGF23 also decreases serum 1,25(OH)$_2$D levels by inhibiting 1-alpha-hydroxylase activity in the kidney, thereby decreasing intestinal absorption of phosphate and calcium. The actions of FGF23 on the tubular reabsorption and intestinal absorption via vitamin D metabolism lead to a decrease in serum phosphorus levels.

Patients with XLH often present during childhood with rickets due to hypophosphatemia and frequently develop skeletal abnormalities (e.g. bowed legs), impaired growth, and short adult stature (Tenenhouse et al. 2001). As young patients age and progress into adulthood, the symptom pattern evolves due to decreased phosphate requirements for bone growth.
Adult XLH patients suffer from bone pain and osteomalacia, increased risk of bone fractures, joint abnormalities and joint pain, enthesopathy, and osteoarthritis (Carpenter et al. 2011).
No consensus exists regarding treatment of adult patients with XLH (Linglart et al. 2014).
The use of oral phosphate and vitamin D may be initiated for the treatment of osteomalacia, bone/joint pain and pseudofractures in symptomatic patients, although evidence of efficacy is limited (Sullivan et al. 1992). This treatment protocol requires frequent and continued monitoring of patients. Serum and urine mineral metabolite levels and imaging studies are required to assess toxicity and secondary complications, including nephrocalcinosis, hypercalciuria, and hyperparathyroidism (Carpenter et al. 2011).

5.2 Brief Overview of KRN23 Development

A brief overview of existing information on KRN23 is provided below; a comprehensive review of the data is contained in the Investigator's Brochure (IB) provided by Ultragenyx Pharmaceutical Inc. (Ultragenyx), which should be reviewed prior to initiating the study.

5.2.1 Brief Description of KRN23

KRN23 is a recombinant human IgG_1 mAb that binds to and inhibits the activity of FGF23. KRN23 is expressed in Chinese Hamster Ovary dihydrofolate reductase-deficient cells. The secreted KRN23 antibody is recovered from the culture medium and purified using a series of chromatographic and filtration steps. Based on the amino acid sequence, the predicted molecular mass of KRN23 is approximately 140 kilodaltons (kDa). Nonclinical studies demonstrated KRN23 possesses high binding affinity to the N-terminal domain of FGF23. KRN23 binds to FGF23 from humans, cynomolgus monkeys and rabbits, but not to other species tested.

5.2.1.1 Mechanism of Action in XLH

Patients with XLH have hypophosphatemia due to excessive serum FGF23 levels. FGF23 reduces serum phosphorus levels by two distinct mechanisms of action (Fukumoto 2008), (Razzaque et al. 2007), (Yamazaki et al. 2008). The primary mechanism is to inhibit phosphate reabsorption in the proximal tubule of the kidney. The secondary mechanism is to decrease phosphate absorption by the small intestine through the inhibition of $1,25(OH)_2D$ production in the kidney.

KRN23 has the potential to block or reduce FGF23 action and improve phosphate homeostasis in XLH patients. KRN23 binds the amino-terminal domain of FGF23 that interacts with the FGF-binding portion of the combination FGFR1/Klotho receptor, preventing FGF23 from binding to and signaling from its receptor. Both intact and fragmented FGF23 polypeptides are immunoprecipitated with KRN23 (Yamazaki et al. 2008). By inhibiting FGF23, KRN23 restores tubular reabsorption of phosphate (TmP/GFR) from the kidney and increases the production of $1,25(OH)_2D$ that also enhances intestinal absorption of phosphate. Both actions increase serum phosphorus levels, which is expected to improve bone mineralization and reduce the diverse bone and non-bone manifestations associated with hypophosphatemia in XLH patients. Bone histomorphometry is the gold standard to evaluate changes in osteomalacia as it allows an assessment of bone quality at the tissue level.

5.2.2 Nonclinical Studies

The Hyp mouse is a murine homologue of XLH with a deletion in the 3' region of the *PHEX* gene (Liu et al. 2007), (Perwad et al. 2005). In addition to hypophosphatemia, rickets, and associated developmental abnormalities, these animals display elevated serum FGF23 levels and increased expression of FGF23 in the bone. Since KRN23 does not bind murine FGF23, the pharmacological effects of murine anti-FGF23 mAbs were examined in juvenile and

adult Hyp mice (Aono et al. 2009), (Aono et al. 2011). In juvenile Hyp mice, anti-FGF23 treatment corrected hypophosphatemia and ameliorated the rachitic bone phenotypes (Aono et al. 2009). Histomorphometry of secondary cancellous bones showed improvement in both static and dynamic parameters of osteoid accumulation. In adult Hyp mice, anti-FGF23 treatment increased serum phosphate and 1,25(OH)$_2$D levels, and increased grip strength and spontaneous movement (Aono et al. 2011). These studies provide proof-of-concept that treatment with antibodies targeting FGF23 may reverse or ameliorate characteristic abnormalities associated with XLH.

KRN23 binds to human, rabbit, and monkey FGF23 with comparable affinities. In a study conducted under Good Laboratory Practice (GLP) conditions, KRN23 cross-reactivity was evaluated against a full panel of human, rabbit (32 tissues), and cynomolgus monkey (33 tissues) tissues by immunohistochemistry. No specific KRN23 staining was observed, suggesting untoward direct-effects of KRN23 are not expected in any tissues of normal humans, rabbits, or cynomolgus monkeys.

A series of nonclinical pharmacology, PK, and toxicity studies have been conducted in rabbits and cynomolgus monkeys to support the use of KRN23 in adults and children. Findings of potential clinical significance and relevance to this protocol are summarized below; additional information is provided in the IB.

- The no observed adverse effect level (NOAEL) in a 40-week toxicity study in adult cynomolgus monkeys was 0.03 mg/kg KRN23 for males and 0.3 mg/kg KRN23 for females. The NOAEL in a 40-week toxicity study in juvenile cynomolgus monkeys and a single-dose study in rabbits was 0.3 mg/kg KRN23.
- Soft tissue and organ mineralization was a consistent finding associated with prolonged and excessive serum phosphate levels including the kidney where nephrocalcinosis was observed at the highest dose tested and reversibility of mineralization could not be established.
- The most prominent pharmacologic actions of KRN23 were dose-dependent changes in serum inorganic phosphorus and 1,25(OH)$_2$D in rabbits and juvenile, adult, and pregnant cynomolgus monkeys.
- No gross or histopathological abnormalities were observed at the intravenous (IV) infusion sites or subcutaneous (SC) injection sites in the 40-week repeat-dose toxicity studies in adult and juvenile cynomolgus monkeys.
- KRN23 demonstrated consistent and predictable PK behavior in both rabbits and cynomolgus monkeys based on the results of single- and repeat-dose studies where exposure was by either the IV or SC route.

The NOAEL was the same in juvenile and adult monkeys suggesting no difference in sensitivity to the adverse effects of KRN23. The results from single- and repeat-dose

toxicology studies in rabbits and juvenile, adult, and pregnant cynomolgus monkeys suggest the primary toxicological effects of KRN23 are associated with prolonged and excessive antagonism of the normal regulatory actions of FGF23 on renal tubular phosphate reabsorption and vitamin D metabolism.

5.2.3 Clinical Studies

Four clinical studies have been completed in adult patients with XLH: a single dose Phase 1 safety and tolerability study of KRN23 (KRN23-US-02), a single dose Phase 1 safety and tolerability study of KRN23 in Japan and Korea (KRN23-001), a repeat dose Phase 1/2 dose escalation study (KRN23-INT-002), and an associated treatment extension study (KRN23-INT-002). An additional open-label long-term extension study (UX023-CL203) is ongoing. A Phase 2 dose-finding, pharmacodynamic (PD) and safety study is also being conducted in pediatric XLH patients (UX023-CL201). A confirmatory randomized, placebo-controlled Phase 3 safety and efficacy study is being conducted in parallel with this study (UX023-CL303). Details of study parameters and available PK, PD, clinical efficacy, and safety results are provided in the IB.

Data from clinical studies to date are consistent with the proposed mechanism of action: that KRN23 blocks FGF23 action, leading to a sustained increase in serum phosphorus levels due to increased tubular reabsorption of phosphate (TmP/GFR) and increased intestinal absorption caused by increased 1,25$(OH)_2$D. Single and repeat-dose clinical studies indicate SC administration of KRN23 consistently increased and sustained serum phosphorus levels and TmP/GFR, without a major impact on urine calcium levels or vitamin D metabolism. The data from the extension study suggest KRN23 could provide sustained increases in serum phosphorus levels sufficiently such that improvements in bone physiology, structure, and function would be expected.

Repeated doses of KRN23 up to 1.0 mg/kg were well tolerated by adult XLH subjects throughout the Phase 1/2 dose escalation and associated treatment extension study. No deaths or life threatening treatment emergent adverse events (AEs) have been reported. In the extension study, serious adverse events (SAEs) reported for 3 subjects were unlikely to be or were not study drug related: breast cancer, hypertensive crisis, and cervical spinal stenosis. Throughout the extension study, treatment-related AEs were reported for 14 subjects (63.6%) treated with KRN23 and included injection site reaction (5 subjects, 22.7%), arthralgia (3 subjects, 13.6%), restless legs syndrome (3 subjects, 13.6%), and injection site pain (2 subjects, 9.1%). No discernible clinically significant trends of lab abnormalities suggestive of a treatment-related adverse effect were noted. Overall, no immunogenicity or patterns of dose-limiting toxicity have been associated with KRN23 treatment.

5.3 Summary of Overall Risks and Potential Benefits

KRN23, a fully human mAb that binds and inhibits FGF23, is being developed as a potential therapeutic candidate for XLH, a rare genetic disease associated with hypophosphatemia and elevated levels of FGF23. By blocking the activity of FGF23, KRN23 has the potential to restore phosphate, vitamin D, and bone metabolism homeostasis in patients with XLH, thereby improving osteomalacia, the pathologic hallmark of XLH in adults. This therapeutic approach directly targets the inherent dysregulation in XLH (i.e., excess FGF23). In contrast, supplementation therapy with phosphate and/or 1,25(OH)$_2$D, is only partially effective and carries a significant burden and risk of ectopic mineralization, particularly nephrocalcinosis.

Clinical studies to date have demonstrated that KRN23 treatment blocks FGF23 action and leads to a sustained increase in serum phosphorus levels due to increased TmP/GFR. Increased 1,25(OH)$_2$D was also observed, as expected, based on the inhibition of the excess of FGF23. Bone formation and resorption markers also increased. KRN23 was well tolerated in the population studied. No major safety concerns were observed; there was no evidence of immunogenicity, and no evidence of left ventricular hypertrophy (LVH) based on electrocardiogram (ECG), even though FGF23 levels were increased following KRN23 treatment. Although ectopic mineralization is a known risk related to XLH disease and is exacerbated by oral phosphate and/or 1,25(OH)$_2$D supplementation, KRN23 does not appear to be associated with progression of cardiac or renal ectopic mineralization beyond the natural course of pre-existing disease.

SC doses of KRN23 in the range from 0.3 to 1.0 mg/kg were able to achieve the desired PD effect in adults, which lasted approximately one month, positioning KRN23 as a drug that could be administered once-per-month by SC injection, which is a convenient and acceptable therapeutic regimen for a chronic condition.

In conclusion, KRN23 inhibits the effects of FGF23, restoring phosphate, vitamin D, and bone metabolism homeostasis. By positively modulating serum phosphorus, it is expected that the underlying osteomalacia will be resolved, leading to improved clinical outcomes including a reduction in pain, stiffness, fatigue, and fracture risk as well as the impact of these factors on the quality of life of adult patients with XLH. To date, KRN23 has a favorable safety profile without evidence of increased ectopic mineralization or other concerns associated with the excess of FGF23. KRN23 has the potential to be an effective and safe treatment option for patients with XLH.

5.4 Study Rationale

XLH is a disorder of hypophosphatemia, renal phosphate wasting, and defective bone mineralization caused by inactivating mutations in the *PHEX* gene (phosphate-regulating gene with homologies to endopeptidases on the X chromosome). In the absence of functional PHEX, release of FGF23 by osteocytes is greatly increased. Excess circulating FGF23 signals the kidney tubules to decrease phosphate reabsorption by down-regulating NaPi-IIa and NaPi-IIc in the tubular cells and suppressing 1,25(OH)$_2$D production, resulting in

decreased intestinal absorption of calcium and phosphate producing hypophosphatemia. In adults with XLH, a hallmark of the disease is osteomalacia, a defect in bone mineralization due to chronically low serum phosphorus. Osteomalacia is characterized by a lack of proper mineralization, a prolonged mineralization process, and an accumulation of osteoid tissue. It is also associated with a reduction in bone remodeling and reduced bone quality and is a key contributor to the symptoms of bone and joint pain and the non-traumatic pseudofractures and reduced quality of life experienced by adults with XLH.

KRN23 is a recombinant fully human IgG1 monoclonal antibody being developed to treat XLH by binding to FGF23 and inhibiting its activity, thereby increasing serum phosphorus levels and improving osteomalacia and related symptoms. Bone histomorphometry is the gold standard for evaluating changes in osteomalacia, thus it is important to characterize changes in bone biopsy parameters in response to KRN23 treatment. Bone biopsy studies in adults with XLH receiving oral phosphate and vitamin D therapy have shown incomplete healing of osteomalacia (Sullivan et al. 1992). In addition, there are no systematic studies in adults to show this regimen improves symptoms or outcomes, and there are concerns about the risk of long-term complications, particularly nephrocalcinosis, which increases with dose and duration of therapy. More efficacious, safer, and convenient therapies clearly are needed for adults who continue to experience XLH-related symptoms and complications.

A Phase 1 study established the PK profile of KRN23. A Phase 1/2 study and associated extension study evaluated the PD of KRN23 on phosphate metabolism and related measures of the phosphate-calcium mineral control system. The safety data from these studies have shown that KRN23 in single and repeated monthly doses up to 1.0 mg/kg was well tolerated by adult subjects with XLH. KRN23 sufficiently increased serum phosphorus levels such that improvements in bone physiology, structure, and function would be expected.

A randomized, double-blind, placebo-controlled Phase 3 study is being conducted to confirm the efficacy and safety of KRN23 treatment, and to establish the beneficial impact on patient-reported and functional outcomes in adults with XLH. The current open-label study will examine the efficacy of KRN23 on bone quality, including the effects on osteomalacia at the tissue level, and provide an additional assessment of safety. During the Treatment Extension Periods I and II, additional changes in efficacy outcomes and long-term safety will be assessed.

5.5 Primary Efficacy Hypothesis

Treatment with 1.0 mg/kg KRN23 monthly will improve XLH-associated osteomalacia as determined by osteoid volume (osteoid volume/bone volume, OV/BV).

6 STUDY OBJECTIVES

Primary Efficacy Objective:

Establish the effect of KRN23 treatment on improvement in XLH-associated osteomalacia as determined osteoid volume/bone volume.

Secondary Efficacy Objectives:

The key secondary efficacy objective is to establish the effect of KRN23 treatment on increasing serum phosphorus levels in adults with XLH

Other secondary efficacy objectives are to establish the effect of KRN23 treatment in adults with XLH on:

- Changes from baseline in additional histomorphometric parameters – including osteoid thickness (O.Th), osteoid surface/bone surface (OS/BS), and mineralization lag time (MLt)
- Changes from baseline in parameters of bone mineralization including mineral apposition rate (MAR), mineralizing surface (MS/BS), bone formation rate (BFR), and others
- Additional PD markers reflecting the status of phosphorus homeostasis and renal function
- Bone remodeling as assessed by bone turnover markers

Exploratory Efficacy Objectives:

- Examine the effect of KRN23 treatment in adults with XLH on pseudofracture healing
- PROs assessing skeletal pain and fatigue

Pharmacokinetic Objective:

Assess the PK of KRN23 throughout the dosing cycle following the first doses and at steady state

Safety Objective:

Establish the safety and tolerability profile of KRN23 in the treatment of adults with XLH including AEs, ectopic mineralization risk, cardiovascular effects, and immunogenicity profile

7 INVESTIGATIONAL PLAN

7.1 Overall Study Design and Plan

UX023-CL304 is a Phase 3 open-label, single-arm, multicenter study to establish the effects of KRN23 on bone quality and osteomalacia associated with XLH. Approximately 14 adult subjects with a diagnosis of XLH supported by typical clinical and biochemical features, and who have not received oral phosphate and vitamin D therapy in the past two years will be enrolled. To ensure a level of gender balance, at least 3 subjects of each sex will be enrolled. Iliac crest bone biopsies will be performed at baseline and Week 48 (or ET if applicable). Baseline histologic and histomorphometric assessments of the bone biopsy specimens will be performed as each biopsy is completed to assess sample quality and confirm the presence of osteomalacia in at least 8 subjects. If a subject is determined not to have osteomalacia at the time of the initial biopsy, that subject will continue on study but will not undergo the second bone biopsy procedure at Week 48. All other assessments will be completed as scheduled. The goal of the study is to assess changes in bone quality. Histologic and histomorphometric evaluation of iliac crest bone biopsies will be supported by changes in serum phosphorus and biochemical markers of bone turnover and additional PD markers associated with FGF23-mediated processes. Pseudofractures and PROs will provide additional information on KRN23 efficacy. Safety, immunogenicity, and PK of KRN23 will also be evaluated.

KRN23 will be administered via SC injections monthly (Q4W, 28 days) for 48 weeks. Subjects will then continue into an additional 48-week Treatment Extension Period I (until Week 96). For subjects at study sites outside of the United States (US), the Week 96 Visit will be their End of Study (EOS) efficacy visit (referred to as EOS I). For subjects at sites in the US, treatment will continue for up to an additional 45 weeks in an open-label Treatment Extension Period II, until end of September 2018, at which time they will have their EOS efficacy visit (referred to as EOS II). (The duration of this period will vary for individual subjects and will be determined by the time from the start of Week 97 through their EOS II Visit). For subjects not immediately continuing KRN23 treatment under commercial use or another mechanism upon completion of study drug treatment or early withdrawal from this study, a Safety Follow-up telephone call (TC) will occur 4 weeks (+5 days) after the subject's final study site visit (ie EOS I [non-US subjects], EOS II [US subjects], or Early Termination [ET] visit) to collect information on any ongoing or new AEs, SAEs, and concomitant medications. If the subject is not continuing KRN23 therapy under commercial use or another mechanism at that time, an additional Safety Follow-up TC will occur at 8 weeks (+ 5 days) after the subject's final study site visit. The end of this study is defined as the last day that the protocol specified assessments (including telephone contact) are conducted for the last subject..

7.2 Discussion of Study Design, Including Choice of Control Group

The study is designed as an open-label, single-arm, multicenter clinical trial intended to establish the beneficial impact of KRN23 on bone quality and strengthen the long-term safety evaluation of KRN23. The study is being conducted in parallel with a pivotal randomized,

double-blind, placebo-controlled Phase 3 efficacy and safety study designed to confirm modulation of phosphate homeostasis and establish the impact of KRN23 treatment on patient-reported outcomes and functional performance.

This Phase 3 study will be conducted in subjects most likely to benefit from treatment: adult XLH patients with osteomalacia and pain. The sample size and study population are intended to characterize KRN23 efficacy as related to bone quality following up to one year of treatment in adult XLH patients.

The protocol consists of multiple study stages, a 48-week Open-Label Treatment Period (Weeks 0-48) and an additional 48-week Treatment Extension Period I (Weeks 49-96). In the US, the study will also include a Treatment Extension Period II (up to approximately 45 weeks). Case reports in XLH and nutritional osteomalacia suggest that healing of osteomalacia and resolution of associated symptoms (e.g., pain) are likely to occur within 3 to 6 months of restoration of phosphorus homeostasis (Kang et al. 2014), (Shikino et al. 2014), (Chalmers et al. 1967). Therefore, the 12-month duration of the initial Open-Label Treatment Period enables a rigorous assessment of improvements in skeletal health associated with phosphate control.

Treatment with oral phosphate and vitamin D metabolites typically stops in adolescence and many adults experience long-term complications from the treatment, such as hyperparathyroidism and nephrocalcinosis. No consensus exists regarding treatment of adult patients with XLH, but symptomatic patients—those with bone or joint pain and/or pseudofractures—may be treated with oral phosphate and vitamin D replacement therapy (Linglart et al. 2014). However, a double-blind, active-comparator design would be very difficult given the differences in the method of administration (oral vs. SC injection for KRN23) and the individualized nature of treatment (Linglart et al. 2014), (Carpenter et al. 2011). Furthermore, bone biopsy assessments and biochemical markers of bone turnover are objective, quantitative measures, which will not be impacted by the open-label design of the study.

KRN23 treatment normalizes serum phosphorous levels, directly impacting the hypophosphatemia and consequent osteomalacia that is the hallmark of XLH, and the cause of the bone abnormalities that result in the clinical signs and symptoms of the disease. The primary efficacy variable reflects reduction in osteoid volume after 48 weeks of treatment. Secondary variables also address the final goals of therapy in adults with XLH, which are to restore skeletal health and thereby reduce the associated pain, fatigue, and fracture risk.

7.3 Selection of Study Population

The study will be conducted in approximately 14 adult subjects with XLH (aged 18 – 65 years) including at least 8 subjects with a confirmed diagnosis of osteomalacia. To ensure a level of gender balance, at least 3 subjects of each sex will be enrolled. The inclusion criteria are structured to enroll subjects with a confirmed diagnosis of XLH,

presenting with characteristic biochemical and clinical features, including reduced serum phosphorus levels, osteomalacia, and pain.

The Sponsor has taken reasonable measures to ensure the protection and safety of this population. Older patients (> 65 years) will be excluded due to frequent comorbidities that may make interpretation of the study results more difficult. Patients with evidence of hyperparathyroidism indicated by increased serum calcium and iPTH, and/or use of calcimimetics will also be excluded.

7.3.1 Inclusion Criteria

Individuals eligible to participate in this study must meet all of the following criteria:

1) Male or female, aged 18 - 65 years, inclusive
2) Diagnosis of XLH supported by classic clinical features of adult XLH (such as short stature or bowed legs), and at least ONE of the following at Screening:
    - Documented *PHEX* mutation in either the patient or in a directly related family member with appropriate X-linked inheritance
    - Serum iFGF23 level > 30 pg/mL by Kainos assay
3) Biochemical findings consistent with XLH based on overnight fasting (min. 8 hours):
    - Serum phosphorus < 2.5 mg/dL at Screening
    - TmP/GFR < 2.5 mg/dL at Screening
4) Presence of skeletal pain attributed to XLH/osteomalacia, as defined by a score of ≥ 4 on the Brief Pain Inventory question 3 (BPI-Q3, Worst Pain) at Screening. (Skeletal pain that, in the opinion of the investigator, is attributed solely to causes other than XLH/osteomalacia—for example, back pain or joint pain in the presence of severe osteoarthritis by radiograph in that anatomical location—in the absence of any skeletal pain likely attributed to XLH/osteomalacia should not be considered for eligibility.)
5) Estimated glomerular filtration rate (eGFR) ≥ 60 mL/min (using the Chronic Kidney Disease Epidemiology Collaboration [CKD-EPI] equation) or eGFR of 45 to <60 mL/min at Screening with confirmation that the renal insufficiency is not due to nephrocalcinosis
6) Provide written informed consent after the nature of the study has been explained, and prior to any research-related procedures. If the subject in a minor, provide written assent and have a legally authorized representative willing and able to provide written informed consent, after the nature of the study has been explained, and prior to any research-related procedures

7) Willing to provide access to prior medical records for the collection of biochemical and radiographic data and disease history

8) Females of child-bearing potential must have a negative urine pregnancy test at Screening and Baseline and be willing to have additional pregnancy tests during the study. Females considered not to be of childbearing potential include those who have not experienced menarche, are post-menopausal (defined as having no menses for at least 12 months without an alternative medical cause) or are permanently sterile due to total hysterectomy, bilateral salpingectomy, or bilateral oophorectomy.

9) Participants of child-bearing potential or fertile males with partners of child-bearing potential who are sexually active must consent to use a highly effective method of contraception as determined by the site Investigator from the period following the signing of the informed consent through the final Safety Follow-up TC (as defined in Section 7.4.3.1)

10) Must, in the opinion of the investigator, be willing and able to complete all aspects of the study, adhere to the study visit schedule and comply with the assessments

7.3.2 Exclusion Criteria

Individuals who meet any of the following exclusion criteria will not be eligible to participate in the study:

1) Use of any pharmacologic vitamin D metabolite or analog (e.g. calcitriol, doxercalciferol, and paricalcitol) within the 2 years prior to Screening

2) Use of oral phosphate within the 2 years prior to Screening

3) Use of aluminum hydroxide antacids, acetazolamides, and thiazides within 7 days prior to Screening

4) Use of bisphosphonates in the 2 years prior to Screening

5) Use of denosumab in the 6 months prior to Screening

6) Use of teriparatide in the 2 months prior to Screening

7) Chronic use of systemic corticosteroids defined as more than 10 days in the 2 months prior to Screening

8) Corrected serum calcium level ≥ 10.8 mg/dL (2.7 mmol/L) at Screening

9) Serum iPTH ≥ 2.5 times the upper limit of normal (ULN) at Screening

10) Use of medication to suppress PTH (cinacalcet for example) within 60 days prior to Screening

11) Prothrombin time/Partial thromboplastin time (PT/PTT) outside the normal range at Screening

12) Evidence of any disease or use of anticoagulant medication (such as warfarin, heparin, direct thrombin inhibitors, or xabans that, in the opinion of the investigator, cannot be discontinued) that may increase the risk of bleeding during the biopsy procedure

13) Pregnant or breastfeeding at Screening or planning to become pregnant (self or partner) at any time during the study

14) Unable or unwilling to withhold prohibited medications throughout the study

15) Documented dependence on narcotics

16) Use of KRN23, or any other therapeutic monoclonal antibody within 90 days prior to Screening

17) Use of investigational product or investigational medical device within 30 days prior to Screening, or requirement for any investigational agent prior to completion of all scheduled study assessments.

    OR, in Japan, use of any investigational product or investigational medical device within 4 months prior to Screening, or requirement for any investigational agent prior to completion of all scheduled study assessments.

18) Presence or history of any hypersensitivity, allergic or anaphylactic reactions to any monoclonal antibody or KRN23 excipients that, in the judgment of the investigator, places the subject at increased risk for adverse effects

19) History of allergic reaction or adverse reactions to tetracycline or demeclocycline

20) Prior history of positive test for human immunodeficiency virus antibody, hepatitis B surface antigen, and/or hepatitis C antibody

21) History of recurrent infection (other than dental abscesses, which are known to be associated with XLH) or predisposition to infection, or of known immunodeficiency

22) Presence of malignant neoplasm (except basal cell carcinoma)

23) Presence of a concurrent disease or condition that would interfere with study participation or affect safety

24) Presence or history of any condition that, in the view of the investigator, places the subject at high risk of poor treatment compliance or of not completing the study

7.3.3 Removal of Subjects from Therapy or Assessment

In accordance with the Declaration of Helsinki, subjects have the right to withdraw from the study at any time for any reason. The investigator and Ultragenyx also have the right to remove subjects from the study. Ultragenyx must be notified of all subject withdrawals as soon as possible. Ultragenyx also reserves the right to discontinue the study at any time for either clinical or administrative reasons and to discontinue participation of an individual subject or investigator due to poor enrollment or noncompliance, as applicable.

Subjects may be removed from the study for the following reasons:

- Occurrence of an unacceptable AE
- An illness that, in the judgment of the investigator or Ultragenyx, might place the subject at risk or invalidate the study
- Pregnancy in subject
- At the request of the subject, investigator, or Ultragenyx, for administrative or other reasons
- Protocol deviation or noncompliance

If the reason for removal of a subject from the study is an AE, the AE and any related test or procedure results will be recorded in the source documents and transcribed onto the Case Report Form (CRF). Each clinically significant abnormal laboratory value or other clinically meaningful abnormality should be followed until the abnormality resolves or until a decision is made that it is not likely to resolve. If such abnormalities do not return to normal within 30 days after the last dose given, their etiology should be identified and Ultragenyx should be notified. All unscheduled tests must be reported to Ultragenyx immediately.

If a subject discontinues from the study prematurely, every reasonable effort should be made to perform the Early Termination Visit procedures within four weeks of discontinuation. Subjects who discontinue due to SAE will be monitored for safety through the final Safety Follow-up TC (as defined in Section 7.4.3.1).

7.3.3.1 Stopping Rules

No formal statistical stopping criteria will be applied for this study. A Data Monitoring Committee (DMC) will be constituted for Studies UX023-CL303 and UX023-CL304 and will act in an advisory capacity to monitor the safety of KRN23 on a routine basisuntil the end of the Treatment Period (Week 48) (Section 7.6.7). The DMC may provide advice to Ultragenyx to aid in the determination of whether study enrollment should be paused or if the study should be stopped. If the Sponsor deems it appropriate to restart the trial following an internal safety review, this will be done only following approval by Regulatory Authorities.

During Treatment Extension Periods I and II, safety data will be reviewed by the Ultragenyx Study Safety Review Team (SSRT) as described in Section 8.5.4.6.

Individual subjects who experience any unexpected and possibly, probably, or definitely drug-related SAEs (Section 8.5.3) that represent a change in the nature or an increase in frequency of the serious event from their prior medical history will be assessed as to whether the subject will continue on the study.

Individual subjects will be monitored for ectopic mineralization (Section 7.5.3). If new or clinically significant worsening in mineralization is considered clinically meaningful by the investigator and/or sponsor and related to study drug, the subject will be discontinued from the study.

Regulatory Authorities, as well as the Institutional Review Board (IRB)/Ethics Committee (EC) will be informed should unexpected and possibly, probably, or definitely drug-related SAEs occur. A full clinical evaluation of the event will be performed in order to make a decision regarding what actions to take, including whether to recommend stopping the study. Regulatory Authorities, as well as IRBs/ECs, will be informed if the study is paused or stopped.

7.4 Treatments

The study drug is manufactured, packaged, and labeled according to Good Manufacturing Practice (GMP) regulations. The Investigational Product should be securely stored under conditions indicated in the IB.

7.4.1 Investigational Product

KRN23 is a sterile, clear, colorless, and preservative-free solution supplied in single-use 5-mL vials containing 1 mL of KRN23 at a concentration of 30 mg/mL. KRN23 will be administered without dilution. All subjects will receive 1.0 mg/kg KRN23 monthly (Q4W, 28 days), rounded to the nearest 10 mg. The amount of drug administered will be calculated based on baseline weight and a 1.0 mg/kg KRN23 dose level (rounded to the nearest 10 mg) up to a maximum dose of 90 mg. The dose will remain fixed for the duration of the study, provided serum phosphorous levels do not exceed 5.0 mg/dL (1.61 mmol/L) at any time or 4.5 mg/dL (1.45 mmol/L) on two occasions. The dose will be recalculated if body weight changes by more than 20% from the baseline measurement.

Subjects will receive study drug via SC injection to the abdomen, upper arms, or thighs; the injection site will be rotated with each injection. No more than 1.5 mL may be administered to a single injection site. If the dose requires more than 1.5 mL, multiple injections must be administered, each at a different injection site.

If serum phosphorus increases above 5.0 mg/dL (1.61 mmol/L) at any time the actual dose will be decreased by half. If serum phosphorous increases above the ULN (4.5 mg/dL; 1.45 mmol/L) but does not exceed 5.0 mg/dL (1.61 mmol/L), the dose will be adjusted only

if a second serum phosphorus result exceeds the ULN. Following a downward dose adjustment, the investigator together with the medical monitor should determine how and when to dose titrate up. Unscheduled serum phosphorus assessments may be necessary. Based on the totality of the data from studies INT-001 and INT-002 over a period of 16 months in which most subjects were treated with 1.0 mg/kg KRN23 and no subject experienced an elevation of serum phosphorus that approached the 4.5 mg/dL (1.45 mmol/L) threshold, it is considered unlikely that dose adjustment will be necessary.

Refer to the Study Reference Manual for additional information on dose adjustments.

7.4.2 Reference Therapy

The study is a single-arm, open-label study. All subjects will be on active treatment; no reference therapy or placebo will be administered.

7.4.3 Selection of Doses and Study Duration

The goal of therapy in adults with XLH is to increase serum phosphorus sufficiently to allow the bone to fully mineralize, and thereby improve bone physiology, structure, and function. The level of serum phosphorus needed to restore bone metabolism homeostasis and mineralization is not precisely known, but there is a general agreement that increasing serum phosphorus above the lower limit of normal (LLN) is an appropriate strategy. Another key consideration of the therapeutic goal is related to the safety concerns associated with ectopic mineralization, in particular nephrocalcinosis. The risk of nephrocalcinosis with the current oral phosphorus and active vitamin D therapy increases with higher doses, and is believed to be associated with exacerbated oral phosphate wasting in the urine, and secondary hypercalciuria (Goodyer et al. 1987), (Verge et al. 1991), (Sabbagh et al. 2008). Although mechanistically KRN23 does not increase urinary phosphorus or calcium, the dosing strategy will be conservative, aiming to maintain serum phosphorus above the LLN for the majority of a given dose interval without causing hyperphosphatemia.

The Phase 3 dose is based on PK, PD, clinical, and safety data obtained in previous studies (KRN23-US-02, KRN23-INT-001 and KRN23-INT-002). These studies indicate normalization of serum phosphorus levels occurs with SC KRN23 doses ranging from 0.3 to 1.0 mg/dL Q4W. The safety data showed repeated monthly doses up to 1.0 mg/kg were well tolerated by adult subjects with XLH.

Data from KRN23-INT-002, the longest clinical study to date, is the primary basis of the dosing strategy. All subjects in the study responded to KRN23 at doses of 0.3, 0.6, and 1.0 mg/kg by increasing serum phosphorus. After the initial dose-titration period, the majority of subjects (60% to 74%) stabilized on an optimal dose of 1.0 mg/kg KRN23 and achieved serum phosphorus levels within the normal range. The maximum increase in serum phosphorus following KRN23 administration was approximately 1 mg/dL from baseline. No hyperphosphatemia (>4.5 mg/dL; 1.45 mmol/L) was detected at any time point. Increases in serum phosphorus persisted through much of each treatment interval and

throughout the 12 months of treatment; however, serum phosphorus decreased towards the end of each dose interval as KRN23 concentrations waned. Importantly, KRN23 administration increased serum phosphorus without negatively affecting other parameters such as iPTH and calcium.

Based on these data, a KRN23 dose of 1.0 mg/kg (rounded to the nearest 10 mg) administered Q4W by the SC route was selected as the dose and regimen expected to be therapeutic while maintaining a reasonable safety margin.

7.4.3.1 Study Duration

The planned duration of treatment in this study is 96 weeks for subjects at sites outside of the US or up to approximately 141 weeks, until end of September 2018, for subjects at sites in the US, or until one of the following occurs: the subject withdraws consent, the subject is discontinued from the study at the discretion of the investigator or Ultragenyx, or the study is terminated. The length of the study is intended to define whether KRN23 is effective at reducing excess osteoid after 48 weeks. Subjects who complete the Open-Label Treatment Period will continue to receive KRN23 for an additional 48 weeks during Treatment Extension Period I. Subjects at study sites outside the US will have their EOS efficacy visit (ie, EOS I) at the end of this period. Subjects at US sites will continue KRN23 treatment for up to an additional approximately 45 weeks in Treatment Extension Period II. The duration of Treatment Extension Period II may vary for individual subjects. For each subject who participates in this period, the study site will schedule the EOS efficacy visit (ie, EOS II) to take place before 30 September 2018. The duration of the period will be determined by the time from the start of Week 97 through the EOS II Visit.

For subjects not immediately continuing KRN23 treatment under commercial use or another mechanism upon completion of study drug treatment or early withdrawal from this study, a Safety Follow-up TC will occur 4 weeks (+ 5 days) after their final study site visit (ie, EOS 1 [non-US subjects], EOS II [US subjects], or ET Visit) to collect information on whether KRN23 treatment has been started through another mechanism and, if not, any ongoing or new AEs, SAEs, or concomitant medications. If the subject is not continuing KRN23 therapy under commercial use or another mechanism at that time, an additional Safety Follow-up TC will occur 8 weeks (+ 5 days) after the subject’s final study site visit to collect information on whether KRN23 treatment has been started through another mechanism and, if not, any ongoing or new AEs, SAEs, or concomitant medications.

The end of the study is defined as the last day that protocol-specified assessments (including telephone contact) are conducted for the last subject. The maximum planned study duration (treatment plus safety follow-up) is approximately 149 weeks.

7.4.4 Method of Assigning Subjects to Treatment Groups

Subjects will be enrolled in the study and sequentially assigned an identification number. All subjects will receive active treatment (KRN23); there is only one treatment group in the study.

7.4.5 Blinding

The study drug will be administered open-label; no study participants will be blinded to study treatment.

7.4.6 Treatment Compliance

Trained personnel will administer study drug by SC injection at the investigational site or during home health (HH) visits as indicated in the Schedule of Events for that period (Table 2.1, Table 2.2, Table 2.3). Each administration of study drug will be recorded on the CRF. If a subject does not receive a dose within 21 days of a scheduled dose, that dose should be skipped and the next dose will be administered at the next scheduled dosing visit. Doses should be administered no fewer than 14 days apart.

7.5 Study Procedures and Assessments

Potential subjects will come in to the site to sign informed consent and complete the initial Screening. Subjects will be contacted by telephone within 1 week of the Screening visit to communicate available test results related to eligibility. For patients who do not meet eligibility on the basis of the iFGF23 assay, *PHEX* mutation analysis may be performed before baseline. If eligible, tetracycline HCl (or demeclocycline) will be provided to the subject with instructions for administration. The bone biopsy should be performed 5 days after the last dosing day for tetracycline (or demeclocycline): The first dose should be given on days -20, -19, and -18, and the second dose given on days -8, -7, and -6. Similar telephone contacts will be placed to provide instructions for tetracycline (or demeclocycline) labeling prior to the Week 48 or, if eligible, the Early Termination (ET) visits.

All Screening/Baseline assessments and inclusion/exclusion criteria must be satisfied prior to dosing. The Baseline visit should occur no more than 31 days following the Screening visit. If the Baseline visit (BL) occurs more than 31 days following the Screening Visit, medical history and pregnancy status should be re-assessed. The Baseline, Week 48, and Week 96 (or ET) assessments may be completed in any reasonable order (except where indicated) to allow for flexibility in scheduling. Renal ultrasound, ECHO, ECG, and skeletal survey/ targeted x-rays may be performed within ± 3 days of indicated clinic visit to accommodate scheduling availability. The bone biopsy must be completed on a day with no other procedures, and prior to dosing on Day 0.

During the Open-Label Treatment Period (Weeks 0 – 48), Treatment Extension Period I (Weeks 49 – 96), and Treatment Extension Period II (US sites only, Weeks 97 up to Weeks 141), subjects will return to the clinic and/or have HH visits as indicated (± 5 days).

Visits designated HH may also be conducted at the investigational site depending on the preference of the subject and proximity of the subject to the clinic. For subjects at sites outside of the US, the Week 96 Visit will be their EOS efficacy visit (ie, EOS I). For sites in the US, at the end of Treatment Extension Period II (ie, final study visit before 30 September 2018), the subjects will have their EOS efficacy visit (ie, EOS II).

For subjects not immediately continuing KRN23 treatment under commercial use or another mechanism upon completion of study drug treatment or early withdrawal from this study, a Safety Follow-up TC will occur 4 weeks (+5 days) after their final study site visit (ie, EOS 1 [non-US subjects], EOS II [US subjects], or ET Visit) to collect information on whether KRN23 treatment has been started through another mechanism and, if not, any ongoing or new AEs, SAEs, or concomitant medications. If the subject is not continuing KRN23 therapy under commercial use or another mechanism at that time, then site personnel will initiate a subsequent Safety Follow-up TC 8 weeks (+ 5 days) after the subject's final study site visit to collect information on whether KRN23 treatment has been started through another mechanism and, if not, any ongoing or new AEs, SAEs, or concomitant medications.

The schedule of visits and assessments conducted during Screening and Baseline, and the Open-Label Treatment Period are provided in Table 2.1 and Table 2.2. The schedule of visits and assessments conducted during the Treatment Extension Periods I and II are provided in Table 2.3 and Table 2.4. Refer to the Study Reference Manual for additional details and a recommended schedule and order of specific assessments.

7.5.1 Efficacy Measures

Efficacy will be evaluated by changes in histomorphometric indices of osteomalacia, supported by PD markers of phosphate homeostasis and renal function, and patient-reported changes in pain and fatigue. Results from baseline assessments will be compared with those of post-treatment assessments, with efficacy conclusions based on change from baseline.

The efficacy variables in this study: OV/BV, O.Th, OS/BS, and MLt, will provide a histomorphometric indicator of KRN23 action. The primary efficacy endpoint will be the reduction in osteoid volume (OV/BV) based on analysis of iliac crest bone biopsies after 48 weeks of KRN23 treatment. Osteoid volume (measured as a percentage of total bone volume) is the most consistent histomorphometric parameter to quantify the amount of osteoid tissue accumulation, which is the hallmark of osteomalacia. The primary efficacy measure will be supported by the key secondary variable, serum phosphorus levels, along with multiple secondary and exploratory variables, including additional histomorphometric parameters, biochemical markers, and PROs. Secondary and exploratory endpoints and associated analyses are described in Section 7.6.4. The following section describes the assessments that will be performed throughout the study to derive primary, key secondary, secondary, and exploratory variables.

7.5.1.1 Iliac Crest Bone Biopsy

In adulthood, XLH progresses into a disease of persistent and often clinically significant osteomalacia as a result of chronic low serum phosphorus, causing bone and joint pain and pseudofractures in a large proportion of patients (Reid et al. 1989). Preclinical and clinical data suggest static and dynamic parameters of osteoid accumulation improve significantly following anti-FGF23 treatment in the Hyp mouse model (Aono et al. 2009), or 6-9 months of supplementation therapy in adults with XLH (Sullivan et al. 1992), (Harrell et al. 1985). Therefore, iliac crest bone biopsies will be obtained to assess OV/BV as the primary outcome measure in this study; by performing the procedure before and after treatment with KRN23, each subject will serve as an individual control.

A bone biopsy of the iliac crest will be performed to assess OV/BV, O.Th, OS/BS, MLt as well as other parameters. Subjects will receive two courses of tetracycline (or demeclocycline) label prior to biopsy (see Section 7.5) associated with the Baseline and Week 48 (or Early Termination if it occurs between the Week 24 and Week 48) visits. Bone biopsy of the trans-iliac crest will be performed by a physician trained and experienced in the procedure. Baseline bone biopsies will be qualitatively analyzed in real time to assess the presence of osteomalacia. If a subject is determined not to have osteomalacia at the time of the initial biopsy, that subject will continue on study but will not undergo the second bone biopsy at Week 48/ET.

Primary and secondary efficacy endpoints derived from these assessments are provided in Section 7.6. Refer to the Transiliac Crest Bone Biopsy Manual for detailed instructions on bone labeling, biopsy, sample processing, and histomorphometry.

7.5.1.2 Biochemical Markers

Osteomalacia is a hallmark of XLH in adults and results from the underlying mineralization defect due to hypophosphatemia. Normalization of bone turnover with improved mineralization would be expected to prevent pseudofractures or accelerate their healing. Biochemical markers of bone formation will be evaluated as an indicator of changes in bone metabolism in response to KRN23 treatment. Skeletal health parameters will be used to establish the improvement in biomarkers and modification of pathological features of bone disease in XLH.

7.5.1.2.1 Serum Phosphorus

KRN23 binds to and inhibits FGF23. As a phosphaturic hormone, FGF23 plays an important role as a specific regulator of serum phosphorus levels. Normalization of serum phosphorus directly bears on the hypophosphatemia and consequent osteomalacia that is the hallmark of XLH and the cause of the bone abnormalities that result in the clinical signs and symptoms of the disease. The key secondary efficacy variable in this study, serum phosphorus levels at the mid-point of the dosing cycle, will provide a biochemical indicator of KRN23 action across the dosing interval.

7.5.1.2.2 Biochemical Markers of Bone Turnover

Biochemical markers of bone turnover in serum, including procollagen type 1 N-propeptide (P1NP), carboxy-terminal cross-linked telopeptide of type I collagen (CTx), and bone-specific alkaline phosphatase (BALP) will be evaluated over time as an indicator of changes in bone metabolism in response to KRN23 treatment. Serum samples will be collected at the Baseline (Day -2), Week 12, Week 24, Week 48, Week 72, and Week 96/EOS I visits and, if applicable, EOS II (or Early Termination) as indicated in the Schedules of Events (Table 2.1, Table 2.2, Table 2.3, and Table 2.4).

7.5.1.2.3 Additional Pharmacodynamic Markers

To demonstrate the positive impact of KRN23 on bone metabolism and phosphate homeostasis, additional PD markers will be assessed, including serum calcium, 1,25(OH)$_2$D, urinary phosphorus, ratio of renal tubular maximum reabsorption rate of phosphate to glomerular filtration rate (TmP/GFR), and tubular reabsorption of phosphate (TRP). These measures will directly reflect the mechanism by which KRN23 restores phosphorus homeostasis by inhibiting excess FGF23.

Serum samples will be obtained following a minimum overnight fasting time of 8 hours and prior to study drug administration (if applicable). Samples should be obtained in the morning at approximately the same time for each visit due to known fluctuations in serum phosphorous levels associated with circadian rhythm. The time of sample collection will be noted on the CRF.

Two-hour fasting urine collection is required to assess phosphate reabsorption (TmP/GFR and TRP) based on simultaneous urine and blood creatinine and phosphorus concentrations. Both 2-hr and 24-hr urine will be used for measurements of urinary phosphorus, creatinine and calcium. The duration of fasting time for all PD parameters will be recorded on the CRF.

PD markers will be assessed as specified in the Schedule of Events (Table 2.1, Table 2.2, Table 2.3, and Table 2.4). When possible, samples will be used to assess both PD and safety parameters to minimize blood draw requirements and patient burden (Section 7.5.3.8).

7.5.1.3 Patient Reported Outcomes

PROs will support the effect of KRN23 on patient benefit and clinical outcomes in this population. Adult patients with XLH experience a number of symptoms including bone and joint pain due to the underlying osteomalacia and joint pain related to osteoarthritis that often develops in adults with XLH as a consequence of the ongoing loading of joints that became misaligned during childhood due to XLH rickets and bowing. The BPI and BFI will be used to assess the effects of KRN23 treatment on patient-reported and quality of life outcomes. PROs will be administered in order of presentation below at Baseline (Week 0) and Weeks 12, 24, 36, 48, 72, and 96/EOS I and, if applicable, Q24W during Treatment Extension Period II and at EOS II, as indicated in the Schedules of Events (Table 2.1,

Table 2.2, Table 2.3, and Table 2.4). PROs will not be administered if a subject terminates from the study early (Early Termination).

7.5.1.3.1 Brief Pain Inventory

The short-form BPI is an 11-question, self-reported, pain-specific PRO with a recall period of 24 hours that may allow a detailed characterization of the pain experienced by patients with XLH. Initially developed to assess pain in cancer patients, the instrument has since been validated to characterize non-malignant pain in patients with arthritis or low back pain (Keller et al. 2004). The BPI evaluates the condition of all pain over the previous 24 hours. Two dimensions are measured: pain severity (worst, least, average, and now) and the impact of pain on functioning (pain interference with general activity, walking, work, mood, enjoyment of life, relations with others, and sleep). The BPI question 3 (BPI Q3; Worst Pain) will be administered at Screening to assess eligibility. The complete short-form BPI will be administered at all remaining indicated visits.

7.5.1.3.2 Brief Fatigue Inventory

The BFI is a self-reported questionnaire consisting of 9 items related to fatigue that are rated on a 0 to 10 numerical rating scale with a recall period of 24 hours. As with the BPI, two dimensions are measured: fatigue and the interference of fatigue on daily life (activity, mood, walking ability, work, relations with others, and enjoyment of life). The BFI was developed to evaluate severity of fatigue due to cancer and cancer treatment (Mendoza et al. 1999) and has been used in musculoskeletal conditions including osteoarthritis and rheumatoid arthritis (Murphy et al. 2010), (Murphy et al. 2013), (Wolfe 2004). The complete BFI will be administered at Screening and at all indicated visits.

7.5.1.4 Targeted Radiography and Skeletal Survey

A radiographic skeletal survey will be conducted at baseline to assess healing or resolution of current pseudofractures, determine areas of osteomalacia and enthesopathy, and identify the number of active fractures/pseudofractures. Standard radiographs will be obtained of the chest, lateral spine, right and left hand/wrist, right and left humerus, right and left radius/ulna, right and left femur/pelvis, right and left tibia/fibula, and right and left foot.

During the 48-week long Open-Label Treatment Period, targeted radiography at locations identified by the skeletal survey will be taken at Weeks 12, 24, 36, and 48 to monitor frequency and healing of pseudofractures and/or fractures. During the Treatment Extension Periods I and II (if applicable), targeted radiographs will only be taken at clinic visits following newly diagnosed fractures.

Post-baseline radiographs will be compared to baseline radiographs using a pre-defined list of abnormalities by a trained central reader who is blinded to subject data. Existing and new pseudofractures will be graded as not healed, partially healed, or fully healed.

7.5.2 Drug Concentration Measurements

PK samples will be obtained from all subjects throughout the study at indicated time points representing the peak and trough exposure levels (Table 2.1, Table 2.2, Table 2.3, and Table 2.4). The actual dates and times of study drug administration and sample collection will be documented in the CRF. The concentration of KRN23 in human serum will be determined using a validated electrochemiluminescent assay (ECLA) method.

7.5.3 Safety Measures & General Assessments

General assessments include medical history, height, and demographics. *PHEX* mutation analysis will be performed for all qualified subjects even if they or a family member has a confirmed *PHEX* mutation. If the initial result for PHEX mutation analysis is negative or inconclusive (i.e., No Mutation, Likely Benign, Variant of Uncertain Significance, or Possibly Pathogenic) and informed consent is provided by the subject, additional genetic testing will be performed to assess mutations in other genes associated with phenotypes overlapping with XLH. This testing will include genes for Autosomal Dominant Hypophosphatemic Rickets (*FGF23*), Autosomal Recessive Hypophosphatemic Rickets (*DMP1*, *ENPP1*), X-Linked Recessive Hypophosphatemic Rickets (*CLCN5*), Raine Syndrome (*FAM20C*), and Hereditary Hypophosphatemic Rickets with Hypercalciuria (*SLC34A3*). The investigator will communicate any additional genetic testing results to the subject.

Safety will be evaluated by the incidence, frequency and severity of AEs and SAEs, including clinically significant changes from baseline to scheduled time points in vital signs, weight, physical examination, eGFR, clinical laboratory evaluations (including additional KRN23/XLH biochemical parameters of interest), and concomitant medications. Ectopic mineralization safety assessments include renal ultrasound, ECHO and ECG.
The development of anti-KRN23 antibodies (anti-drug antibodies [ADA]) will also be assessed. Pregnancy testing will be conducted in females with child-bearing potential.

7.5.3.1 Medical History

General medical information includes subject demographics (date of birth, ethnicity, and sex) and a history of major medical illnesses, diagnoses, and surgeries. The review will also include a detailed assessment of symptoms and conditions associated with XLH and treatment.

Subjects must be willing to provide access to prior medical records for the collection of biochemical and radiographic data, as well as disease history. The specific diagnosis of XLH will be recorded, along with date of onset, clinical presentation, and date and method of diagnosis. Any available family history of XLH will be noted, including any available previous *PHEX* mutation analysis results for the subject or relevant family members with appropriate X-linked inheritance pattern.

XLH treatment history and relevant concomitant medications will be recorded (start date, stop date, dose, dose regimen). Previous treatments may include calcitriol and oral phosphate, calcimimetics or other adjunctive therapy. Medications include investigational, prescription, over-the-counter, herbal and nutritional supplements. Any relevant concomitant therapy, including physical/occupational therapy will be recorded. Use of pain medications will also be recorded. Refer to Section 7.5.3.10.1 for prohibited medications during the study.

7.5.3.2 Vital Signs

Vital signs will include seated systolic blood pressure and diastolic blood pressure measured in millimeters of mercury (mm Hg; 2 measurements separated by 15 minutes), heart rate in beats per minute, respiration rate in breaths per minute, and temperature in degrees Celsius (°C). Vital signs measurements will be obtained at specified visits (Table 2.1, Table 2.2, Table 2.3, and Table 2.4) before any additional assessments are completed.

7.5.3.3 Weight and Height

Weight will be obtained using a scale and recorded in kilograms at specified visits (Table 2.1, Table 2.2, Table 2.3, and Table 2.4). The measurement obtained at the Baseline visit will be used to calculate the appropriate dose of study drug to be administered on a mg/kg basis throughout the duration of the study, unless weight changes by more than 20%, in which case the dose of study drug should be recalculated according to the new body weight.

Height (in meters) will be obtained using a stadiometer (without shoes) at the Screening Visit only.

7.5.3.4 Physical Examination

Complete physical examinations will be performed at specified visits (Table 2.1, Table 2.2, Table 2.3, and Table 2.4). Physical examinations will include assessments of general appearance; head, eyes, ears, nose, and throat; the cardiovascular, dermatologic, lymphatic, respiratory, gastrointestinal, genitourinary, musculoskeletal, and neurologic systems.

The genitourinary exam scope should be non-invasive and as per age-appropriate standard of care, at the Investigator's discretion based on clinical judgement and/or safety need. If the Investigator determines there is no clinical indication for a genitourinary exam, it is not necessary to perform.

7.5.3.5 Renal Ultrasound and Glomerular Filtration Rate

Renal ultrasounds will be conducted at Screening and Weeks 24, 48, 72, and 96/EOS I and, if applicable, EOS II (or Early Termination), as indicated in the Schedules of Events (Table 2.1, Table 2.2, Table 2.3, and Table 2.4). The ultrasound will be interpreted by qualified personnel at the investigational site for purposes of inclusion criteria.

Results obtained at Screening will serve as baseline data. Baseline and all post-treatment renal ultrasounds will be evaluated by a trained central reader blinded to subject data to evaluate changes in calcifications and all other renal abnormalities from baseline (i.e. screening assessment). Ultrasonographic findings of nephrocalcinosis will be graded on a 5-point scale (Patriquin et al. 1986).

The eGFR will be calculated using the Chronic Kidney Disease Epidemiology Collaboration (CKD-EPI) equation (Levey et al. 2009) for determining creatinine clearance. Results obtained at screening will serve as baseline data.

7.5.3.6 Echocardiogram

ECHO will be performed Weeks 24, 48, 72, and 96/EOS I and, if applicable, EOS II (or Early Termination if not performed within 3 months of termination) as indicated in the Schedules of Events (Table 2.1, Table 2.2, Table 2.3, and Table 2.4). The goal is twofold: 1) assess for evidence of ectopic mineralization in the heart and aorta, and 2) evaluate for signs of LVH or cardiac dysfunction. Additional tests may be performed if any abnormalities are detected or if medically indicated. ECHO administration procedures will be standardized and results will be centrally read.

7.5.3.7 Electrocardiogram

A standardized 12-lead ECG will measure PR, QRS, QT, and QTc at Baseline and Weeks 24, 48, 72, and 96/EOS I and, if applicable, EOS II (or Early Termination) as indicated in the Schedules of Events (Table 2.1, Table 2.2, Table 2.3, and Table 2.4). The goal is to evaluate both for LVH changes, as well as for changes in conductivity and intervals. ECG administration procedures will be standardized and results will be read centrally by qualified personnel blinded to subject data. The ECG results will be assessed for any clinically significant abnormality or relevant changes from baseline.

7.5.3.8 Clinical Laboratory Tests for Safety

A comprehensive serum metabolic panel (Chem-20), complete blood count, and urinalysis will be used as routine screens to assess KRN23 safety. Screening and Week 48 Day 1 hematology will include standard tests for risk of bleeding. Certain analytes (i.e., serum phosphorus) in the routine Chem-20 panel are also designated as PD/efficacy parameters in this study (Section 7.5.1.2.3). Additional KRN23/XLH biochemical parameters of interest include serum 1,25$(OH)_2$D, calcium, creatinine, and iPTH; and urinary phosphorus, calcium and creatinine.

Blood and urine samples will be collected at Screening, Baseline, and regular intervals throughout the study as indicated in the Schedule of Events (Table 2.1, Table 2.2, Table 2.3, and Table 2.4). Fasting for a minimum of 8 hours (overnight) is required prior to each blood draw; the duration of fasting will be recorded on the CRF. Twenty-four hour urine collection is required to assess urinary phosphorus:creatinine and calcium:creatinine ratios; urinary phosphorus, a PD parameter, will also be obtained from 24-hour urine samples.

Clinical laboratory parameters to be assessed for safety are provided in Table 7.5.3.8.1. See Study Reference Manual for details on sample collection and processing.

Table 7.5.3.8.1: Clinical Laboratory Assessments for Safety

**Chemistry**	**Hematology**	**Urinalysis**
25(OH) D	Hematocrit	Appearance
1,25(OH)$_2$D	Hemoglobin	Color
Alanine aminotransferase (ALT)	Platelet count	pH
Alkaline phosphatase (ALP)	Red blood cell (RBC) count	Specific gravity
Amylase	White blood cell (WBC) count	Ketones
Aspartate aminotransferase (AST)	Mean corpuscular volume (MCV)	Protein
Bilirubin (direct and total)	Mean corpuscular hemoglobin (MCH)	Glucose
Blood urea nitrogen (BUN)	MCH concentration	
Calcium (total)	Prothrombin time	**24-hour Urine**
Chloride	Partial thromboplastin time	Calcium
Carbon dioxide (CO_2)		Calcium/creatinine ratio
Cholesterol (total)		Creatinine
Creatinine		Phosphorus/creatinine ratio
Gamma-glutamyl transpeptidase (GGT)		Phosphorus
Glucose		**2-hour Urine**
Intact parathyroid hormone (iPTH)		Calcium
Lactate dehydrogenase (LDH)		Creatinine
Lipase		Calcium/creatinine ratio
Phosphorus		Phosphorus
Potassium		TmP/GFR
Protein (albumin and total)		TRP
Sodium		Pregnancy test (if applicable)
Uric acid		
Serum pregnancy test (if applicable)		

Subjects who experience a SAE possibly or probably related to study drug or other AE of concern may, at the discretion of the investigator (and/or medical monitor), have additional blood samples taken for safety laboratory tests.

7.5.3.8.1 Fibroblast Growth Factor 23

Serum FGF23 concentrations will be measured using a validated assay. Blood samples will be collected at specified visits (Table 2.1, Table 2.2, Table 2.3, and Table 2.4) after a minimum overnight fasting time of 8 hours and prior to drug administration (if applicable).

7.5.3.8.2 Anti-KRN23 Antibody Testing

To determine the immunogenicity profile of KRN23 in adults with XLH, blood samples will be obtained for anti-KRN23 antibody (ADA) testing at Day 0/Weeks 0, 4, 24,48, 72, and 96/EOS I and, if applicable, EOS II (or Early Termination), as indicated in the Schedules of Events (Table 2.1, Table 2.2, Table 2.3, and Table 2.4).. PK samples will also be obtained on days of ADA testing to assess potential neutralizing effects if ADA are detected. The formation of anti-KRN23 antibodies in human serum will be determined using a validated ECLA and a 3-tiered strategy: screening, specificity confirmation, and titer assays. If the development of anti-KRN23 antibodies is suspected in a given subject, samples may be obtained at additional time points on a case-by-case basis, if warranted.

7.5.3.9 Pregnancy Testing

Female subjects of childbearing potential will have urine pregnancy tests at the indicated visits (Table 2.1, Table 2.2, Table 2.3, and Table 2.4) throughout the duration of the study. Female subjects with a positive pregnancy test at Screening will not be enrolled in the study.

Additional pregnancy tests will be performed at any visit in which pregnancy status is in question. A serum pregnancy test will be performed in the event of a positive or equivocal urine pregnancy test result, or can be performed if pregnancy test by urine is not feasible. Pregnancy in subject or partner must be reported (Section 8.5.4.5); pregnant subjects will be discontinued from the study.

Experience with KRN23 in pregnant women is limited. The study drug may involve risks to a pregnant female or unborn baby which are currently unknown. Female participants of child-bearing potential must consent to use a highly effective method of contraception as listed below from the period following the signing of the informed consent through the final Safety Follow-up TC (as defined in Section 7.4.3.1). Sexually active male participants with female partners of childbearing potential must consent to use a condom with spermicide or one of the highly effective methods of contraception listed below from the period following the signing of informed consent through the final Safety Follow-up TC (as defined in Section 7.4.3.1).

Highly effective methods of contraception (CTFG 2014) include:

- Combined (estrogen and progestogen containing) hormonal contraception associated with inhibition of ovulation (e.g. oral, intravaginal, transdermal)
- Progestogen-only hormonal contraception associated with inhibition of ovulation (e.g. oral, injectable, implantable)
- Intrauterine device (IUD) or intrauterine hormone-releasing system (IUS)
- Bilateral tubal occlusion
- Male sterilization, also called vasectomy
- Sexual abstinence (i.e., refraining from heterosexual intercourse during the entire period of risk associated with the study treatments, when this is in line with the preferred and usual lifestyle of the subject)

7.5.3.10 Prior and Concomitant Therapy

Throughout the study, there should be no significant changes to a subject's diet or medication schedule unless medically indicated. Investigators may prescribe any concomitant medications or treatments deemed necessary to provide adequate supportive care, except those specified as prohibited medications (Section 7.5.3.10.1). All concomitant medications taken during the study will be recorded in the CRF with indication, dose information, and dates of administration. Any changes to concomitant medication will also be documented.

7.5.3.10.1 Prohibited Medications

To be eligible for the study, subjects must not have used certain medications for the indicated timeframe prior to Screening (Section 7.3.2). The following medications will remain prohibited throughout the conduct of the study:

- Pharmacologic vitamin D metabolites or analogs (e.g. calcitriol, doxercalciferol, and paricalcitol)
- Oral phosphate
- Aluminum hydroxide antacids, acetazolamides, and thiazides
- Bisphosphonate therapy (no use in the 2 years prior to Screening)
- Denosumab therapy (no use in the 6 months prior to Screening)
- Teriparatide therapy (no use in the 2 months prior to Screening)
- Initiation of hormone replacement therapy in post-menopausal women (stable doses prior to study entry acceptable)

- Chronic use of systemic corticosteroids (short courses acceptable if indicated)
- PTH suppressors (e.g. cinacalcet)
- Any other mAb therapy (other than study drug; 90-day washout required)

7.5.3.10.2 Permitted Medications

Other than the medications specifically prohibited in this protocol, subjects may receive concomitant medications as required. If serum 25-hydroxyvitamin D (25(OH)D) levels fall below 20 ng/mL, oral supplementation may be provided. Medications (investigational, prescription, over-the-counter, and herbal) and nutritional supplements taken within the 30 days prior to Screening visit will be reviewed and recorded.

7.5.3.10.3 Concomitant Medications/Therapies

Concomitant medications and therapies will be reviewed and recorded in the subject's CRF at each study visit to the investigational site and during Home Health visits. Medications (investigational, prescription, over-the-counter, and herbal) and nutritional supplements taken during the 30 days prior to Screening will be reviewed and recorded. Therapies (physical therapy, occupational therapy as well as mobility and walking devices, including ankle-foot orthosis, braces, cane, crutches, walker, wheelchair etc.) utilized during the 30 days prior to Screening will also be reviewed and recorded. At each subsequent visit, change in medications and therapies since the previous visit will be recorded. Particular attention will be paid to medications used to control pain associated with XLH including changes in dose and frequency of administration. Evidence of narcotic dependence is exclusionary and may result in subject discontinuation.

7.5.3.11 Adverse Events

All AEs will be recorded from the time the subject signs the informed consent through the final Safety Follow-up TC (as defined in Section 7.4.3.1). The determination, evaluation, reporting, and follow-up of AEs will be performed as outlined in Section 8.5. At each visit, subjects will be asked about any new or ongoing AEs since the previous visit. Assessments of AEs will occur at each visit to the investigational site and at all Home Health Visits.

Clinically significant changes from baseline in physical examination findings, vital signs, weight, clinical laboratory parameters, renal ultrasounds, eGFR, ECHO and ECGs will be recorded as AEs or SAEs, if deemed appropriate by the investigator.

7.5.4 Appropriateness of Measures

Osteomalacia is the hallmark of XLH in adults and results from the underlying mineralization defect due to hypophosphatemia. The transiliac crest bone biopsy is a well-established tool in research and clinical use for evaluating bone tissue in metabolic bone diseases such as renal osteodystrophy, osteomalacia, and osteoporosis. Cellular activity and the amount of bone can be accurately quantified by histomorphometry, providing information

that cannot be obtained through other imaging or biochemical studies (Sullivan et al. 1992). Osteomalacia is characterized by an accumulation of osteoid tissue, reflecting a prolonged and defective mineralization process (Arnstein et al. 1967). Specific histomorphometric parameters chosen to evaluate changes in osteomalacia with KRN23 treatment include osteoid thickness, osteoid surface/bone surface, and osteoid volume/bone volume, which are static parameters providing information about the amount of unmineralized bone (Dempster et al. 2013). In addition, mineralization lag time, a dynamic modeling parameter representing the mean time interval between the formation of osteoid and its subsequent mineralization, will be evaluated (Dempster et al. 2013). These parameters showed improvement but not normalization with oral phosphate and vitamin D therapy in XLH patients (Sullivan et al. 1992), (Glorieux et al. 1980), (Harrell et al. 1985).

Secondary endpoints have been incorporated into the design to support the hypothesis that achieving phosphate homeostasis ameliorates defective bone mineralization and metabolism and reduces the clinical burden of XLH. Secondary variables have been selected to demonstrate an overall improvement in bone health and metabolism to reduce disease burden and support the efficacy of KRN23 in this patient population.

Hypophosphatemia is the cause of the consequent osteomalacia and bone abnormalities that result in clinical manifestations of XLH. Serum phosphorus levels serve as a surrogate for the final goals of therapy, which are to restore skeletal health, thereby reducing the associated pain, fatigue, and fracture risk affecting adult patients with XLH. By positively modulating serum phosphorus, the underlying osteomalacia will be resolved, leading to improved clinical outcomes.

PROs have been included in the design to support clinical efficacy and demonstrate patient benefit from KRN23 treatment. The BPI has been used to evaluate pain in numerous diseases and conditions including cancer bone pain, musculoskeletal pain, and osteoarthritis. Similarly, the BFI has been used to evaluate fatigue in musculoskeletal conditions including osteoarthritis and rheumatoid arthritis.

The safety parameters to be evaluated in this study include standard assessments such as recording of medical history, AEs and SAEs, physical examination, vital signs, serum chemistry, and other routine clinical and laboratory procedures. Additional safety measures have been incorporated into the study design to perform a risk assessment of ectopic mineralization in this population. PK and ADA samples will also be obtained to gain additional information regarding exposure and immunogenicity of KRN23.

7.6 Statistical Methods and Determination of Sample Size

A full description of the analysis details and procedures for handling missing, spurious, or unused data will be provided in the Statistical Analysis Plan (SAP).

7.6.1 Determination of Sample Size

The study will enroll approximately 14 adult subjects with XLH; baseline histologic and histomorphometric assessments of the bone biopsy specimens will be performed as each biopsy is completed to assess sample quality and confirm the presence of osteomalacia in at least 8 subjects. To ensure a level of gender balance, at least 3 subjects of each sex will be enrolled. At least 6 paired biopsy specimens are expected at the end of the study. A reduction in excess osteoid is expected to be shown in all subjects with paired biopsies with an estimated ≥ 50% reduction from baseline in osteoid thickness. The sample size and study duration are believed to be sufficient to enable characterization of KRN23 effects on bone tissue and skeletal health.

Information will be used to support the findings of the larger Phase 3 confirmatory efficacy and safety study, where it is adequately powered to evaluate increases in serum phosphorus levels across the entire dosing interval and to describe the treatment effect on select patient-reported outcomes.

7.6.2 Analysis Populations

7.6.2.1 Primary Analysis Set

The primary analysis set will include enrolled subjects with baseline and follow-up (Week 48/ET) bone biopsy data.

7.6.2.2 Full Analysis Set

The full analysis set for efficacy is defined as all enrolled subjects who receive at least one dose of study drug.

7.6.2.3 Safety Analysis Set

The safety analysis set consists of all enrolled subjects who receive at least one dose of study drug. This analysis set will be used for the analyses of all safety endpoints.

7.6.2.4 Treatment Extension Analysis Set

The treatment extension analysis set consists of all enrolled subjects who continued after the Open-Label Treatment Period and received at least one dose during the Treatment Extension Period I. This analysis set will be used for the analyses of efficacy and safety endpoints.

7.6.3 General Principles

A general description of the statistical methods to be used to analyze the efficacy and safety of the study drug is outlined below. The analyses planned in this protocol will be expanded in the SAP to include detailed description. The SAP will be finalized and approved prior to the

database lock. Any deviations from the analyses described in the protocol and SAP will be noted in the final clinical study report.

The completeness of the data affects the integrity and accuracy of the final study analysis. Therefore, every effort will be made to ensure complete, accurate, and timely data collection, and to avoid missing data. In general, only subjects with a post-baseline efficacy evaluation will be included for that analysis. Missing data will be treated as missing and no statistical imputation method will be used unless stated otherwise. The procedures for handling missing, unused, or spurious data, along with the detailed method for analyses, will be presented in the SAP.

The statistical analyses will be reported using summary tables, figures, and data listings. For continuous variables, the mean, standard deviation, median, quartile, minimum, and maximum will be provided. For discrete data, the frequency and percent distributions will be provided.

7.6.3.1 Subject Accountability

The number of subjects who enrolled, complete the treatment period, and discontinue the study will be summarized. The reasons for study treatment discontinuation and study discontinuation will also be summarized.

7.6.3.2 Demographic and Baseline Characteristics

Demographics (age, gender, race and ethnicity) and other baseline disease characteristics will be summarized using descriptive statistics.

7.6.3.3 Baseline

Generally, for parameters/assessments scheduled to be performed on the same day as the first study treatment, the baseline value is the last value measured before the first administration of study treatment on that day. For parameters/assessments not scheduled to be performed (or scheduled but not performed) on the same day as the first administration of study treatment, the baseline value is the value from the screening period measured closest to the day of first administration of study treatment.

7.6.4 Efficacy Endpoints and Analyses

In general, observed values at baseline and end of study, change from baseline, and percent changes from baseline at end of study will be summarized. The detailed statistical approaches on analysis of primary and secondary endpoints will be described in the SAP.

7.6.4.1 Primary Efficacy Endpoint

The primary endpoint is the percent change from baseline at Week 48 in osteoid volume (osteoid volume/bone volume, OV/BV).

The primary analysis will be performed using the primary analysis set. OV/BV at baseline, 48 Weeks, and the percent change from baseline at 48 Weeks will be summarized.

7.6.4.2 Key Secondary Endpoint

The key secondary efficacy endpoint is the proportion of subjects achieving mean serum phosphorus levels above the LLN (2.5 mg/dL [0.81 mmol/L]) at the mid-point of the dose interval (i.e., Weeks 2, 6, 14, and 22), as averaged across dose cycles between baseline and Week 24.

7.6.4.3 Secondary Endpoints

The secondary efficacy endpoints will compare the effects of treatment with KRN23 on the following:

- Percent changes from baseline in additional histomorphometric parameters – including osteoid thickness (O.Th), osteoid surface/bone surface (OS/BS), and mineralization lag time (MLt)
- Changes from baseline in MAR, MS/BS, BFR and additional measures of bone formation and remodeling
- Additional measures to assess serum phosphorus levels between baseline and Week 24 include:
    - Proportion of subjects achieving mean serum phosphorus levels above the LLN (2.5 mg/dL [0.81 mmol/L] at the end of the dosing cycle (4 weeks after dosing), as averaged across dose cycles
    - Mid-point of dosing cycle: mean change from baseline, and percent change from baseline averaged across dose cycles
    - End of dosing cycle: mean change from baseline, and percent change from baseline averaged across dose cycles
    - Cumulative exposure: area under the curve (AUC)
- Change from baseline over time in serum 1,25$(OH)_2$D, urinary phosphorus, TmP/GFR, and TRP
- Change and percent change from baseline over time in serum bone turnover markers, including P1NP, CTx-I, and BALP

Additional analyses of serum phosphorus including observed values, change from baseline, percent change from baseline over time, and area under the curve will be summarized.

Similar analyses will be performed for 1,25(OH)$_2$D, urinary phosphorus, TmP/GFR, and TRP.

In general, observed values at baseline and end of study, and percent changes from baseline to end of the study for bone quality parameters and bone turnover markers will be summarized.

Correlations may be used to assess the relationship between bone turnover markers and selected histomorphometric indices.

7.6.4.4 Exploratory Endpoints

The following exploratory endpoints will be examined:

- Healing of active pseudofractures and/or fractures, as defined by skeletal survey at baseline and subsequent targeted radiography
- Change from baseline in BPI Pain Severity and in Pain Interference scores over time
- Change from baseline over time in BFI Q3-Worst Fatigue Score
- Change from baseline over time in BFI Global Fatigue score, calculated by averaging all 9 items on the BFI

7.6.5 Safety Analyses

Safety will be evaluated by the incidence, frequency and severity of AEs and SAEs, including clinically significant changes from baseline to scheduled time points in vital signs and weight, physical examinations, eGFR, chemistry, hematology, urinalysis, immunogenicity, and concomitant medications. Additional safety parameters to assess the risk of ectopic mineralization include renal ultrasound, ECHO and ECG findings. Safety analysis will be performed using the safety analysis set.

All AEs will be coded using the Medical Dictionary for Regulatory Activities (MedDRA). The incidence and frequency of AEs will be summarized by System Organ Class, Preferred Term, relationship to study drug, and severity. All reported AEs with onset during the treatment (i.e. treatment-emergent AEs) will be included in the analysis. For each AE, the percentage of subjects who experienced at least 1 occurrence of the given event will be summarized. Special attention will be given to those subjects who died, discontinued treatment due to an AE, or experienced a SAE (e.g. summaries, listings, and narrative preparation may be provided, as appropriate).

Clinical laboratory data will be summarized by the type of laboratory test. Reference ranges and markedly abnormal results (specified in the SAP) will be used in the summary of laboratory data. The frequency and percentage of subjects who experience abnormal clinical laboratory results (i.e. outside of reference ranges) and/or clinically significant abnormalities

(as determined by the investigator) will be presented for each clinical laboratory measurement.

The SAP will provide additional details on the planned safety analyses.

7.6.6 Interim Analysis

Administrative analyses may be performed at any time during the study at the discretion of the Sponsor.

7.6.7 Data Monitoring Committee

An independent DMC constituted for the double-blind, placebo-controlled Phase 3 study (UX023-CL303) includes members with expertise in metabolic bone disease. This DMC will act in an advisory capacity to monitor subject safety for both the UX023-CL303 and UX023-CL304 studies through the end of the Treatment Period (Week 48). A review of safety data will be conducted by the DMC periodically. Ad hoc meetings will be held if indicated based on observed events. The roles and responsibilities of the DMC will be defined in the DMC Charter. During Treatment Extension Periods I and II, safety data will be reviewed by the SSRT on an ongoing basis.

8 STUDY CONDUCT

8.1 Ethics

8.1.1 Institutional Review Board or Ethics Committee

The IRB/EC must be a properly constituted board or committee operating in accordance with 21 CFR Part 56, "Institutional Review Boards." This protocol, any protocol amendments, and the associated informed consent forms (ICFs) must be submitted to the IRB/EC for review and must be approved before screening of any subject into the study. Study drug may not be shipped to the investigator until Ultragenyx or its designee has received a copy of the letter or certificate of approval from the IRB/EC for the protocol and any protocol amendments, as applicable.

All subject recruitment and/or advertising information must be submitted to the IRB/EC and Ultragenyx or its designee for review and approval prior to implementation. IRB/EC approval of any protocol amendments must be received before any of the changes outlined in the amendments are put into effect, except when the amendment has been enacted to protect subject safety. In such cases, the chair of the IRB/EC should be notified immediately and the amendment forwarded to the IRB/EC for review and approval.

8.1.2 Ethical Conduct of Study

This protocol is written in accordance with the principles established by the 18th World Medical Association General Assembly (Helsinki, 1964) and subsequent amendments and clarifications adopted by the General Assemblies. The sponsor and investigator will make every effort to assure the study described in this protocol is conducted in full conformance with those principles, current FDA regulations, ICH Good Clinical Practices (GCP) guidelines, and local ethical and regulatory requirements. Should a conflict arise, the sponsor and investigator will follow whichever law or guideline affords the greater protection to the individual subject. The investigator will also make sure he or she is thoroughly familiar with the appropriate administration and potential risks of administration of the study drug, as described in this protocol and the IB, prior to the initiation of the study.

8.1.3 Subject Information and Consent

Appropriate forms for documenting written informed consent will be provided by the investigator and reviewed and approved by Ultragenyx or its designee before submission to the IRB/EC. Ultragenyx or its designee must receive a copy of the IRB/EC's approval of the ICF before the shipment of study drug to the study site.

It is the investigator's responsibility to obtain signed written informed consent from each potential study subject prior to the conduct of any study procedures. This written informed consent will be obtained after the methods, objectives, requirements, and potential risks of the study have been fully explained to each potential subject. The investigator must explain

to each subject that the subject is completely free to refuse to enter the study or to withdraw from it at any time.

The method of obtaining and documenting informed consent and the contents of the ICF will comply with ICH GCP guidelines, the requirements of 21 CFR Part 50, “Protection of Human Subjects,” the Health Insurance Portability and Accountability Act (HIPAA) regulations, and all other applicable regulatory requirements. Subjects will be given a copy of the signed ICF and will be provided any new information during the course of the study that might affect their continued participation in the study. The investigator or a qualified designee will be available to answer each subject's questions throughout the study, and all of the subject's questions must be answered to the subject's satisfaction. If the protocol is amended and the ICF is revised, each subject will be required to provide written informed consent again using the revised ICF.

Receipt of written informed consent will be documented in each potential subject’s CRF. The signed ICF will remain in each subject's study file and must be available to the study monitor(s) at all times.

8.2 Investigators and Study Administrative Structure

Each investigator must provide Ultragenyx and/or its designee a completed and signed Form FDA 1572 and a Financial Disclosure Form. All sub-investigators must be listed on Form FDA 1572 and Financial Disclosure Forms must be completed for all sub-investigators listed on Form FDA 1572.

Ultragenyx and/or its designee will be responsible for managing and monitoring the clinical trial to ensure compliance with FDA and ICH GCP guidelines. Ultragenyx's trained designated representative (the monitor) will conduct regular visits to the clinical site, to perform source document verification. The monitor will verify the investigator's ongoing qualifications, inspect clinical site facilities, and inspect study records, including proof of IRB/EC review, with the stipulation that subject confidentiality will be maintained in accordance with local and federal regulations, including HIPAA requirements.

A Coordinating Investigator will be identified for multicenter trials. The Coordinating Investigator will be selected on the basis of active participation in the trial, thorough knowledge of the therapeutic area being studied, and the ability to interpret data.
The Coordinating Investigator will read and sign the Clinical Study Report (CSR).

8.3 Investigational Product Accountability

While at the clinical site, study drug must be stored in a secure limited access location at controlled temperature as described in the IB and according to product packaging.
The storage facility must be available for inspection by the study monitor at any time during the study.

A drug accountability record must be maintained for all study drug received, dispensed, returned, and/or lost during the study. This record must be kept current and made available to the study monitor for inspection. Following the close-out of the study, all unused study drug must be returned to Ultragenyx and/or its designee unless other instructions have been provided for final disposition of the study drug.

8.4 Data Handling and Record Keeping

8.4.1 Case Report Forms and Source Documents

The investigator is required to initiate and maintain, for each subject, an adequate and accurate case history that records all observations and other data related to the study for that subject. A validated electronic data capture (EDC) system will be used for entry of the data into electronic CRFs. Data must be recorded on CRFs approved by Ultragenyx or its designee. All information recorded on CRFs for this study must be consistent with the subject's source documentation.

Initial data entry and any changes to the data will be made only by Ultragenyx-authorized users, and data entries and changes will be captured in an electronic audit trail. An explanation of any data change should be recorded in the CRF. All data entered in to the CRF must be verifiable; therefore, CRFs will be routinely checked for accuracy, completeness, and clarity and will be cross-checked for consistency with source documents, including laboratory test reports and other subject records by Ultragenyx or its designee. The investigator must allow direct access to all source documents.

8.4.2 Data Quality Assurance

Monitoring and auditing procedures developed by Ultragenyx and/or its designee will be implemented to ensure compliance with FDA and ICH GCP guidelines. Ultragenyx's designated representative (the monitor) will contact the investigator and conduct regular visits to the study site. The monitor will be expected and allowed to verify the investigator's qualifications, to inspect clinical site facilities, and to inspect study records, including proof of IRB/EC review, with the stipulation that subject confidentiality will be maintained in accordance with local and federal regulations, including HIPAA requirements. The monitor will also be responsible for confirming adherence to the study protocol, inspecting CRFs and source documents, and ensuring the integrity of the data. CRFs will be checked for accuracy, completeness, and clarity and will be cross-checked for consistency with source documents including progress notes, laboratory test reports and other subject records. Instances of missing or uninterruptable data will be resolved in coordination with the investigator.

The monitor will also investigate any questions concerning adherence to regulatory requirements. Any administrative concerns will be clarified and followed. The monitor will maintain contact with the site through frequent direct communications with the study site by e-mail, telephone, facsimile, and/or mail. The investigator and all other site personnel agree

to cooperate fully with the monitor and will work in good faith with the monitor to resolve any and all questions raised and any and all issues identified by the monitor.

The investigator understands that regulatory authorities, the IRB/EC, and/or Ultragenyx or its designees have the right to access all CRFs, source documents, and other study documentation for on-site audit or inspection and will retain this right from the start of the study to at least two years after the last approval of a marketing application or for at least two years after clinical development of the study drug for the indication being studied has been discontinued. The investigator is required to guaranty access to these documents and to cooperate with and support such audits and inspections.

8.4.3 Record Retention

All study records must be retained for at least 25 years after the end of the clinical trial or in accordance with national law. Subject files and other source data must be kept for the maximum period of time permitted by the hospital, institution or private practice, but not less than 25 years. Ultragenyx must be notified and will assist with retention should the Investigator/institution be unable to continue maintenance of subject files for the full 25 years. All study records must be stored in a secure and safe facility.

8.5 Reporting and Follow-up of Adverse Events

8.5.1 Definition of Adverse Events

An AE is defined as any untoward medical occurrence associated with the use of a drug in humans, whether or not considered drug related. An AE can therefore be any unfavorable and unintended sign (including an abnormal laboratory finding, for example), symptom, or disease temporally associated with the use of a medicinal (investigational) product, whether or not related to the medicinal (investigational) products.

A suspected adverse reaction is any AE for which there is a reasonable possibility that the drug caused the AE. For the purposes of expedited safety reporting, “reasonable possibility” means there is evidence to suggest a causal relationship between the drug and the AE. Suspected adverse reaction implies a lesser degree of certainty about causality than adverse reaction, which means any AE caused by a drug.

Life-threatening AE or life-threatening suspected adverse reaction is an AE or suspected adverse reaction that, in the view of either the investigator or Ultragenyx, places the patient or subject at immediate risk of death. It does not include an AE or suspected adverse reaction that, had it occurred in a more severe form, might have caused death.

An AE or suspected adverse reaction is considered “unexpected” if it is not listed in the current Investigators Brochure’s Reference Safety Information (RSI) or is not listed at the specificity or severity that has been observed.

An SAE or serious suspected adverse reaction is an AE or suspected adverse reaction that at any dose, in the view of either the investigator or Ultragenyx, results in any of the following outcomes:

- Death
- A life-threatening AE
- Inpatient hospitalization or prolongation of existing hospitalization
- Persistent or significant incapacity or disability (substantial disruption of the ability to conduct normal life functions)
- A congenital anomaly/birth defect
- Note that hospitalizations planned prior to study enrollment (e.g. for elective surgeries) are not considered SAEs. Hospitalizations that occur for pre-existing conditions that are scheduled after study enrollment are considered SAEs.

Important medical events that may not result in death, be immediately life-threatening, or require hospitalization may be considered serious when, based upon appropriate medical judgment, they may jeopardize the patient or subject and may require medical or surgical intervention to prevent one of the outcomes listed in the definition.

8.5.2 Severity of Adverse Events

Wherever possible, the severity of all AEs will be graded using the NCI CTCAE version 4.03. The majority of AEs can be graded using this system.

If an AE cannot be graded using the CTCAE criteria, it should be graded as mild, moderate, severe, life-threatening, or death using the following definitions.

- Mild (Grade 1): Awareness of signs or symptoms, but easily tolerated and of a minor irritant type, causing no loss of time from normal activities. Symptoms do not require therapy or a medical evaluation; signs and symptoms are transient.
- Moderate (Grade 2): Events introduce a low level of inconvenience or concern to the participant and may interfere with daily activities, but are usually improved by simple therapeutic measures; moderate experiences may cause some interference with functioning.
- Severe (Grade 3): Events interrupt the participant’s normal daily activities and generally require systemic drug therapy or other treatment; they are usually incapacitating.
- Life-threatening (Grade 4): Events that place the participant at immediate risk of death or are disabling.
- Death (Grade 5): Events that result in death.

To make sure there is no confusion or misunderstanding of the difference between the terms "serious" and "severe," which are not synonymous, the following note of clarification is

provided. The term "severe" is often used to describe the intensity (severity) of a specific event (as in mild, moderate, or severe myocardial infarction); the event itself, however, may be of relatively minor medical significance (such as severe headache). This is not the same as "serious" which is based on subject/event outcome or action criteria usually associated with events that pose a threat to a subject's life or functioning. Seriousness (not severity) serves as a guide for defining regulatory reporting obligations.

8.5.3 Relationship of Adverse Events to Study Drug

The investigator will assess the potential relationship of the AE to study drug using the following descriptions.

Categories of attributions for "Unrelated" events:

- ***Unrelated:*** This category applies to an AE that ***is clearly not related*** to the investigational agent/procedure, beyond a reasonable doubt.
- ***Unlikely Related:*** This category applied to an AE that ***is doubtfully related*** to the investigational agent/procedure.

Categories of attributions for "Related" events:

- ***Possibly Related:*** This category applies to an AE that ***may be related*** to the investigational agent/procedure.
- ***Probably Related:*** This category applies to an AE that ***is likely related*** to the investigational agent/procedure.
- ***Definitely Related:*** This category applies to an AE that ***is clearly related*** to the investigational agent/procedure.

For the purposes of reporting to regulatory agencies, AEs deemed as Definitely, Probably or Possibly Related will be considered Related and those deemed Unrelated or Unlikely Related will be considered Unrelated.

8.5.4 Adverse Event Reporting to Ultragenyx

8.5.4.1 General

All AEs (i.e. any new or worsening in severity or frequency of a preexisting condition) with onset after the subject signs consent for study participation must be promptly documented on the AE eCRF via the EDC system. The Principal Investigator is responsible for evaluating all AEs, obtaining supporting documents, and ensuring documentation of the event is adequate. Details of the AE must include severity, relationship to study drug, duration, and outcome.

All AEs will be collected from the time the subject signs informed consent through the final Safety Follow-up TC (as defined in Section 7.4.3.1). In addition, the Investigator should report any AE that occurs after this time period that is believed to have a reasonable possibility of being associated with study drug.

AEs ongoing at the final Safety Follow-up TC should have a comment in the source document by the Investigator whether the event has recovered, recovered with sequelae, or stabilized.

8.5.4.2 Serious Adverse Events, Serious Adverse Drug Reactions, and Requirements for Immediate Reporting

Any SAE that occurs at any time during the study, including a clinically significantly abnormal laboratory test result that is considered serious, must be reported within 24 hours of knowledge of the event to Ultragenyx or its designee. These requirements apply equally to all subjects, regardless of the study phase or the at-risk subject's treatment assignment or dosage. The reporting requirement for SAEs is from the time of signing of the ICF through the final Safety Follow-up TC.

SAEs will be reported by completing and submitting SAE report forms to Ultragenyx or its designee. Initial SAE reports must be followed by detailed descriptions. These should include copies of hospital case records and other documents when requested. Telephone reports must be confirmed promptly by facsimile. Follow-up SAE information must be submitted in a timely manner as additional information becomes available. All SAEs regardless of relationship to study drug must be followed to resolution or stabilization if improvement is not expected.

A death occurring during the study, during the per-protocol follow-up period, or through the final Safety Follow-up TC (as defined in Section 7.4.3.1) must be reported to Ultragenyx or its designee within 24 hours of knowledge of the death whether or not it is considered treatment-related.

The investigator also must notify the IRB/EC of the occurrence of the SAE, in writing, as soon as is practicable and in accordance with IRB/EC requirements and local law. A copy of this notification must be provided to Ultragenyx or its designee.

8.5.4.3 Urgent Safety Reporting

The regulations governing clinical studies state that the sponsor and investigator are required to take appropriate urgent safety measures to protect subjects against any immediate hazards that may affect the safety of subjects, and that the appropriate regulatory bodies should be notified according to their respective regulations. According to the European Union (EU) Clinical Trial Directive 2001/20/EC, "…in the light of the circumstances, notably the occurrence of any new event relating to the conduct of the trial or the development of the investigational medicinal product where that new event is likely to affect the safety of the subjects, the sponsor and the investigator shall take appropriate urgent safety measures to

protect the subjects against any immediate hazard. The sponsor shall forthwith inform the competent authorities of those new events and the measures taken and shall ensure that the Ethics Committee (EC) is notified at the same time." The reporting period for urgent safety issues is the period from the signing of the ICF through the final Safety Follow-up TC (as defined in Section 7.4.3.1). Investigators are required to report any urgent safety measures to Ultragenyx within 24 hours.

8.5.4.4 Adverse Drug Reaction Reporting

Ultragenyx or its designee will submit suspected unexpected serious adverse reactions (SUSAR) to appropriate Regulatory Authorities (including Competent Authorities in all Member States concerned), Ethics Committees, and Investigators as per local laws and regulations. Fatal and life-threatening SUSARs will be submitted no later than 7-calendar days of first knowledge of the event and follow-up information submitted within an additional eight (8) days. All other SUSARs will be submitted within 15-calendar days of first knowledge of the event.

Principal Investigators are required to report any urgent safety matters to Ultragenyx or its designee within 24 hours. Ultragenyx or its designee will inform the Regulatory Authorities, Ethics Committees, and Investigators of any events (e.g. change to the safety profile of KRN23, major safety findings) that may occur during the clinical trial that do not fall within the definition of a SUSAR but may affect the safety of subjects participating in the clinical trials, as required, in accordance with applicable laws and regulations. The reporting period for urgent safety issues is the period from the signing of the ICF through the final Safety Follow-up TC (as defined in Section 7.4.3.1).

The investigator will notify the IRBs/Research Ethics Boards (REB)/ECs of SAEs and urgent safety matters, in accordance with IRB/REB/EC requirements and local laws and regulations. A copy of this notification must be provided to Ultragenyx or its designee.

Non-SUSARs will be maintained in the Ultragenyx safety database and provided in annual and/or periodic reports as per local laws and regulations. Ultragenyx or its designee will prepare and submit annual safety reports and/or other aggregate periodic summary reports to Regulatory Authorities and Ethics Committees, as per local laws and regulations.

8.5.4.5 Pregnancy in Subject or Partner

Pregnancies in subjects or partners must be reported within 24 hours of knowledge of the event to Ultragenyx or its designee. The reporting period for pregnancies is the period from the signing of the ICF through the final Safety Follow-up TC (as defined in Section 7.4.3.1). Reported pregnancy of a subject or a subject's partner, while participating in the study, will be monitored for the full duration and/or followed until the outcome of the pregnancy is known. In the event of a pregnancy in the partner of a subject, the Investigator should make every effort to obtain the female partner's consent for release of protected health information. Refer to the Study Reference Manual for details on the reporting

procedures to follow in the event of pregnancy. Pregnancy-associated SAEs will be processed and submitted, as necessary, as per the SUSAR reporting process (Section 8.5.4.4).

8.5.4.6 Review of Safety Data

The Ultragenyx SSRT, Medical Monitor, and Investigator(s) will actively review safety data during the course of the study. Subsequent safety data review by the SSRT will occur approximately quarterly or more frequently, as needed. Safety data for review will, at a minimum, include listings of all SAEs, treatment-emergent AEs, grade 2 or greater laboratory values, deaths, and AEs leading to study discontinuation.

To facilitate prompt risk mitigation activities, the SSRT will immediately evaluate:

- Any observations that may materially influence the risk-benefit analysis of KRN23
- Any significant safety issues that have been identified from cumulative data
- Any actions taken by any country's Regulatory Authority due to safety issues

Potential safety signals identified during the SSRT reviews or any other process during the conduct of the study will be escalated to the appropriate internal Ultragenyx safety governing bodies. Any action indicated by Ultragenyx safety governing bodies will be communicated accordingly to all stakeholders, e.g. Regulatory Authorities, Ethics Committees, and Investigators.

An independent DMC will act in an advisory capacity to monitor subject safety through the end of the Treatment Period (Week 48). The DMC may meet on a routine basis, or as needed, to review aggregate safety data and provide advice regarding the safety of subjects and the continuing scientific validity of the study. The DMC may also be asked to review SUSARs that represent changes in the nature or an increase in the frequency of events and may provide recommendations regarding continued subject participation.

Potential safety signals identified during the DMC reviews or any other process during the conduct of the study will be escalated to the appropriate internal Ultragenyx safety governing bodies. Any action indicated by Ultragenyx safety governing bodies will be communicated accordingly to all stakeholders, e.g. Regulatory Authorities, ECs, IRBs, and Investigators.

8.5.4.7 Safety Contact Information

Drug Safety	Medical Monitor
PrimeVigilance<br>[redacted]	Michael Skinner, MD, PharmD<br>Premier Research<br>[redacted]

8.6 Financing and Insurance

Financing and insurance for this clinical trial will be addressed in clinical trial agreements with the study site.

8.7 Publication Policy

Any publication or presentation by the investigator and/or the Institution based on data or results resulting from the Ultragenyx study shall only be done in strict accordance with the Publication section in the Clinical Trial Agreement executed between Ultragenyx or its designee and the Institution and/or the investigator.

9 REFERENCES

Aono, Y, Hasegawa, H, Yamazaki, Y, Shimada, T, Fujita, T, Yamashita, T and Fukumoto, S. 2011. "Anti-FGF-23 neutralizing antibodies ameliorate muscle weakness and decreased spontaneous movement of Hyp mice." *J Bone Miner Res* 26 (4):803-10.

Aono, Y, Yamazaki, Y, Yasutake, J, Kawata, T, Hasegawa, H, Urakawa, I, Fujita, T, Wada, M, Yamashita, T, Fukumoto, S and Shimada, T. 2009. "Therapeutic effects of anti-FGF23 antibodies in hypophosphatemic rickets/osteomalacia." *J Bone Miner Res* 24 (11):1879-88.

Arnstein, AR, Frame, B and Frost, HM. 1967. "Recent progress in osteomalacia and rickets." *Ann Intern Med* 67 (6):1296-330.

Burnett, CH, Dent, CE, Harper, C and Warland, BJ. 1964. "Vitamin D-Resistant Rickets. Analysis of Twenty-Four Pedigrees with Hereditary and Sporadic Cases." *Am J Med* 36:222-32.

Carpenter, TO, Imel, EA, Holm, IA, Jan de Beur, SM and Insogna, KL. 2011. "A clinician's guide to X-linked hypophosphatemia." *J Bone Miner Res* 26 (7):1381-8.

Chalmers, J, Conacher, WD, Gardner, DL and Scott, PJ. 1967. "Osteomalacia--a common disease in elderly women." *J Bone Joint Surg Br* 49 (3):403-23.

CTFG. 2017. *Clinical Trial Facilitation Group. Recommendations related to contraception and pregnancy testing in clinical trials* 2014 [cited January 2017]. Available from http://www.hma.eu/fileadmin/dateien/Human_Medicines/01-About_HMA/Working_Groups/CTFG/2014_09_HMA_CTFG_Contraception.pdf.

Dempster, DW, Compston, JE, Drezner, MK, Glorieux, FH, Kanis, JA, Malluche, H, Meunier, PJ, Ott, SM, Recker, RR and Parfitt, AM. 2013. "Standardized nomenclature, symbols, and units for bone histomorphometry: a 2012 update of the report of the ASBMR Histomorphometry Nomenclature Committee." *J Bone Miner Res* 28 (1):2-17.

Dixon, PH, Christie, PT, Wooding, C, Trump, D, Grieff, M, Holm, I, Gertner, JM, Schmidtke, J, Shah, B, Shaw, N, Smith, C, Tau, C, Schlessinger, D, Whyte, MP and Thakker, RV. 1998. "Mutational analysis of PHEX gene in X-linked hypophosphatemia." *J Clin Endocrinol Metab* 83 (10):3615-23.

Fukumoto, S. 2008. "Physiological regulation and disorders of phosphate metabolism--pivotal role of fibroblast growth factor 23." *Intern Med* 47 (5):337-43.

Glorieux, FH, Marie, PJ, Pettifor, JM and Delvin, EE. 1980. "Bone response to phosphate salts, ergocalciferol, and calcitriol in hypophosphatemic vitamin D-resistant rickets." *N Engl J Med* 303 (18):1023-31.

Goodyer, PR, Kronick, JB, Jequier, S, Reade, TM and Scriver, CR. 1987. "Nephrocalcinosis and its relationship to treatment of hereditary rickets." *J Pediatr* 111 (5):700-4.

Harrell, RM, Lyles, KW, Harrelson, JM, Friedman, NE and Drezner, MK. 1985. "Healing of bone disease in X-linked hypophosphatemic rickets/osteomalacia. Induction and maintenance with phosphorus and calcitriol." *J Clin Invest* 75 (6):1858-68.

Imel, EA and Econs, MJ. 2005. "Fibroblast growth factor 23: roles in health and disease." *J Am Soc Nephrol* 16 (9):2565-75.

Jonsson, KB, Zahradnik, R, Larsson, T, White, KE, Sugimoto, T, Imanishi, Y, Yamamoto, T, Hampson, G, Koshiyama, H, Ljunggren, O, Oba, K, Yang, IM, Miyauchi, A, Econs, MJ, Lavigne, J and Juppner, H. 2003. "Fibroblast growth factor 23 in oncogenic osteomalacia and X-linked hypophosphatemia." *N Engl J Med* 348 (17):1656-63.

Kang, YE, Hong, JH, Kim, J, Joung, KH, Kim, HJ, Ku, BJ and Kim, KS. 2014. "A Novel PHEX Gene Mutation in a Patient with Sporadic Hypophosphatemic Rickets." *Endocrinol Metab (Seoul)* 29 (2):195-201.

Keller, S, Bann, CM, Dodd, SL, Schein, J, Mendoza, TR and Cleeland, CS. 2004. "Validity of the brief pain inventory for use in documenting the outcomes of patients with noncancer pain." *Clin J Pain* 20 (5):309-18.

Levey, AS, Stevens, LA, Schmid, CH, Zhang, YL, Castro, AF, 3rd, Feldman, HI, Kusek, JW, Eggers, P, Van Lente, F, Greene, T, Coresh, J and Ckd, EPI. 2009. "A new equation to estimate glomerular filtration rate." *Ann Intern Med* 150 (9):604-12.

Linglart, A, Biosse-Duplan, M, Briot, K, Chaussain, C, Esterle, L, Guillaume-Czitrom, S, Kamenicky, P, Nevoux, J, Prie, D, Rothenbuhler, A, Wicart, P and Harvengt, P. 2014. "Therapeutic management of hypophosphatemic rickets from infancy to adulthood." *Endocr Connect* 3 (1).

Liu, S, Tang, W, Zhou, J, Vierthaler, L and Quarles, LD. 2007. "Distinct roles for intrinsic osteocyte abnormalities and systemic factors in regulation of FGF23 and bone mineralization in Hyp mice." *Am J Physiol Endocrinol Metab* 293 (6):E1636-E44.

Mendoza, TR, Wang, XS, Cleeland, CS, Morrissey, M, Johnson, BA, Wendt, JK and Huber, SL. 1999. "The rapid assessment of fatigue severity in cancer patients: use of the Brief Fatigue Inventory." *Cancer* 85 (5):1186-96.

Murphy, SL, Alexander, NB, Levoska, M and Smith, DM. 2013. "Relationship between fatigue and subsequent physical activity among older adults with symptomatic osteoarthritis." *Arthritis Care Res (Hoboken)* 65 (10):1617-24.

Murphy, SL, Lyden, AK, Smith, DM, Dong, Q and Koliba, JF. 2010. "Effects of a tailored activity pacing intervention on pain and fatigue for adults with osteoarthritis." *Am J Occup Ther* 64 (6):869-76.

Patriquin, H and Robitaille, P. 1986. "Renal calcium deposition in children: sonographic demonstration of the Anderson-Carr progression." *AJR Am J Roentgenol* 146 (6):1253-6.

Perwad, F, Azam, N, Zhang, MY, Yamashita, T, Tenenhouse, HS and Portale, AA. 2005. "Dietary and serum phosphorus regulate fibroblast growth factor 23 expression and 1,25-dihydroxyvitamin D metabolism in mice." *Endocrinology* 146 (12):5358-64.

PHEXdb. *PHEX Locus Database, Search Engine Site* [cited November 2014. Available from http://www.phexdb.mcgill.ca/.

Razzaque, MS and Lanske, B. 2007. "The emerging role of the fibroblast growth factor-23-klotho axis in renal regulation of phosphate homeostasis." *J Endocrinol* 194 (1):1-10.

Reid, IR, Hardy, DC, Murphy, WA, Teitelbaum, SL, Bergfeld, MA and Whyte, MP. 1989. "X-linked hypophosphatemia: a clinical, biochemical, and histopathologic assessment of morbidity in adults." *Medicine (Baltimore)* 68 (6):336-52.

Rowe, PS. 2012. "Regulation of bone-renal mineral and energy metabolism: the PHEX, FGF23, DMP1, MEPE ASARM pathway." *Crit Rev Eukaryot Gene Expr* 22 (1):61-86.

Sabbagh, Y, Tenenhouse, HS and Econs, MJ. 2008. "Mendelian Hypophosphatemias." In *The Online Metabolic and Molecular Bases of Inherited Disease*, edited by C. R. Scriver, Childs and W. S. Sly. New York, NY: McGraw-Hill.

Shikino, K, Ikusaka, M and Yamashita, T. 2014. "Vitamin D-deficient osteomalacia due to excessive self-restrictions for atopic dermatitis." *BMJ Case Rep* 2014.

Sullivan, W, Carpenter, T, Glorieux, F, Travers, R and Insogna, K. 1992. "A prospective trial of phosphate and 1,25-dihydroxyvitamin D3 therapy in symptomatic adults with X-linked hypophosphatemic rickets." *J Clin Endocrinol Metab* 75 (3):879-85.

Tenenhouse, H and Econs, M. 2001. "Mendelian Hypophosphatemias." In *The metabolic and molecular basis of inherited disease, 8th ed.*, edited by C. B. Scriver, AL; Sly, WS; Valle, D, 5039–67. New York: McGraw-Hill.

Veilleux, LN, Cheung, M, Ben Amor, M and Rauch, F. 2012. "Abnormalities in muscle density and muscle function in hypophosphatemic rickets." *J Clin Endocrinol Metab* 97 (8):E1492-8.

Verge, CF, Lam, A, Simpson, JM, Cowell, CT, Howard, NJ and Silink, M. 1991. "Effects of therapy in X-linked hypophosphatemic rickets." *N Engl J Med* 325 (26):1843-8.

Wolfe, F. 2004. "Fatigue assessments in rheumatoid arthritis: comparative performance of visual analog scales and longer fatigue questionnaires in 7760 patients." *J Rheumatol* 31 (10):1896-902.

Yamazaki, Y, Okazaki, R, Shibata, M, Hasegawa, Y, Satoh, K, Tajima, T, Takeuchi, Y, Fujita, T, Nakahara, K, Yamashita, T and Fukumoto, S. 2002. "Increased circulatory level of biologically active full-length FGF-23 in patients with hypophosphatemic rickets/osteomalacia." *J Clin Endocrinol Metab* 87 (11):4957-60.

Yamazaki, Y, Tamada, T, Kasai, N, Urakawa, I, Aono, Y, Hasegawa, H, Fujita, T, Kuroki, R, Yamashita, T, Fukumoto, S and Shimada, T. 2008. "Anti-FGF23 neutralizing antibodies show the physiological role and structural features of FGF23." *J Bone Miner.Res* 23 (9):1509-18.

10 SIGNATURE PAGE

Protocol Title: An Open-Label, Single-Arm, Phase 3 Study to Evaluate the Effects of KRN23 on Osteomalacia in Adults with X-linked Hypophosphatemia (XLH)

Protocol Number: UX023-CL304 Amendment 3

I have read Protocol UX023-CL304. I agree to conduct the study as detailed in this protocol and in compliance with the Declaration of Helsinki, Good Clinical Practices (GCP), and all applicable regulatory requirements and guidelines.

Investigator Signature Date

Printed Name: ______________________________

Accepted for the Sponsor:

As the Sponsor representative, I confirm that Ultragenyx will comply with all Sponsor obligations as detailed in all applicable regulations and guidelines. I will ensure the [redacted]

Javier San Martin, MD Date
Vice President, Clinical Sciences
Ultragenyx Pharmaceutical Inc.
